# Functional and Transcriptome Analysis Reveals an Acclimatization Strategy for Abiotic Stress Tolerance Mediated by *Arabidopsis* NF-YA Family Members

**DOI:** 10.1371/journal.pone.0048138

**Published:** 2012-10-31

**Authors:** Marco Antonio Leyva-González, Enrique Ibarra-Laclette, Alfredo Cruz-Ramírez, Luis Herrera-Estrella

**Affiliations:** Laboratorio Nacional de Genómica para la Biodiversidad (LANGEBIO), Centro de Investigación y Estudios Avanzados Irapuato, Irapuato, Guanajuato, México; RIKEN Plant Science Center, Japan

## Abstract

Nuclear Factor Y (NF-Y) is a heterotrimeric complex formed by NF-YA/NF-YB/NF-YC subunits that binds to the CCAAT-box in eukaryotic promoters. In contrast to other organisms, in which a single gene encodes each subunit, in plants gene families of over 10 members encode each of the subunits. Here we report that five members of the *Arabidopsis thaliana NF-YA* family are strongly induced by several stress conditions *via* transcriptional and miR169-related post-transcriptional mechanisms. Overexpression of *NF-YA2*, *7* and *10* resulted in dwarf late-senescent plants with enhanced tolerance to several types of abiotic stress. These phenotypes are related to alterations in sucrose/starch balance and cell elongation observed in NF-YA overexpressing plants. The use of transcriptomic analysis of transgenic plants that express miR169-resistant versions of *NF-YA2*, *3*, *7*, and *10* under an estradiol inducible system, as well as a dominant-repressor version of NF-YA2 revealed a set of genes, whose promoters are enriched in NF-Y binding sites (CCAAT-box) and that may be directly regulated by the NF-Y complex. This analysis also suggests that NF-YAs could participate in modulating gene regulation through positive and negative mechanisms. We propose a model in which the increase in *NF-YA* transcript levels in response to abiotic stress is part of an adaptive response to adverse environmental conditions in which a reduction in plant growth rate plays a key role.

## Introduction

Nuclear factor Y (NF-Y) (also termed Heme Activator Protein or CCAAT Binding Factor) is a heterotrimeric transcription factor (TF) composed of NF-YA (HAP2/CBF-B), NF-YB (HAP3/CBF-A) and NF-YC (HAP5/CBF-C) subunits, which is conserved from yeast to mammals. In yeast (*Saccharomyces cerevisiae*) there is a fourth subunit, HAP4 necessary for complex formation and transcriptional activation [1], [2]. NF-Y activates transcription by recognizing the CCAAT- box cis-acting element present in about 30% of eukaryotic promoters in a highly specific manner via the DNA binding domain present in the NF-YA subunit [3]. The activator activity of NF-Y by binding to the CCAAT boxes has been documented for cytochrome and nitrogen metabolism-related genes in yeast [4], [5] and in genes involved in penicillin biosynthesis in *Aspergillus nidulans* [6]. In animals a multitude of genes, including housekeeping and inducible genes, developmentally controlled and cell cycle-related genes [7], [8], [9] contain CCAAT boxes in its promoter regions. In the case of plants, the CCAAT-box is present mainly in the promoters of genes involved in photosynthesis [10], [11].

Unlike fungi and mammals, which have only a single gene for each subunit, in plants there are more than 10 different loci encoding each subunit [12], suggesting a vast potential for the formation of different NF-Y heterotrimeric complexes that could be involved in a wide range of developmental processes and/or responses to environmental cues. In *Arabidopsis*, 10 *NF-YA*, 13 *NF-YB* and 13 *NF-YC* genes encode the different subunits of NF-Y. The expression of *NF-YA* family members in *Arabidopsis* has been reported to be regulated at the transcriptional level [12], [13] and some *NF-YA* family members, such as *NF-YA2* and *NF-YA5*, are targets of the miR169 family [13], [14]. At least other six Arabidopsis *NY-FA* transcripts are predicted to be targets of mir169 [14].

The fact that the mir169 family comprises 14 members in *Arabidopsis thaliana* [14] suggests a scenario in which a complex set of regulatory combinations between several *NF-YA* transcripts and the numerous members of the mir169 family might control the expression of diverse groups of genes in response to developmental and/or environmental cues. Indeed, *Arabidopsis NF-YA5*, which is involved in drought tolerance [13], is a target for miR169 and miR169 overexpressing (OE) lines showed a similar phenotype to *nf-ya5* [13], demonstrating the participation of this microRNA in drought stress responses.

Recent studies also suggest the involvement of miR169-dependent *NF-YA* regulation in response to nutrient stress, since some members of this microRNA family are down-regulated by nitrate and phosphate starvation [15] and the expression of some genes encoding NF-YA subunits is up-regulated by phosphate (Pi) and nitrate deprivation as well as during leaf senescence [16]–[18].

Another biological process that depends on the mir169-*NF-YA* interaction is plant growth. Overexpression of *Arabidopsis NF-YA4* causes growth reduction [19]; while mimicry lines, with a reduced level of miR169 and increased *NF-YA* transcript levels, display reduced growth [20]. Other *NF-Y* subunits are also involved in growth control, since *Arabidopsis NF-YB1* overexpression arrests growth of the shoot apical meristem [21], and rice *OsHAP3E* (a *NF-YB9* orthologue) overexpression produces a dwarf phenotype [22]. However, the mechanism by which NF-Y modulates plant growth remains to be elucidated.

Besides the mir169-*NF-YA* regulatory mechanism, several studies have highlighted the importance of the NF-Y complex in plant developmental processes. For example, *Arabidopsis thaliana* mutants in the genes encoding NF-YB9 and NF-YB6, denominated LEAFY COTYLEDON 1 (LEC1) and LEAFY COTYLEDON 1 LIKE (L1L), respectively, produce defective embryos, which fail to acquire desiccation tolerance [23]. The notion that NF-Y complexes regulate large groups of genes involved in developmental processes is illustrated by the finding that LEC1 overexpression leads to the formation of embryo like structures in adult leaves [24] whereas *lec1* produces ectopic development of trichomes on cotyledons [25]. Emerging evidence also indicates that NF-Y plays an important role in the flowering process. In *Arabidopsis*, CONSTANS (CO) interacts *in vivo* with NF-YB and NF-YC subunits, replacing a NF-YA partner, to promote flowering by regulating *FLOWERING LOCUS T* (*FT*) [21]. Additionally, NF-YA1 OE plants are late flowering, without altering *CO* mRNA levels, suggesting that NF-YA1 impairs the formation of the CO/NF-YB/NF-YC complex [21].

Several reports demonstrate the participation of NF-Y in abiotic stress responses. In maize (*Zea mays*) and *Arabidopsis*, overexpression of *NF-YB1* and *NF-YB2*, respectively, promote drought resistance [26]. However, microarray analysis of the transcriptional alterations caused by *NF-YB2* overexpression in *Arabidopsis* did not reveal a clear effect on genes directly related to stress tolerance, but rather on those involved in polysaccharide metabolism.

Here we show that the expression of 5 members of the *Arabidopsis NF-YA* family is induced by nutrients stress and, in contrast to the majority of stress-related transcription factors, its expression window is long-term. Overexpression of *NF-YA* members confers tolerance to diverse abiotic stresses, including drought, flooding, cold and heat. Unexpectedly, we found that at least several members of the NF-YA family act as negative regulators of early stress response genes and modulate overall plant growth through modification of carbohydrate metabolism and cell elongation. We propose that this genetic response conduces the plant to a state of acclimatization. We also propose that NF-YA modulates stress responses mainly by sequestering the NF-YB/NF-YC heterodimers preventing its interaction with other transcription factors that induce the expression stress-responsive genes.

## Results

### Members of the *NF-YA* family are strongly up-regulated by different abiotic stresses

It has been shown that some members of the *NF-YA* family are up-regulated in Pi-deprived plants [16], [27] and analysis of public microarray databases suggests that they are also deregulated by other abiotic stresses, including low nitrogen (N), drought, cold, heat, high glucose and by treatment with the stress-related hormone ABA (https://www.genevestigator.com/gv/plant.jsp). These data indicate that the members of the *NF-YA* family might play a general role in stress responses.

**Figure 1:**
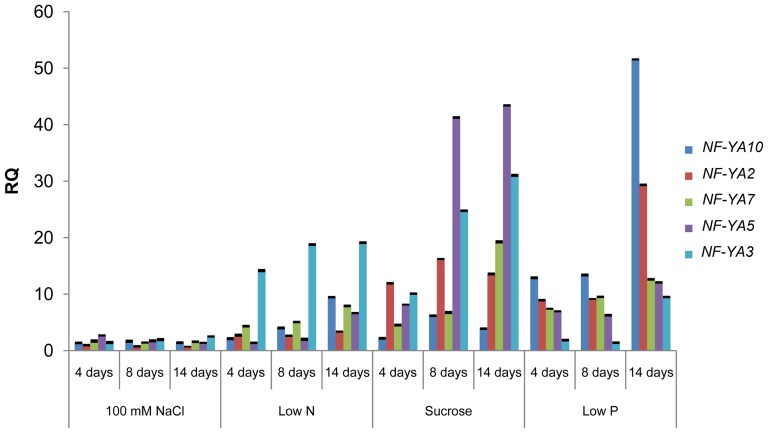
Expression profile of *Arabidopsis NF-YA* genes in seedlings exposed to different stress conditions. Seedlings were germinated in solid media for each treatment (100 mM NaCl, low N, 9.4% Sucrose or low P). Total RNA was extracted after 4, 8 and 14 days of treatment and transcript levels for *NF-YA2*, *3*, *7*, *5* and *10* determined by qRT-PCR. Expression level of *ACTIN 2 (ACT2)* was used as internal reference. Values represent the means and error bars indicate the standard error (SE) of 3 independent amplification replicates.

In the past years our lab has focused on the study of plant responses to nutrient stress, specifically to Pi availability. Therefore, we explore the behavior of several of these *NF-YA* members in response to diverse nutrient stresses. As a starting point we performed a time-course qRT-PCR analysis of the changes in transcript levels of five members of the *NF-YA* family (*NF-YA2*, *3*, *5*, *7*, *10*), which were up-regulated in our microarray analysis of the *Arabidopsis* response to low Pi availability [20]. Besides low Pi (5 µM), we also analyzed its response to low N (50 µM), high salinity (100 mM NaCl) and high sucrose (Suc) (9.4%). Transcript levels for these *NF-YA* family members increased between 5 and 50-fold, under all conditions tested, with the exception of high salinity, where only *NF-YA5* and *NF-YA3* transcript levels increased by 2-fold at 4 and 14 days of treatment, respectively (Figure 1). An increase in *NF-YA2*, *NF-YA7* and *NF-YA10* transcript levels was detected 4 days after germination (dag) in low P, low N and high Suc treatments, *NF-YA5* and *NF-YA3* showed a similar response in high Suc but no change in low N and P conditions, respectively (Figure 1). Transcript levels were maintained 8 dag and showed a further increase at 14 dag for low P and low N. In high Suc, the level of transcript induction was similar at 8 and 14 dag. This experiment also showed that even though all 5 *NF-YAs* tested were up-regulated in response to low P, low N and high Suc conditions, the degree of responsiveness of each *NF-YA* gene to each condition differed, for example, under low N *NF-YA3* was the most responsive (19-fold induction at 14dag), whereas *NF-YA5* was most responsive in high Suc (43-fold induction at 14dag) and *NF-YA10* under low P (51-fold induction at 14 dag).

**Figure 2:**
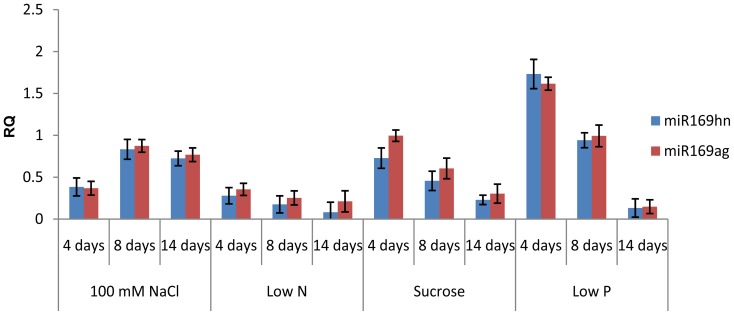
Expression profile of mature miR169 *Arabidopsis* in seedlings exposed to different stress conditions. Seedlings were germinated in solid media for each treatment (100 mM NaCl, low N, 9.4% Sucrose or low P). Total RNA was extracted after 4, 8 and 14 days of treatment and transcript levels for miR169 determined by qRT-PCR. Because of high sequence identity quantification was made for two groups of miR169, a to g and h to n. Expression level of *ACT2* was used as internal reference. Values represents the means and error bars indicate SE of 3 independent amplification replicates.

Since the expression of several members of the *Arabidopsis* miR169 family is repressed by low Pi and low N [15], and *NF-YA5* is a target for this microRNA family [14], it was important to determine whether the expression of the miR169 family is altered by the same nutrient stress conditions under which *NF-YAs* mRNA levels increased. For this, we used a qRT-PCR approach similar to that previously described [15] to examine the mature forms of miR169ag and miR169hn. A more specific analysis was not possible because of the high identity between the mature miR169 sequences. A substantial decrease in the level of the mature miR169ag and miR169hn subgroups was observed in seedlings exposed to low N, low P and high Suc (Figure 2). Reduction in miR169 expression was more pronounced after 14 days of treatment, correlating with the increase in *NF-YA* transcript levels in response to the same treatments. Despite no clear change in expression level was observed for the miR169 subgroups under high salinity conditions, we cannot exclude the possibility that one or few specific members of the miR169 family could be controlling the low but consistent increase in transcript levels detected for *NF-YA3* and *NF-YA5* at 4 and 14 dag (Figure 1). This fact could be due to, the qRT-PCR analysis we used to assess miR169 levels is only able to detect subgroups of several mature miR169s but does not have specificity to detect subtle changes in the expression of a single member of the miR169 family.

Changes in *NF-YA* mRNA levels and miRNA169 abundance fit well with the classical behavior of miRNAs and their targets showing an inverse pattern of expression in which the *NF-YA* transcript level increases as the corresponding miR169 decreases.

### The induction response of *NF-YAs* under Low Pi conditions has both a transcriptional and a post-transcriptional component

**Figure 3:**
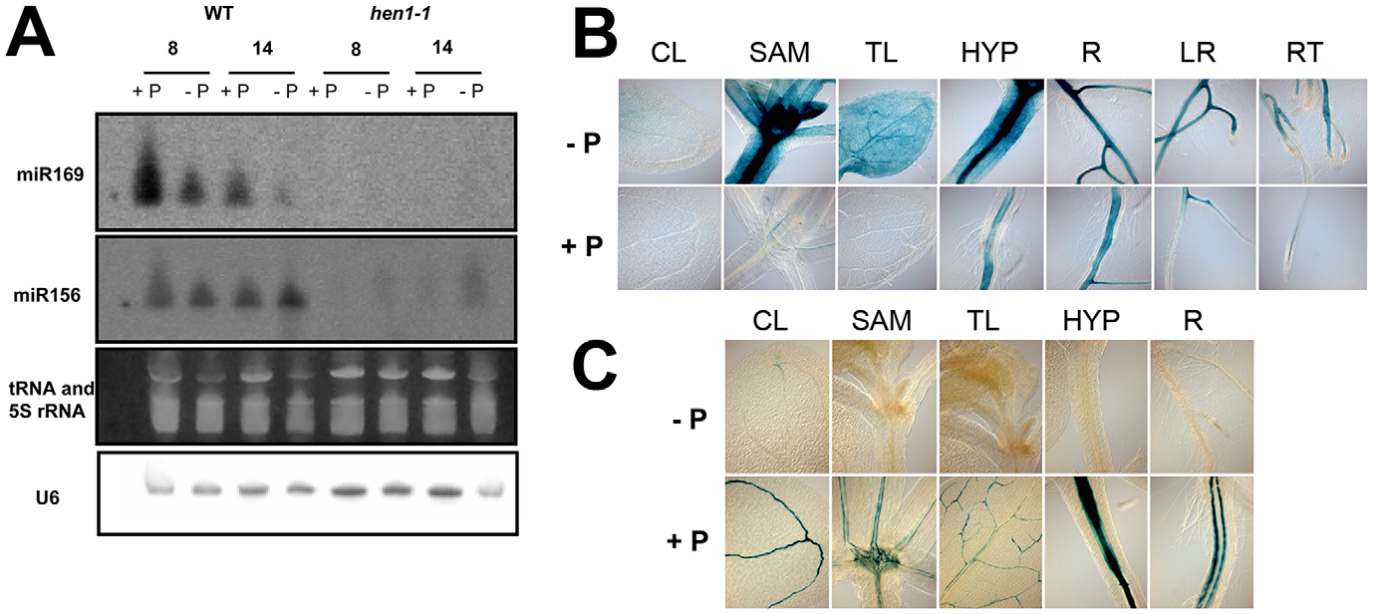
Temporal and Spatial Expression Patterns of miR169 gene and *NF-YA10* in response to Pi availability. (**A**) Northern blot analysis of miR169 in response to Pi availability (+P, 1 mM and −P, 5 µM) in 8 and 14-day-old *hen 1-1* and in WT seedlings. miR156 probe was used as a control to confirm that mature microRNAs in general have decreased levels or are absent in *hen1-1*. U6 probe was used as a loading control. Ethidium bromide-stained tRNA and rRNA is shown below the blots to indicate the relative amount of total low molecular weight RNA loaded per lane. (**B**) and (**C**) Tissue specific expression directed by the *NF-YA10* (**B**) and miR169ij (**C**) promoters as revealed by GUS histochemical assays in seedlings grown in Pi contrasting conditions 14 days after germination (dag). CL, SAM, TL, HYP, R, LR and RT, are cotyledonary leaf, shoot apical meristem, true leaf, hypocotyl, root, lateral root and root tip, respectively.

If the inverse expression patterns of miR169s and its *NF-YA* targets are integrated into stress response regulatory networks, and this obeys a post-transcriptional regulatory program, it would be expected that in mutants affected in microRNA biogenesis, the expression of the *NF-YAs* should be altered. To explore this possibility, we analyzed *NF-YA* transcript levels in *hen 1-1* grown under contrasting Pi conditions. Since HEN1 is involved in transferring a methyl group to the 3′ end of miRNAs that confers stability to the mature miRNA [28], we expect that, in the mutant, the amount of mature miR169 is reduced leading to an increase in the uncleaved *NF-YA* mRNAs. Northern Blot analysis showed that the level of miR169 decreases in WT seedlings subjected to Pi-deprivation for 8 and 14 days, whereas in *hen1-1* the level of mature miR169 was undetectable in both high and low Pi media (Figure 3A). qRT-PCR was performed to determine the effect of *hen1-1* on *NF-YA* transcript levels in media containing sufficient and low Pi. As previously shown for WT seedlings, transcript levels for *NF-YA2*, 3, *5*, *7* and *10* were higher in Pi-deprived seedlings than in those grown in Pi-sufficient conditions (Table 1). In 8-day-old *hen1-1* seedlings grown in Pi sufficient conditions, *NF-YA5*, *NF-YA3*, *NF-YA2* and *NF-YA10* transcript levels were 9-, 5.4-, 15.7- and 5.3-fold higher than those present in WT seedlings, respectively, confirming *NF-YA* post-transcriptional regulation by microRNAs and suggesting that *NF-YA2* is the most tightly post-transcriptionally regulated member of the tested *NF-YAs* (Table 1). In contrast, transcript levels for *NF-YA7*, which lacks the binding site for miR169, was similar in *hen1-1* to that present in WT seedlings (Table 1). In 8-day-old *hen1-1* seedlings grown in low Pi media a further increase in transcript levels was observed for *NF-YA3* and *NF-YA10* in comparison to *hen1-1* seedlings grown in Pi-sufficient conditions (Table 1), suggesting that these genes are also subjected to transcriptional regulation. In the case of *NF-YA2*, transcript levels were similar in Pi-deprived and Pi-sufficient seedlings suggesting regulation mainly at the post-transcriptional level.

**Table 1:** qRT-PCR analysis of the *NF-YA* subfamily en Pi contrasting conditions in WT and *hen 1-1* backgrounds.

Gene	8 days				14 days			
	WT		*hen 1-1*		WT		*hen 1-1*	
	+P	−P	+P	−P	+P	−P	+P	−P
*NF-YA5*	1	6	9	1.2	1	12	**3.3**	**4**
*NF-YA3*	1	1.4	5.4	12.4	1	9.5	**13.6**	**11.9**
*NF-YA2*	1	9	**15.7**	**14.2**	1	29	21	62
*NF-YA10*	1	13	5.3	32.5	1	51	8.9	84.7
*NF-YA7*	1	9.5	0.7	2.5	1	12	0.9	6.8
*SPX1*	1	52	0.5	65	1	169	0.97	73

Transcript levels that reflect a major component of post-transcriptional regulation are in bold. +P, optimum P (1 mM) −P, low P (5 µM).

Similar results were obtained for 14-day-old seedlings for *NF-YA2* and *10*, and for *NF-YA3* and *5* for which transcript levels seem to be mainly regulated post-transcriptionally. To confirm that the effect HEN1 loss of function on *NF-YA* transcript levels was due to a direct effect on microRNA biogenesis and not an indirect general effect on Pi-deprivation responses, we examined the expression of *SPX1*, a Pi-responsive gene [27]. It was found that *SPX1* transcript levels in the WT and *hen1-1* were similar in both Pi-sufficient and limiting conditions, showing that *hen1-1* does not have a general effect on the *Arabidopsis* response to Pi-availability (Table 1).

To corroborate that *NF-YA2*, *3*, *5*, *7* and *10* are also regulated at the transcriptional level by Pi-availability we fused the promoter region of these genes to the *β-glucuronidase* (*GUS*) reporter gene. In Pi-sufficient conditions, a similar expression pattern to that previously reported for *PNF-YAs:GUS* [12] was observed. In contrast, in Pi-deprived seedlings a significant increase of GUS activity was observed for *PNF-YA2*, *3*, *7* and *10* lines with different patterns of tissue specific responses (Figure 3B and Figure S1A to S1D). We also produced *promoter:GUS* transcriptional gene fusions for some of the miR169 family members, namely miR169i/j, miR169k/l, miR169m/n, which are arranged in tandem, and for miR169h and miR169a which are monocistronic units. In seedlings grown in Pi-sufficient media we observed a pattern of expression similar to that previously reported [29], with predominant expression in the vascular tissue. Under Pi-limiting conditions it was observed that GUS staining decreased for miR169 promoters, in particular for miR169h, miR169k/l, and miR169i/j as compared to that observed for seedlings grown in Pi-sufficient media (Figure 3C and Figure S1E to S1H). There is a clear overlap between the expression patterns of *PNF-YA2:GUS*, *PNF-YA5:GUS* and *PNF-YA10:GUS* with *PmiR169h:GUS*, *PmiR169i/j:GUS* and *PmiR169k/l:GUS* in vascular tissues of leaf and hypocotyl; showing a space-time coincidence between the miRNAs and their targets suggesting that, under Pi limiting conditions, the decrease in the transcript levels of these miR169s is, at least in part, responsible for the increase in transcript levels of *NF-YA2*, *NFYA5* and *NF-YA10*.

To confirm that miR169 modulates the level of *NF-YA2*, *3*, *5*, and *10* mRNAs, we generated transgenic *Arabidopsis* lines that overexpress the mature sequence of miR169 h to n, which is identical for all these miRNAs. For this we cloned a fragment of DNA that encodes for miR169m and miR169n precursors arranged in tandem, hereafter called miR169nm, under the control of the CaMV 35S promoter.

Two homozygous overexpressor lines were characterized by qRT-PCR, one showed a 2-fold and the other a 3.5-fold higher level of mature miR169hn when compared to WT plants (Figure S2A). qRT-PCR analysis showed that transcript levels of *NF-YA2*, *3*, *5* and *10* were reduced in both miR169/OE lines, whereas transcript levels for *NF-YA7*, which lacks the target site for miR169, were unaffected by the overexpression of miR169 (Figure S2B).

Taken together, these results confirm that the post-transcriptional regulation of several members of the *NF-YA* family is mediated by miR169hn, which in turn are transcriptionally regulated by Pi availability.

### *NF-YA* overexpression causes a dwarf phenotype

**Figure 4:**
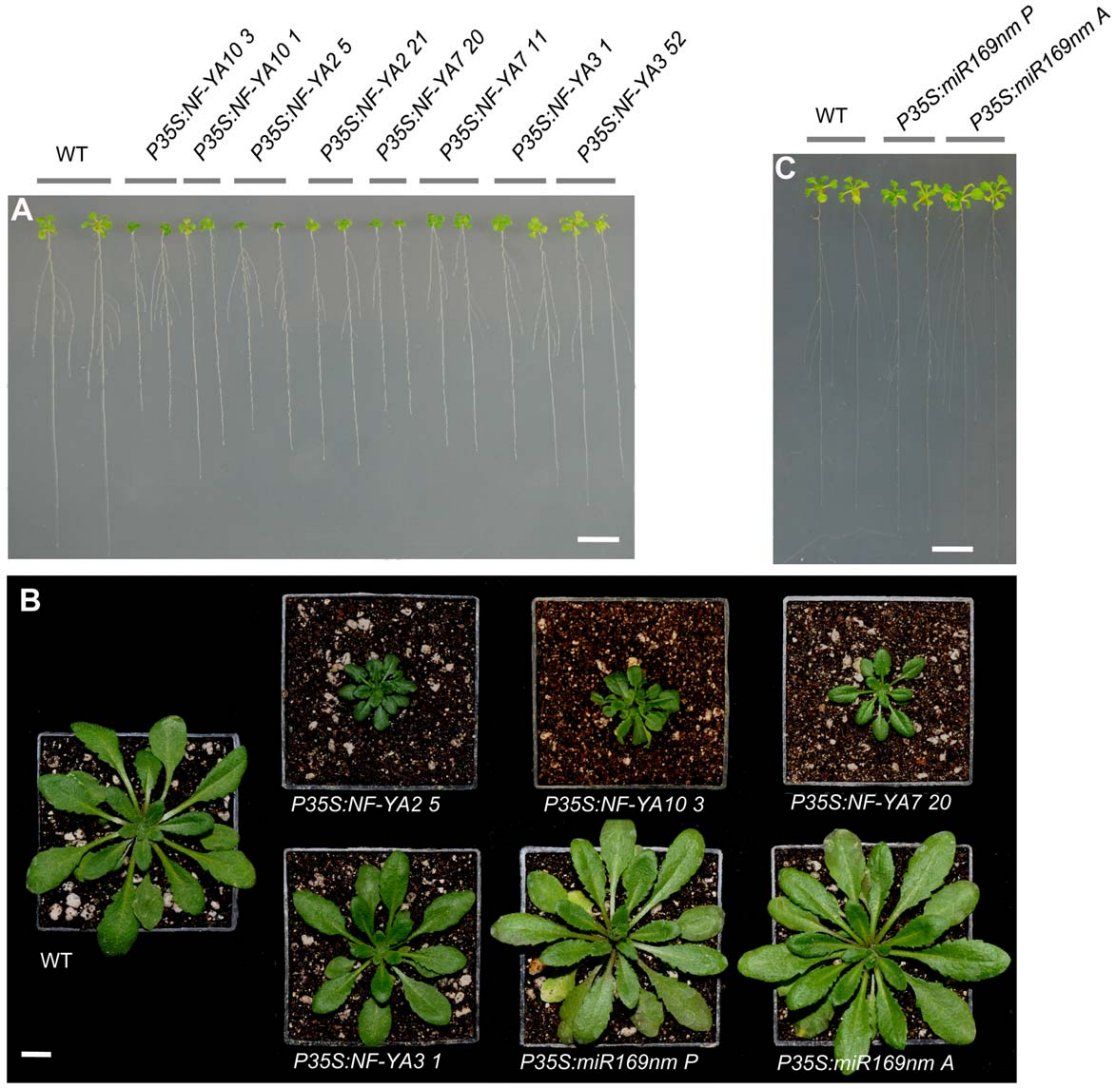
Phenotype of transgenic lines overexpressing *NF-YAs* and miR169nm. (**A**) and (**C**) Phenotype of 14-day-old seedlings overexpressing *NF-YAs* and miR169nm, respectively. (**B**) Phenotype of three-week-old plants grown under greenhouse conditions. Bars = 10 mm in (**A**) to (**C**).

To characterize the functional role of NF-YAs, we produced transgenic plants that express miR169-resistant versions of *NF-YA2*, *3* and *10* genes and the native form of the *NF-YA7* gene, under the control of the CaMV 35S promoter. At least 5 independent overexpressing lines were generated for each construct. After an initial phenotypic evaluation (Figure 4) and qRT-PCR analysis of two representative lines (Figure S3), one OE line for each construct was selected for further detailed evaluation. qRT-PCR analysis showed that these transgenic lines had from 20- up to 1000-fold higher transcript levels than that of the corresponding *NF-YA* in WT plants (Figure S3). No *NF-YA5* OE lines were produced since the effect of its overexpression in *Arabidopsis* was previously reported [13]

Phenotypic analyses of the selected *NF-YA* OE lines showed that *P35S:NF-YA3* plants developed in a similar fashion to WT controls, whereas *P35S:NF-YA2*, *P35S:NF-YA7* and *P35S:NF-YA10* plants showed a dwarf phenotype depending on the expression level of the transgene (Figure 4A and Figure S3). The dwarf phenotype of *P35S:NF-YA2*, *7* and *10* lines was observed both at the seedling and adult stages (Figure 4B), smaller siliques and seeds were also observed in these OE lines compared to those of the WT (Figure S4). The dwarf phenotype observed in *NF-YA* OE lines was reflected in biomass reduction, for example *P35S:NF-YA2* plants had almost 2.5-fold less biomass than the WT (Figure S5). A dark green color was observed in *P35S:NF-YA2*, *7*, and *10* leaves and to a lesser extent in leaves of *P35S:NF-YA3* plants, which correlated with a higher chlorophyll content in these lines as compared to that of WT plants (Figure S6). *P35S:miR169nm* plants had a contrasting phenotype to that observed for *NF-YA* OE lines, as they produced 20% larger seeds, accumulated approximately 25% more biomass and less chlorophyll than WT plants (Figure 4B to 4C, Figure S4, Figure S5, and Figure S6). The relatively mild phenotype observed for the miR169 overexpressing lines could be explained by the fact that repression of *NF-YAs* containing the miR169 target sequences is incomplete and/or because the expression of *NF-YA7* is not affected.

### Transcriptomic analysis of genes downstream of NF-YA2, 3, 7, and 10

To identify genes regulated by NF-YAs while avoiding the potential indirect effects in gene expression related to the dwarf phenotype caused by constitutive *NF-YA* expression, we used the XVE inducible system (LexA-VP16-estrogen receptor) [30], [31] to drive the expression of miR169-resistant forms of *NF-YA2*, *3*, *10* and the native form of *NF-YA7* or the genomic region comprising the mir169nm. Representative transgenic lines containing these gene constructs showed WT phenotype when germinated in media lacking estradiol, whereas in estradiol-containing media, their phenotypes were similar to those observed in their constitutively expressed counterparts (data not shown). Estradiol-inducible *NF-YA* and miRNA169 overexpressing lines were used to analyze the effect of NF-YAs and miR169 overexpression on global RNA profiles.

**Figure 5:**
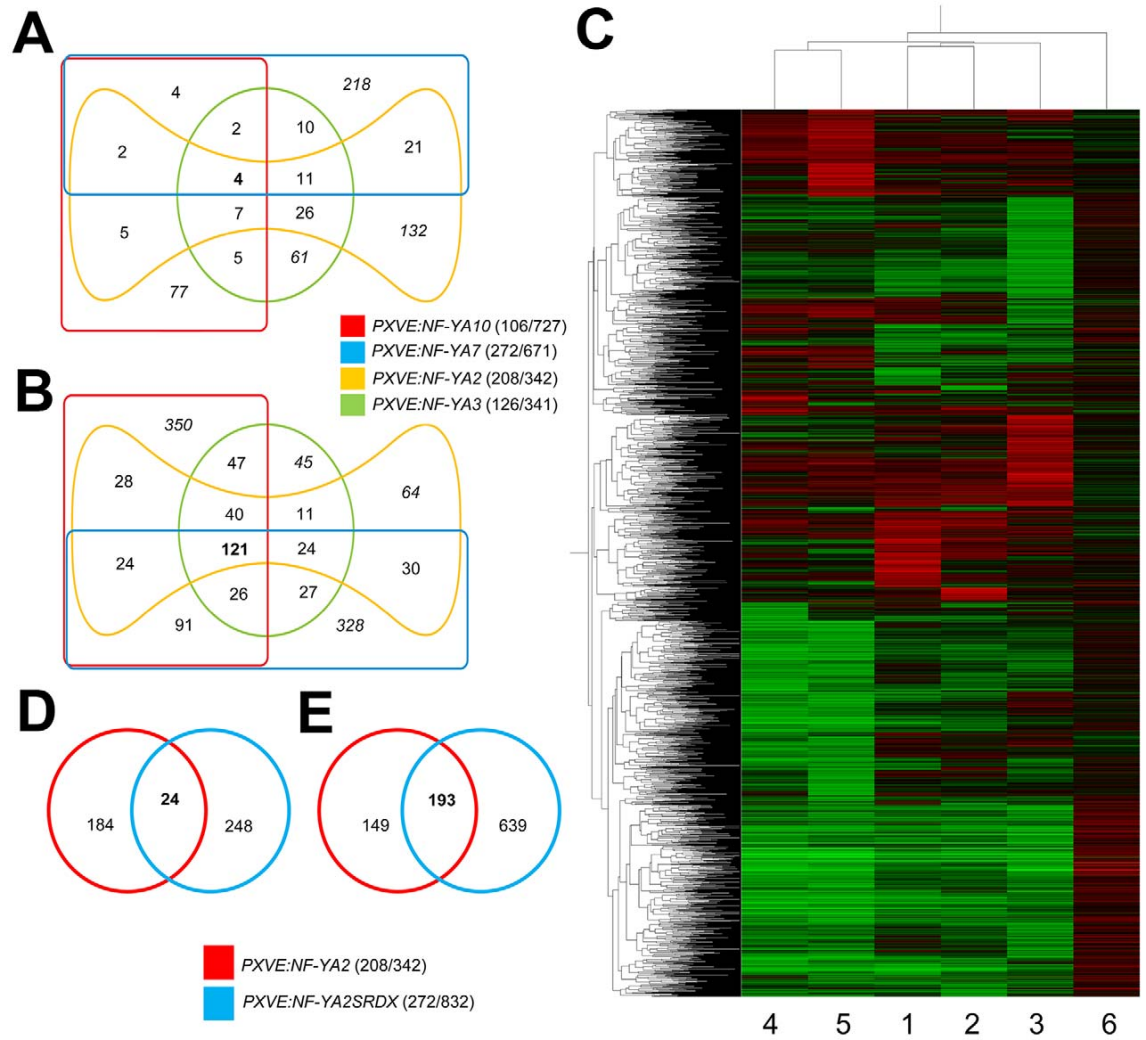
Global gene expression profile in *NF-YA* overexpressing lines. (**A**) and (**B**), (**D**) and (**E**) Venn diagrams showing genes with differential expression for *PXVE:NF-YAs* and *PXVE:NF-YA2SRDX*, respectively. (**A**) and (**D**) represent up-regulated genes; (**B**) and (**E**) down-regulated genes. The number of up-regulated/down-regulated genes is given in parenthesis in each case. The number of shared genes in the corresponding data set is indicated in bold. (**C**) Overall picture of the hierarchical clustering analysis of differentially expressed transcripts. Expression patterns of differential genes identified in at least one of the evaluated lines (*PXVE:NF-YA2*, *3*, *7*, *10*; *PXVE:NF-YA2SRDX* and *PXVE:miRNA169nm*; 1, 2, 3, 4, 5, 6, respectively. Green indicates down-regulated values, red, up-regulated values and black unchanged values.

The microarray data corresponding to the overexpression of *NF-YAs* showed a strikingly small overlap between up-regulated genes (Figure 5A), with only 4 genes shared by the four transgenic lines. Interestingly, down-regulated genes showed a greater overlap with 121 genes common to all lines (Figure 5B). In contrast to the results obtained for *PXVE:NF-YA* lines, our data from the *PXVE:miR169nm* array showed that 270 of the 359 differentially expressed genes were induced, including 32 genes that are down-regulated in the four *PXVE:NF-YAs* lines (Dataset S1). These results suggest that NF-YA overexpression has a mainly negative effect on gene expression and that this repressor activity is modulated by miR169.

**Figure 6:**
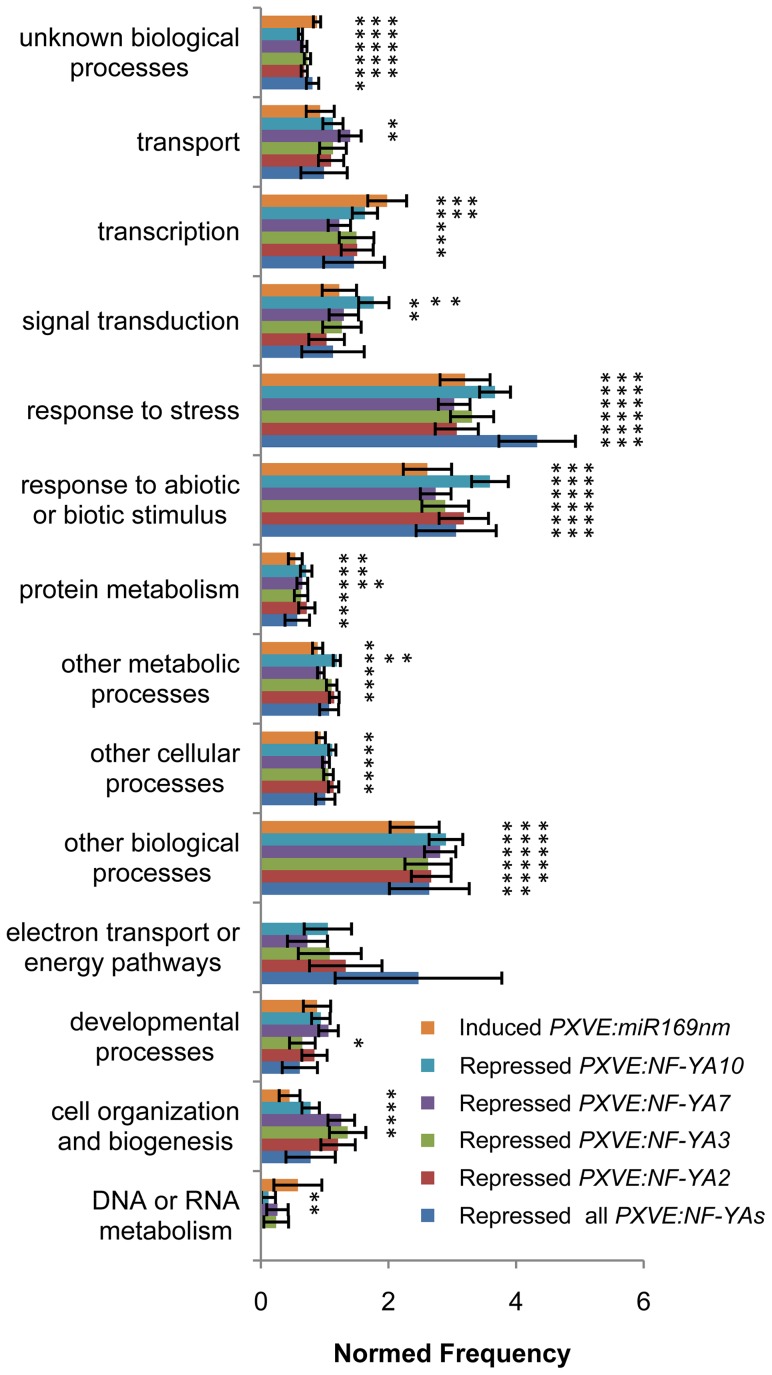
Functional classification of differentially expressed transcripts in *PXVE:NF-YAs* and *PXVE:miR169nm* lines. Gene ontology classification of the transcripts according to classical gene ontology categories using the web-based tool Classification Superviewer (http://bar.utoronto.ca) with normalized class score option. One, two and three asterisks indicate a p-value<0.05, 0.001 and 0.0001, respectively.

To gain insight into the co-regulatory network downstream of NF-YAs, differentially expressed genes in NF-YA overexpressing lines were clustered using the Pearson correlation and average linkage clustering. This analysis confirmed that the expression profiles of *PXVE:NF-YA* lines were similar and inverse to those observed for the *PXVE:miR169nm* line (Figure 5C, Dataset S2). Analysis of the microarray data of *PXVE:NF-YA* lines using the web-tool Superviewer [32], showed that the expression of stress-related genes is modulated by the NF-Y/miR169 system, since these genes were down-regulated as a consequence of *NF-YA* overexpression and induced in the *PXVE:miR169nm* line (Figure 6). Using the MapMan functional category classification we found that differentially expressed genes in NF-YA overexpressing lines include genes whose function is related to carbon metabolism, starch breakdown, glycolysis, cell wall biosynthesis, senescence, fermentation, ABA and auxin sensing, circadian clock, among others, were repressed in *NF-YA* OE lines (Figure S7 and Dataset S3). Moreover, using the web-tool Genesect at VirtualPlant (http://virtualplant.bio.nyu.edu/cgi-bin/vpweb/), we determined that the set of general stress-induced genes [33], [34] have a significant overlap with genes repressed in *PXVE:NF-YA* lines (Table 2).

**Table 2:** Z-score for overlap between early general stress response genes and those repressed in *PXVE:NF-YA* transgenic lines.

Experiment	Line							
	*PXVE:NF-YA2*		*PXVE:NF-YA3*		*PXVE:NF-YA7*		*PXVE:NF-YA10*	
	Z-score	S	Z-score	S	Z-score	S	Z-score	S
Ma and Bohnert (2007)	**18.0**	29	**13.4**	23	**13.9**	34	**23.7**	58
Walley et al. (2007)	**11.2**	17	**12.3**	18	**14.1**	30	**15.7**	35

The overlap of early general stress response genes reported in two previous studies [33], [34] and the repressed genes by NF-YAs (cutoff≤2-fold, p-value≤0.05) is given. Data obtained using the Genesect web tool available through VirtualPlant 1.2 software platform [35]. S, size of intersection. Significant differences (p-value<0.001) based on a Z-score overrepresented (Z-score>11) are highlighted in bold.

### NF-YA expression impairs cell elongation and has a strong impact on carbon metabolism

In coincidence with the dwarf phenotype of the *NF-YA* OE lines, we found that among the differentially expressed genes in *PXVE:NF-YA* lines, a number of genes strongly down-regulated encode for enzymes involved in cell wall modification processes such as xyloglucan endotransglucosylase-hydrolases (XTHs) and expansins (EXPs), suggesting that the dwarf phenotype observed in these lines could be due to defects in cell elongation (Table S1). To determine whether cell elongation was impaired in *P35S:NF-YA* lines, we measured hypocotyl elongation in etiolated *P35S:NF-YA* seedlings. These lines showed an important decrease in elongation capacity in this assay; *P35S:NF-YA2*, *3*, *7* and *10* seedlings had a 49, 33, 41 and 39%, reduction in hypocotyl elongation as compared to that achieved by WT plants (Figure S8A to S8B).

**Figure 7:**
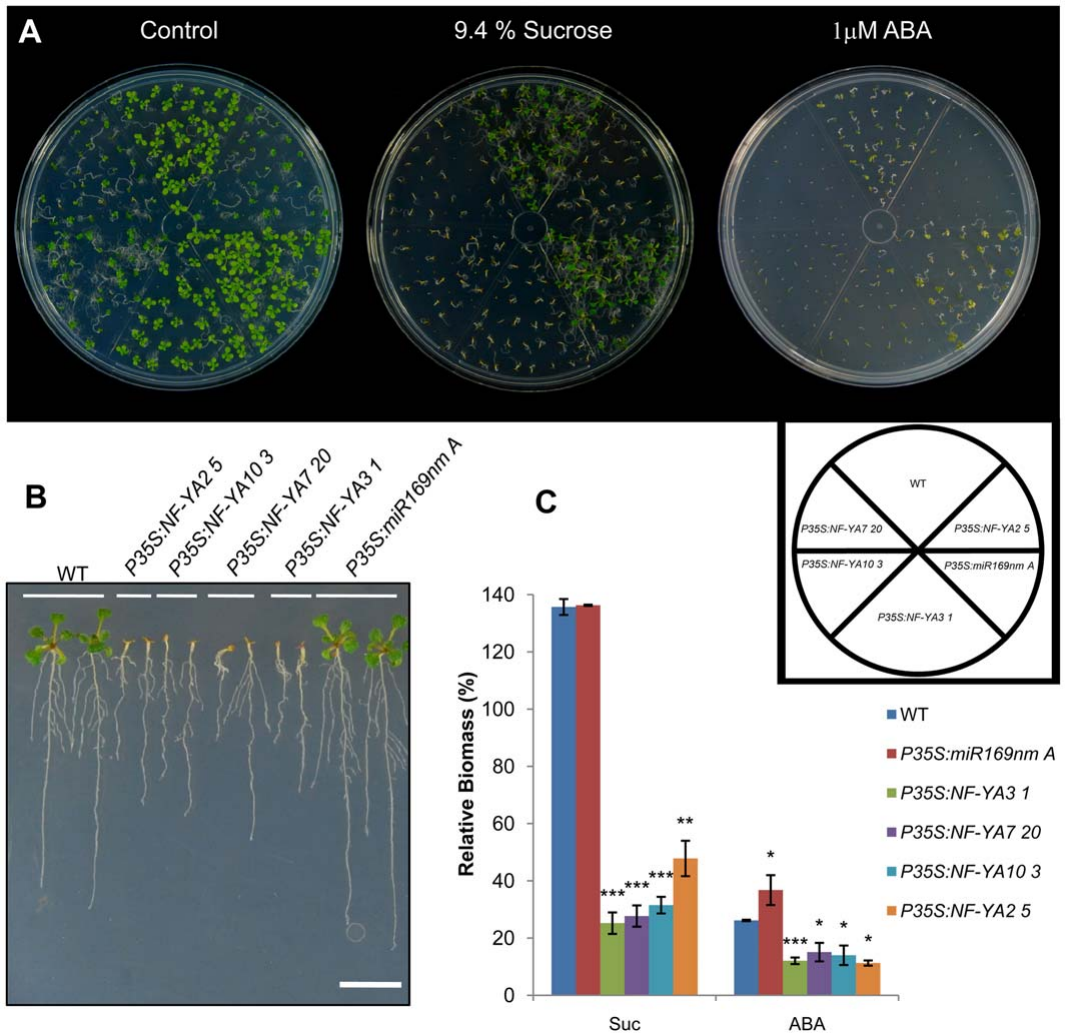
*P35S:NF-YA* lines display an increased sensitivity to sucrose and ABA. (**A**) Images of twelve-day-old *P35S:NF-YA, P35Smir169nm* and WT, seedlings grown on 0.1× MS control media and on media supplemented with 9.4% sucrose or 1 µM ABA under standard light/dark conditions (16/8). A schematic representation of the distribution of the different lines on the plate is shown. (**B**) Phenotype of 14-day-old WT, *P35S:NF-YA* and *P35Smir169nm* seedlings grown in plates with medium supplemented with 9.4% sucrose and grown in vertical position in a growth chamber. Bar = 10 mm. (**C**) Relative biomass of wild-type and overexpressing plants grown on media supplemented with 9.4% sucrose (Suc) or 1 µM ABA (ABA) for twelve days. Total biomass was recorded and used to calculate the relative biomass (expressed as a percent of the value for the same line growing on control medium, set to 100%). Values are means and SD of three biological replicates statistically treated using a student *t*-test (*P<0.05, **P<0.01, ***P<0.001).

In *PXVE:NF-YA* seedlings we also identified several differentially expressed genes involved in carbon metabolism, such as sucrose synthases (*SUS1* and *SUS4*), qua-quine starch (*QQS*), β-amylase 3 (*BAM3*) and *α-AMYLASE 2*. Interestingly, *P35S:NF-YA* lines treated with 9.4% Suc showed a Suc-hypersensitive phenotype, showing limited cotyledon expansion and being unable to develop true leaves as compared to the WT (Figure 7A to 7C). Moreover, we found that *P35S:NF-YA2* plants had a 2-fold higher Suc content than WT whereas miR169/OE lines showed a 20% reduction (Table S2). *P35S:NF-YA* lines also had a higher starch content than WT plants, whereas the *P35S:miR169nm* line showed lower starch accumulation (Figure S9). Together these results suggest that *NF-YAs* can modulate plant growth (i.e. cell expansion) through the regulation of carbon metabolism related genes.

### *P35S:NF-YA* lines have an abiotic stress tolerance phenotype related to altered ABA perception and senescence

Our microarray data also showed that the expression of genes involved in ABA perception was strongly affected in all *PXVE:NF-YA* lines (Table 3). Since the crosstalk between sugar and ABA signaling has been well documented [36], we tested whether *NF-YA* overexpression also alters ABA sensitivity. Seedlings of *P35S:NF-YA* lines grown on plates with 1 µM ABA showed hypersensitivity to this hormone, while the *P35S:miR169nm* line was slightly less sensitive than the WT (Figure 7A and 7C).

**Table 3:** ABA signaling pathway related genes with altered transcript level in *PXVE:NF-YA* transgenic lines.

Gene ID	Description	Fold Change			
		*PXVE:NF-YA2*	*PXVE:NF-YA3*	*PXVE:NF-YA7*	*PXVE:NF-YA10*
At4g17870	PYR1/RCAR11	-	-	0.56	0.61
At1g01360	PYL9/RCAR1	0.61	0.58	0.51	0.50
At2g26040	PYL2/RCAR14	0.52	0.56	0.61	0.58
At2g38310	PYL4/RCAR10	-	-	0.78	0.67
At5g53160	PYL8/RCAR3	0.62	0.56	0.51	0.62
At5g05440	PYL5/RCAR8	-	0.71	0.48	-
At2g40330	PYL6/RCAR9	0.66	0.64	0.60	0.59
At1g72770	HAB1	-	-	-	0.32
At4g33950	OST1	0.61^*^	0.54	0.59	0.31
At3g50500	SNRK2.2	-	-	0.72	-
At2g17290	CPK6	-	-	0.64	0.67
At1g35670	CPK11	0.46	-	0.50	0.45
At3g57530	CPK32	-	-	-	0.46
At3g51920	CML9	-	-	0.58	0.55
At5g37770	CML24	0.62	0.69	0.62	0.53

Gene expression values shown represent fold change (estradiol treatment vs. control), p-value≤0.05. PYR, PYRABACTIN RESISTANCE; PYL, PYR1-Like; RCAR, regulatory component of ABA receptor; HAB1, Hypersensitive to ABA1; OST1, Open stomata 1; SNRK, Sucrose non-fermenting1 related kinase; CPK, calcium-dependent protein kinase; CML, Calmodulin like. Asterisk (*) indicates p-value 0.056. (−) not statistically significant.

**Figure 8:**
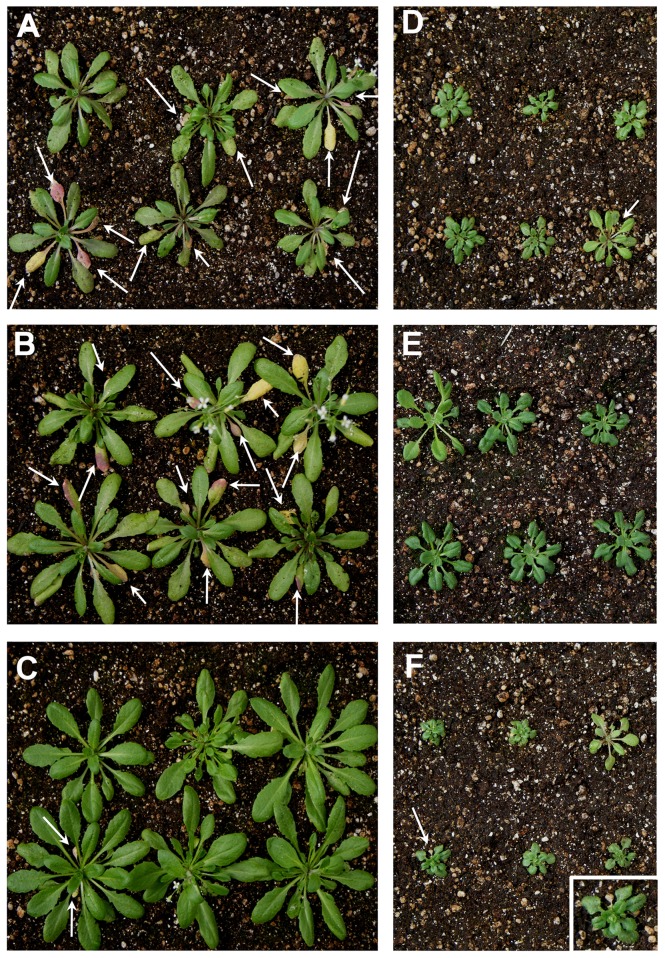
*NF-YA* overexpression produces a flooding tolerant phenotype. (**A**) to (**F**) represent plants belonging to WT, *P35S:miR169nm* and *P35S:NF-YA3*, *7*, *10*, *2*, respectively, after 14 days of recovery. Arrows indicate senescent leaves. Inset in (**F**) is a magnification of *P35S:NF-YA2* rosette. Two-week-old plants of each transgenic line were completely submerged in water-filled tanks in standard light/dark cycle (16/8) for 5 days and photographed after a recovery period of 14 days. The depth of the water column during submergence was kept at 6 cm above the pot surface.

A direct link between ABA and abiotic stress responses has been reported, i.e., transgenic plants hypersensitive to ABA are tolerant to drought stress [37]. According to our microarray data, *NF-YA* overexpression down-regulates the expression of the general stress response genes, including most of the 48 genes of the “anaerobic cluster” [38] (Table S3). These findings prompted us to evaluate the response of *P35S:NF-YA* lines to abiotic stress conditions, namely flooding, drought, heat and cold. *P35S:NF-YA* lines showed higher tolerance to submergence when compared to WT plants, in which leaves showed the typical yellowing characteristic of the senescence induced by hypoxia (Figure 8A, 8C to 8F); *P35S:miR169nm* plants behaved similar to WT (Figure 8B). We also observed that stress-induced damage by drought (Figure S10A and S10B), heat and cold (Figure S11A and S11B) was significantly less severe in *P35S:NF-YA* seedlings than in WT and *P35S:miR169nm* seedlings.

**Figure 9:**
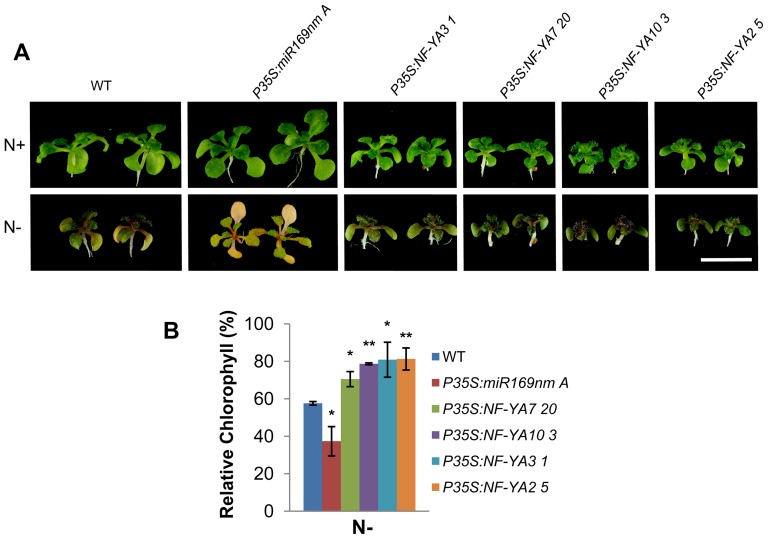
Plants overexpressing *NF-YA* display reduced N starvation induced senescence. (**A**) Photograph of WT, *P35S:NF-YA* and *P35S:miR169nm* plants grown for 2 weeks on N+ (N optimum) or N− (N limited) media under standard light/dark cycle. Bar = 10 mm. (**B**) Relative chlorophyll contents of WT and overexpressing plants grown on N contrasting conditions. Total chlorophyll content was measured, normalized per gram fresh weight of sample and used to calculate the relative content (expressed as a percent of the value for the same line growing on N optimum, set to 100%). Values are means and SD of three independent experiments. Statistically significant differences using the student t-test between WT and transgenic lines are indicated (*P<0.05, **P<0.01).

Several senescence-related genes, including *SENESCENCE 1* (*SEN1*), *POLYUBIQUITIN 10* (*UBQ10*), *SALT TOLERANT ZINC FINGER* (*STZ*) and *WRKY6* are repressed in *P35S:NF-YA* plants (Table 4), suggesting that the increased expression of *NF-YAs* could lead to reduced senescence in *Arabidopsis*. We confirmed the role of NF-YA TFs in controlling senescence using an assay based on nitrogen starvation-induced senescence [39]. After two weeks of N starvation, WT plants showed clear senescence symptoms such as leaf yellowing, particularly in cotyledons. Leaf senescence was less drastic in *P35S:NF-YA2*, *3*, *7* and *10* seedlings than that observed for the WT. In contrast, leaf senescence was more drastic in *P35S:miR169nm* seedlings than for the WT (Figure 9A). Chlorophyll content analysis confirmed the observed phenotypes under N starvation conditions, as chlorophyll content in WT seedlings was reduced 40%, whereas that of *P35S:NF-YA* lines decreased only 20 to 30% and in *P35S:miR169nm* seedlings reduction was about 60% (Figure 9B). Similar results were obtained using another senescence assay, by transferring plants to darkness for 7 days (Figure S12). Under greenhouse conditions, a delay between one to three weeks in developmental senescence was also observed for *NF-YA* overexpressing plants, as compared to the WT at the end of their life cycle (Figure S13A to S13L).

**Table 4:** Expression changes in genes involved in senescence related processes in *PXVE:NF-YA* and *PXVE:miR169nm* transgenic lines.

Gene ID	Description	Fold Change				
		*PXVE:NF-YA2*	*PXVE:NF-YA3*	*PXVE:NF-YA7*	*PXVE:NF-YA10*	*PXVE:miR169nm*
At4g35770	SENESCENCE 1	-	0.48190	0.38967	0.16077	2.21905
At4g30270	SENESCENCE 4	-	-	0.46831	0.37010	1.44568
At4g02380	SENESCENCE-ASSOCIATED GENE 21	0.36220	0.54261	0.41296	-	-
At4g35985	Senescence/dehydration-associated protein-related	-	-	0.41765	0.48309	1.71738
At5g62000	ORESARA 14	-	-	0.60483	0.57416	-
At4g05320	POLYUBIQUITIN 10	0.64522	0.68095	0.54658	0.60331	-
At2g17850	Rhodanese	0.25176	0.11597	0.09108	0.10600	-
At1g27730	SALT TOLERANT ZINC FINGER	0.39096	0.42403	-	0.35361	-
At1g62300	WRKY6	0.62249	0.51744	0.65640	0.39123	1.29049^*^
At4g01250	WRKY22	-	-	0.49892	0.44510	2.13443
At4g23810	WRKY53	0.44315	0.55789	0.58980	-	-
At5g46700	TETRASPANIN 1	-	1.41474	1.50586	-	-
At2g19580	TETRASPANIN 2	-	-	0.59429	0.61269	-
At2g23810	TETRASPANIN 8	-	-	0.45217	0.52539	1.52715
At5g66040	SULFURTRANSFERASE PROTEIN 16	0.66635	0.69143	0.43626	0.64451	-

Gene expression values shown represent fold change (estradiol treatment vs. control), p-value≤0.05. (−), not statistically significant. Asterisk (*) indicates p-value 0.053.

### NF-YAs are part of an acclimatization strategy to cope with adverse conditions

Although there is substantial evidence indicating that growth reduction is part of an adaptive strategy in plants to face abiotic stresses [40], it is also possible that a dwarf phenotype *per se* promotes tolerance to abiotic stresses because smaller plants have lower water and nutrient requirements. To assess whether the stress tolerance phenotype observed in *P35S:NF-YA* is a direct effect of *NF-YA* overexpression *per se* or indirectly by reducing plant growth, we carried out freezing and heat assays using WT, *P35S:NF-YA2* and *PXVE:NF-YA2* lines. Seeds from these three lines were germinated in MS media and 8 dag the seedlings were transferred to MS supplemented with 7 µM β-estradiol for short (24 h) and long (7 days) induction times prior to exposure to freezing and heat stress. The idea is that if overexpression of *NF-YAs per se* is sufficient to confer tolerance, a short induction time should be enough, whereas if growth reduction is responsible for tolerance, a longer induction time will be needed.

**Figure 10:**
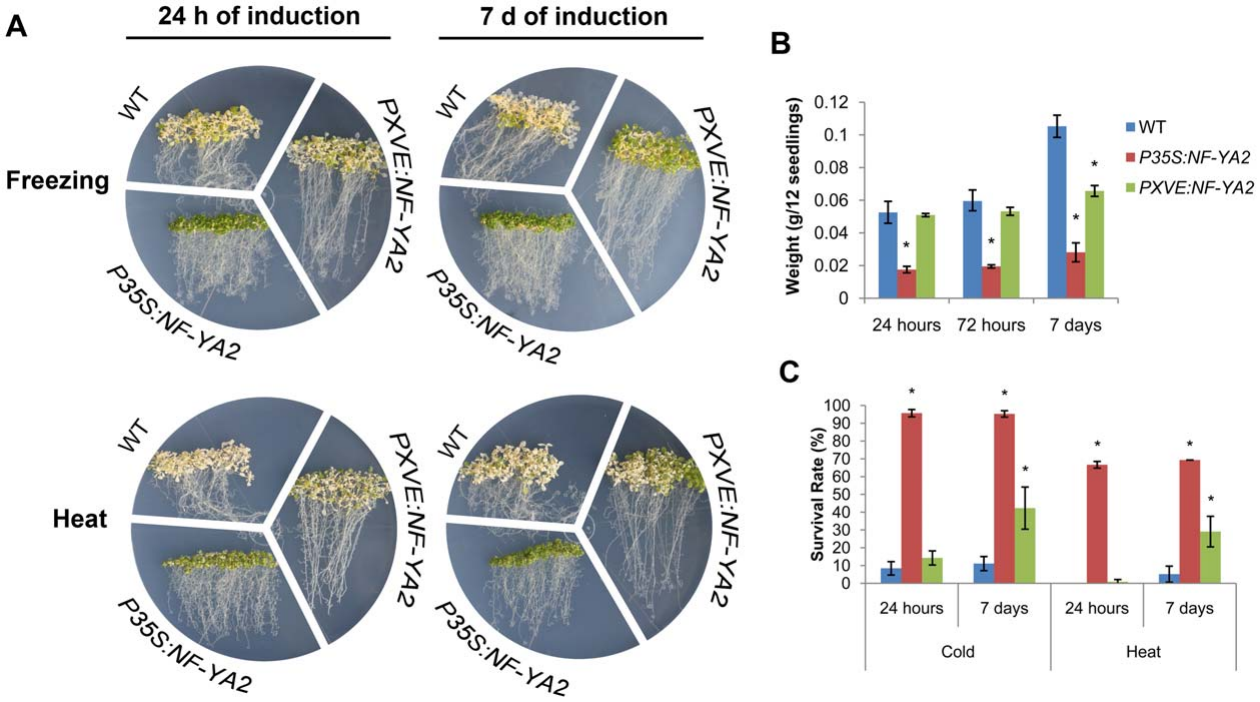
Long-term expression of *NF-YA2* confers tolerance to freezing and heat. (**A**) Images of eight-day-old WT, *P35S:NF-YA2* and *PXVE:NF-YA2* seedlings grown on 0.1× MS that were transferred to 0.1× MS+ β-estradiol (7 µM) for 24 h or 7 days prior to stress. Photographs were taken after four and seven days of recovery time for freezing (−7°C/4 h, lids off) and heat (45°C/2 h, lids on) treatments, respectively. (**B**) Weight of 12 seedlings of each line was determined at the indicated induction times. Data represent means and SD of three replicates statistically treated using a student t-test (*P<0.05). (**C**) Average survival rate of each plant line subsequent to the stress assay (n>50). Data represent means and SD of three replicates statistically treated using a student t-test (*P<0.05).

At the short induction time, freezing and heat conditions imposed a similar stress on *PXVE:NF-YA2* and WT seedlings, (Figure 10A to 10D). After 7 days of induction, when *PXVE:NF-YA2* seedlings show a decrease in growth rate, seedlings showed a better survival to heat and freezing stress compared to WT. The reduction in growth rate was determined by monitoring the repression of *EXL* (Figure S14A and S14B), a gene involved in growth promotion process [41]. A control line constitutively overexpressing NF-YA2 (*P35S:NF-YA2*) performed significantly better than the WT at both short and long exposure times (Figure 10E).

### Promoters of down-regulated genes in *NF-YA* overexpressing lines are enriched in DNA motifs related to stress responses

Since induced ectopic expression of the *NF-YAs* caused changes in the expression of a large number of genes, we searched, using the Athena Web tools [42], for enriched cis-regulatory elements in the promoters of the differentially expressed genes in each line. No clear motif enrichment was detected in up-regulated genes, whereas in down-regulated genes a number of enriched motifs was found, including ABA signaling-related (ABF binding site motif, ABRE binding site motif, ACGT ABRE motif A2OSE), dehydration responses (ABRE-like binding site motif, MYB1AT), drought stress responses (AtMYC2BS in RD22), unfolded protein response (UPRMOTIFIAT), WRKY transcription factor binding sites (W-box), TFIID binding site (TATA-box motif) and motifs related to circadian rhythm (Table S4). Intriguingly, no enrichment of the CCAAT motif in the promoter of differentially expressed genes in *NF-YA* OE lines was observed using either the Athena or the Promomer DNA motif searching tools [43] (Table S5). Therefore, it is unlikely that the complete NF-YA/NF-YB/NF-YC complex, which recognizes the CCAAT-box, regulates the expression of genes that are repressed in *NF-YA* OE lines.

### Transcriptional activities of NF-YAs

Down-regulation of stress related genes in *NF-YA* overexpressing lines could be due to the NF-YA dependent activation of one or several transcriptional repressors or to a direct repressor activity of NF-YAs. In an attempt to elucidate whether NF-YAs act as positive and/or negative regulators of gene expression we used the CRES-T system (Chimeric repressor silencing technology) [44], [45] to convert NF-YA2 into a repressor. We reasoned that if NF-YAs indirectly down-regulated gene expression by activating the expression of one or several transcriptional repressors, conversion of NF-YAs into a repressor would result in the opposite effect (transcriptional activation of the same set of genes in the absence of the repressor(s)). If NF-YAs have a direct negative effect on gene expression, conversion of NF-YAs into repressors should have a similar effect on the expression of genes that are down-regulated in *NF-YA* overexpressing lines. In the CRES-T system, the fusion of a TF to the EAR-motif repression domain (SRDX) causes the repression of the genes whose promoters are normally recognized by the native TF. To avoid possible lethality caused by strong repression of the NF-YAs targets, we cloned the NF-YASRDX fusions under the control of the inducible XVE system.

We obtained at least 5 independent transgenic lines harboring the *PXVE:NF-YA2SRDX* construct. After an initial evaluation of the phenotype of these lines, seeds of two representative lines were germinated in media supplemented with different estradiol concentrations (20 nM to 20 µM). Eight day-old *PXVE:NF-YASRDX2* seedlings showed a strong dwarf phenotype which was proportional to the estradiol concentration (Figure S15A to S15D), while untreated seeds from this line showed normal morphology (data not shown). The phenotype of these NF-YA repressor lines was similar but more drastic than that observed for *NF-YA* overexpressing lines.

Microarray analysis of the *PXVE:NF-YA2SRDX* line showed that the repressor version of NF-YA2 caused a strong down-regulation in gene expression since 832 of the 1104 differentially expressed genes suffered a strong repression (Dataset S1). Comparison of differentially expressed genes between the *PXVE*:*NF-YA2* and *PXVE:NF-YA2SRDX* lines showed little overlap among up-regulated genes (Figure 5D), whereas 56% of repressed genes in *PXVE:NF-YA2* were also repressed in the chimeric repressor line (Figure 5E). Moreover, of 121 down-regulated genes common among the four *PXVE*:*NF-YA* lines, 107 were also repressed in *PXVE:NF-YA2SRDX* (Dataset S1). Clustering array data from the NF-YA2 repressor line using the Pearson correlation and average linkage clustering confirmed that the expression profiles of *PXVE:NF-YA2SRDX* and *PXVE:NF-YA* lines were similar (Figure 5C, Dataset S2). The phenotype of NF-YA2 repressor lines and the similarity of the effect of overexpression of NF-YA2 and its repressor version on global gene expression suggests that NF-YAs could act as repressors. Therefore, it is possible that NF-YAs could act both as transcriptional activators of genes, whose promoters contain the CCAAT-box (as previously documented in other systems) and as repressors of a subset of genes that lack the CCAAT-box. However, formal demonstration of these activator and repressor activities requires additional experimentation.

### Potential NF-YA target genes

Comparison of the microarray data for the *NF-YA2* OE line with that of the chimeric NF-YA2 repressor would allow the identification of the genes that are directly regulated by NF-YA2. We expected that genes whose expression is deregulated (both positively and negatively) in the *NF-YA2* OE line and down-regulated in the *PXVE:NFYA2SRDX* line, would represent direct targets of NF-YA2. With this idea in mind we inspected the list of the differentially expressed genes, whose level of expression varied by at least two-fold (p-value≤0.05), for both *PXVE:NF-YA2* and *PXVE:NFYA2SRDX* lines, and found that out of 832 down-regulated genes in the *PXVE:NFYA2SRDX* line, 202 genes were differentially expressed in *PXVE:NF-YA2*, of which 9 were induced and 193 were repressed. A search for cis-acting elements using the Athena and Promomer web tools in the 193 common genes, showed a similar motif overrepresentation to that obtained for the complete lists of differentially expressed genes (Table S6 and S7).

Because many of the genes that are down-regulated by the chimeric NF-YA2 repressor did not pass the cut off value in the *NF-YA2* OE line, it is possible that these genes represent a group that is directly regulated by the NF-Y complex, but whose expression is not affected by overexpression of only one of the subunits of the complex, considering that the complete NF-YA/NF-YB/NF-YC heterotrimer is necessary to bind CCAAT-box containing promoters [4], [46]. A survey of conserved motifs in the promoters of 322 down-regulated genes in the *PXVE:NF-YA2SRDX* line, whose expression was not significantly altered in *PXVE:NF-YA2* (Dataset S4), revealed that the CCAAT motif was overrepresented in this set of genes (Z-score = 3.4, significance value = 0.001) and that 69% of them (223) have one or more CCAAT boxes (Dataset S4), which represents more than a 2-fold increase compared to the 30% normally found in eukaryotic promoters [3]. These results suggest that NF-YA positively regulates the expression of promoters containing the CCAAT-box and when converted into a repressor down-regulates their expression. Additional experiments, such as transactivation using protoplast systems will be required to provide direct evidence of the activator and repressor activities suggested by our data.

## Discussion

### Expression of *NF-YAs* is regulated at the transcriptional and post-transcriptional level

NF-Y activity is regulated at the transcriptional, post-transcriptional and post-translational levels, with some variation throughout the different kingdoms. In human cells, where the biochemistry of the NF-Y complex is well understood, NF-Y activity is regulated through NF-YA subunit by differential splicing [47], by ubiquitination and acetylation [48] and by long noncoding RNAs [49]. In *Aspergillus nidulans*, NF-Y activity is controlled by the redox status through two closely positioned cysteine residues, Cys74 and Cys78, of the NF-YB subunit [50], but in plants NF-YB subunits lack the first cysteine residue suggesting that this mechanism of regulation does not operate in plants [51].

Our results show that, in *Arabidopsis*, *NF-YA* expression is regulated both at the transcriptional and post-transcriptional levels. Analysis of *NF-YAs* in *hen1-1* and in miR169/OE lines revealed that a post-transcriptional component participates in regulating the expression of *Arabidopsis NF-YA2*, *3*, *5* and *10*. In agreement with the importance of a post-transcriptional mechanism, the expression of miR169 family members showed an inverse expression pattern in response to the same stress conditions that promote an increase in the *NF-YA* transcript levels. Analysis of *promoter-GUS* gene fusions confirmed the transcriptional component of *NF-YA* expression at least in contrasting Pi conditions. This transcriptional induction was not surprising since *NF-YA7* has recently been identified as a direct target of the central regulator of the Pi stress response PHR1 [52].

In plants, the NF-Y complex activity could be controlled by the NF-YA subunit as previously shown for the mammalian system [48], [53]. In agreement with this hypothesis, we found that when NF-YAs are converted into repressor using the CRES-T system the expression of a large set of genes whose promoter contain one or more copies of the CCAAT-box are repressed.

### NF-YA overexpression has a severe impact on growth control processes

Previous reports [17], [54], [55] and our own results show that the expression of several members of the *NF-YA* family is induced under abiotic stress conditions that reduce plant growth. The phenotype and changes in global gene expression observed in our *Arabidopsis NF-YA* OE lines suggest that several NF-YAs could regulate plant growth by modulating the expression of genes involved in carbohydrate metabolism and cell expansion. Carbohydrate metabolism has been proposed to be a key regulator of plant growth, since an imbalance between Suc and starch leads to growth reduction in plants subjected to abiotic stress [54], [56]–[59], also starch content has been proposed as a major integrator of growth control in *Arabidopsis* [60]. The finding that *NF-YA* overexpression leads to a Suc-hypersensitive phenotype, an increase in starch accumulation and a 2-fold increase in Suc content, supports the notion that reduced growth in *P35S:NF-YA* lines is related to alterations in carbohydrate metabolism. We speculate that Suc hyper-accumulation in *P35S:NF-YA2* could be responsible for the repression of genes encoding Suc degrading enzymes such as Sucrose Synthases 1 and 4 observed in all *PXVE:NF-YA* lines.

In this study we found that transcript levels of *BAM3*, *QQS* and *α-AMYLASE 2* are reduced in *PXVE:NF-YA* lines. Interestingly, it has been reported that reduced transcript levels of the starch degrading enzymes qua-quine starch (QQS) and β-amylase 3 (BAM3) result in a sex (starch excess) and dwarf phenotype [61], [62]. These evidences support the notion that NF-YA plays a key role in regulating part of the genetic program that modulates carbon metabolism and plant growth.

Plant growth depends in part on cell elongation, a process that requires re-arrangements of cell wall components where XTHs and EXPs play an active role in cell wall loosening [63]. Several XTH and EXP encoding genes such as *XTH6*, *XTH12*, *XTH14*, *EXP8*, *EXP14* are repressed in several *PXVE:NF-YA* lines, (Table S1) and the seedlings of these lines showed reduced cell elongation (Figure S8A and S8B). When we search for CCAAT enrichment in the promoters of this set of cell wall remodeling genes (Table S1), instead of CCAAT overrepresentation, these promoters displayed an enrichment in TATA box motifs [Promomer: z-score = 4.5 (top), significance = 0.001; Athena: p-value<10^−3^ (the unique highly enriched)], which suggest that cell elongation process would not be directly controlled by the heterotrimeric NF-Y complex.

We provide evidence to suggest that NF-YAs modulate a transcriptional program that links abiotic environmental signals with carbon metabolism and cell expansion, two key elements for plant growth rate. We speculate that the *Arabidopsis* NF-YAs controls a genetic program that, in response to abiotic stresses, adjusts metabolic expenditure associated with cell elongation and carbon metabolism as a strategy to adapt to adverse conditions. This strategy would involve the action of NF-YA factors independently of the capacity of this factor to drive the expression, together with NF-YB and NF-YC, of the CCAAT-containing genes.

### NF-YA has positive and negative effects on gene expression

It has been proposed that NF-Y modulates gene expression by two distinct mechanisms, the best characterized involves the association of the heterotrimeric complex NF-YA/YB/YC to the CCAAT-box motif *via* the DNA-binding domain of NF-YA, inducing the expression of CCAAT-containing genes [53]. Several studies have shown the requirement of the whole NF-YA/YB/YC complex to bind the CCAAT-box and activate the transcription of promoters containing this cis-acting element [4], [19], [46]. The second mechanism proposes that NF-YA acts as a repressor by sequestering the NF-YB/YC heterodimer and preventing its association with other transcription factors that mediate binding to DNA motifs other than the CCAAT-box [21], [64]. Therefore by titrating the NF-YB/YC heterodimer, NF-YA indirectly represses the expression of NF-YB/YC/TF targets.

The finding that the overexpression of the repressor version of NF-YA2 results in the negative regulation of genes whose promoters contain the CCAAT-box, suggests that, as in yeast and animal cells, NF-Y activates the expression of CCAAT-box promoters. However, since overexpression of NF-YA2 also results in the negative regulation of a similar subset of genes that are negatively regulated in the repressor version of NF-YA2, it is likely that NF-YA also acts as a negative regulator of genes expression. NF-YA repressor activity generated by sequestering the NF-YB/YC heterodimer has been previously reported in *Arabidopsis*. It was shown that NF-YB and NF-YC subunits interact with CO to promote flowering and that overexpression of *NF-YA1* and *NF-YA4* delay flowering by preventing the interaction of CO with NF-YB/NF-YC [21]. We found that when *NF-YA2*, *3*, *7* and *10* are ectopically expressed, there is a delayed flowering (Figure S16), suggesting that these NF-YA subunits can act redundantly to NF-YA1 and NF-YA4 and prevent the interaction of CO with the NF-YB/NF-YC heterodimer and alter flowering time. In plants, another clear example of the negative effect of NF-YA on gene expression was reported by Yamamoto and co-workers (2009). These authors showed that NF-YB/NF-YC forms an heterotrimeric complex with bZIP67 to activate the expression of the *CRUCIFERIN C* (*CRC*) promoter *via* binding to an ABRE motif and demonstrated that overexpression of *NF-YA4*, *5*, *7*, and *9* prevents the interaction with bZIP67 and blocks the activation of *CRC* [64]. We found 99, 91, 175 and 285 repressed genes in the microarray analyses of *PXVE:NF-YA2*, *3*, *7*, *10*, respectively, that contain the ABRE or ABRE-like motifs in their promoter sequence (Table S4). This suggests that NF-YA could regulate a large subset of ABA-responsive genes by interfering with the binding of the NF-YB/NF-YC complex to some members of the bZIP family. However, additional experimentation is required to directly demonstrate this general activity of NF-YA as a transcriptional repressor.

### NF-YAs role in controlling a general abiotic stress response

The most conspicuous phenotypes of *NF-YA* OE plants are: a significant reduction in growth rate, delayed senescence and increased tolerance to different abiotic stresses. These phenotypes together with the finding that transcript levels of *NF-YAs* are induced by different abiotic stress conditions, suggest an important general role for NF-Y in the response to adverse environmental conditions in *Arabidopsis*. The relatively late and prolonged induction window of *NF-YAs* by abiotic stress found by others [65] and in this work, contrasts with that observed for more specific regulators of stress responses, which generally have early and transient expression patterns [66].

**Figure 11:**
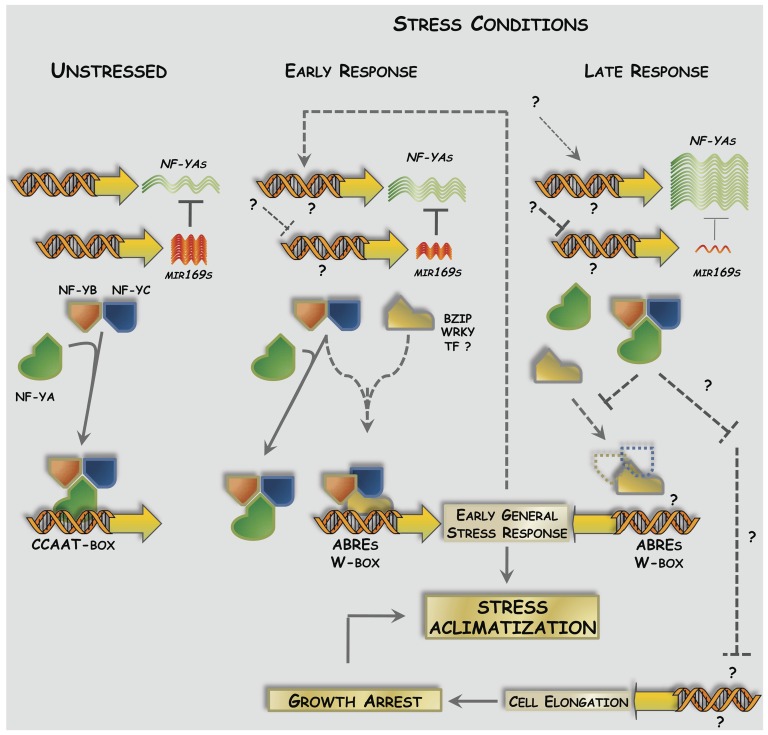
Hypothetical Molecular Model of NF-YA action mode. In WT plants growing under non-stress conditions, the expression of *NF-YAs* is low due miR169-mediated post-transcriptional down-regulation, but sufficient to activate the transcription of CCAAT-box containing promoters. Upon exposure to abiotic stress, *NF-YA* levels increase due to the transcriptional activation of *NF-YA* expression (early) and to the repression of miR169 (late). NF-YAs repress early general stress response genes sequestering NF-YB/NF-YC heterodimer avoiding its interaction probably with bZIPs, and on the other hand, participating in the late down-regulation of cell wall remodeling genes. This last step could be responsible of growth arrest mediated by these TFs when plants face for long time a stressful environment.

Based on our results, we propose a model (Figure 11) in which NF-YAs act as key components of a stress tolerance adaptive mechanism. *NF-YAs* expression is induced in response to prolonged periods of stress. After an initial period of stress, NF-YAs repress the expression of early stress response genes and activate mechanisms that reduce cell elongation and plant growth by repressing genes involved in cell elongation. Reduction in plant growth translates into a reduction in metabolic and energetic costs and water requirements related to cell expansion and plant growth. We also speculate that two different, but not mutually exclusive mechanisms could cause the reduction in cell elongation and growth. One of them related to alterations in carbon partitioning as indicated by the repression of genes involved in these processes and the increased content of Suc and starch in *NF-YA* OE plants and the other related to the repression of genes involved in cell elongation.

Several lines of evidence support our hypothesis: 1) *NF-YA* overexpression represses the early general stress response, which includes the early ABA-mediated induction of gene expression [65], 2) genes involved in cell wall remodeling such as *XTH* and *EXP* are repressed in *NF-YA* OE plants, 3) *NF-YA* OE plants have increased tolerance to senescence induced by different types of environmental stress and 4) *NF-YA* OE plants phenocopy WT plants subjected to abiotic stress in terms of growth rate reduction. Our results suggest that *NF-YA* OE plants enter a kind of quiescent state that leads to an increased tolerance to different types of abiotic stress. This could be similar to the growth reduction already proposed as an adaptive trait that confers tolerance to flooding, in which “the quiescent strategy” functions as a mechanism that protects plants against an unfavorable environment by reducing the cost related to cell expansion and plant growth [67]. The role of growth arrest in abiotic stress tolerance was confirmed with *NF-YA* estradiol-inducible lines, since stress tolerance is not observed upon *NF-YA* induction but rather at later stages when growth arrest is observed.

Mechanistically our hypothetical model proposes that in WT plants growing under non stress conditions, *NF-YAs* expression is low, due to the presence of high levels of miR169, but sufficient to activate the transcription of CCAAT-box containing promoters. Upon exposure to abiotic stress, *NF-YA* levels increase due to their transcriptional activation and to the reduction in miR169 levels. Increased levels of NF-YAs repress early abiotic stress response genes, probably by sequestering the NF-YB/YC heterodimer, creating a regulatory loop to arrest early responses that represent high energy and carbon costs, as well as participating in the activation of late responses. Late abiotic response involves the repression of genes involved in cell wall remodeling and cell elongation, leading to a quiescent state that confers general abiotic resistance. Our hypothetical model is based on solid evidences and led us to generate several interesting working hypothesis for which further experimental work is needed in order to elucidate in detail the molecular mechanisms in which NF-YA seems to play a central role.

## Methods

### Plant Materials and Growth Conditions

Wild-type *Arabidopsis thaliana* (ecotype Col-0) was used to produce all transgenic lines. The *hen1-1* mutant was previously reported [68]. Seeds were surface-sterilized and germinated on agar plates with limiting (5 µM) or sufficient (1 mM) NaH_2_PO_4_ in 0.1× MS medium as previously described [69]. For contrasting nitrogen conditions, N optimum medium contained 2.0 mM NH_4_NO_3_ and 1.9 mM KNO_3_, whereas for N limited conditions, NH_4_NO_3_ was left out of the medium and KNO_3_ was adjusted to 50 µM. For nitrogen starvation induced senescence 1-week-old seedlings grown on N optimum medium were transferred to either N optimum or nitrogen-deficient medium and grown under for 2 weeks. All media (pH 5.7, 1% [w/v] agar) contained 1% Suc, unless stated otherwise.

### Expression Analysis

For qRT-PCR analysis, total RNA was extracted using the RNeasy Plant kit from Qiagen according to the manufacturer's instructions. cDNA templates for PCR amplification were prepared using reverse specific primers (Table S8) and SuperScript III reverse transcriptase (Invitrogen) according to the manufacturer's instructions. qRT-PCR was performed in an ABI PRISM 7500 sequence detection system (Applied Biosystems), using SYBR Green PCR Master Mix according to the manufacturer's protocol. Data shown represent mean values obtained from at least three independent amplification reactions and the error bars indicate ±SE. Gene expression was normalized to that of *ACTIN 2* (*ACT2*) gene by subtracting the CT value of *ACT2* from the CT value of the gene of interest (*NF-YA2*, *3*, *5*, *7*, *10*; *SPX1*). Relative quantification number (RQ) was obtained from the 2^−ΔΔCT^ method, [70] where ΔΔCT represents ΔCT (treatment sample) - ΔCT (control condition). ΔCT was calculated [CT(*NF-YA2*, *3*, *5*, *7*, *10* or *SPX1*)*E]-[CT(*ACT2*)*E] and E is the PCR efficiency according to the protocol reported by Czechowski et al. (2004) [71].

Mature miR169ag and miR169hn levels were determined by qRT-PCR as previously described [72]. Total RNA was extracted using Trizol reagent (Invitrogen) and reverse-transcribed using the stem-loop primers describe by Pant et al., (2009) [15]. Small-RNA Northern blot analysis was performed as described previously [73]. Membranes containing low molecular weight RNA were probed with U6 small nucleolar RNA, miR169 and miR156 oligonucleotides (Table S8) end-labeled in the presence of [γ-^32^P] ATP. The hybridization signals were scanned using a Storm phosphorimager.

### Histological Assays

*NF-YA2*, *3*, *5*, *7*, *10* promoter fusions, and GUS histochemical analysis were carried out as described previously [74]. A genomic region upstream of each ORF was amplified by PCR. For miR169a, miR169h, miR169i/j, miR169k/l and miR169m/n promoter fusions, the promoter sequence immediately upstream to the predicted precursor sequences was amplified and cloned using the protocol mentioned above, except for miR169a which was cloned in the pCR8/GW/TOPO (Invitrogen) vector instead of pDONR221 (Invitrogen).

### Generation of Overexpressing Transgenic Lines

To generate the *P35S:miR169nm* construct, the fragment surrounding the miRNA sequences including both fold-back structures was amplified from genomic DNA. For *P35S::NF-YA* constructs the coding sequence of *NF-YA3*, *10* and *7* was amplified with the primers indicated (Table S8) in order to remove the miR169 target site located at the 3′UTR. The OE lines under the 35S promoter were produced as previously reported [69] except that *NF-YA7* and *10* that were cloned in pCR8/GW/TOPO vector instead of pDONR221. For OE lines under the estradiol inducible system, the same protocol was carried out, but the pMDC7 plasmid was used instead of pb7WG2D (Invitrogen). For *NF-YA2*, a CDS clone [75] (stock # G85154) was obtained from the ABRC and used in the subsequent steps as described [69] using pb7WG2D and pMDC7 vectors to generate 35S and estradiol inducible lines, respectively. Chimeric repressor version for NF-YA2 was produced by amplifying the coding sequence before the stop codon for each gene by PCR with reverse primers containing the SRDX coding sequence and a stop codon coding sequence (Table S8). Transgenic estradiol inducible lines were produced as described early using the pCR8/GW/TOPO and pMDC7 vectors.

### Microarray Analysis

Estradiol inducible lines were grown on liquid media for 7 days in standard light/dark cycle conditions (16/8 h) and on the 8th day, five hours after the light cycle had started, plants were exposed for 24 h to 7 µM β-estradiol or DMSO (mock treatment). Whole seedlings were collected and total RNA was extracted using the RNeasy Kit according to the manufacturer's instructions (Qiagen). Spotted glass microarray slides were obtained from the University of Arizona (http://ag.arizona.edu/microarray/). Three biological replicates for each line were used for RNA isolation and hybridized as two technical replicates (in swap) to the two channel microarrays. Labelling, hybridization, and image processing was performed as described previously [76]. Statistical tests using the Limma software [77] were used to identify differentially expressed genes with at least two-fold change in expression at the end of the estradiol treatment for *PXVE:NF-YAs*, *PXVE:NF-YAsSRDX* and 1.5 -fold in the case of *PXVE:miR169nm* with an adjusted p-value≤0.05 in all cases. The p- value was adjusted with FDR.

### Pigment quantification

Chlorophyll was extracted with DMSO from 100 mg of shoots and Chlorophyll a and b content measured as previously described [78].

### Sucrose analysis

Sucrose amount was quantified by UPLC-MS analysis. 12-day-old shoots were ground in liquid nitrogen, soluble sugar extracted in ethanol 80% (v/v) twice at 65°C for 15 min, samples were centrifuged at 5000 rpm for 10 min. Supernatant was dried under vacuum and resuspended in acetonitrile∶water (1∶1). Sucrose amount was quantified by UPLC-MS analysis performed on an LC-MS system composed of the Waters Acquity UPLC system (Waters Corporation) equipped with an Acquity photodiode array detector interfaced with the ThermoFisher LTQ mass spectrometer (ThermoFisher). Conditions for LC were as follows: column, ACQUITY UPLC BEH Amide 1.7 mm (2.1 mm×100 mm; Waters); solvent A, MeCN∶H_2_O (80∶20); solvent B, MeCN∶H_2_O (30∶70); gradient, 0 to 25 min, initial, 88% A and 12% B; 10 min, 30% A and 70% B; 15 min 88% A and 12% B; flow rate, 0.13 mL·min^−1^; injection volume 5 mL and column temperature, 35°C. Conditions for MS under negative mode were as follows, capillary voltage, 2.4 kV; cone voltage, 100 V; source temperature, 120°C; desolvation temperature, 350°C; cone gas flow, 10 L·h^−1^; desolvation gas flow, 450 L·h^−1^; nebulizer and curtain gas, N_2_.The amount of sucrose was determined by spectrometer software (MassLynx™ v. 4.1, Micromass) using calibration curves prepared with sucrose purchased from Sigma-Aldrich.

### Dark induced elongation assay

Seeds were germinated on 0.1× MS medium for 24 hours and then transferred to darkness for 5 days. Hypocotyl length was determined and Scanning Electron Microscopy was performed in an EVO® 40 Series microscope (Carl Zeiss AG).

### Accession Numbers

Sequence data from this article can be found in the EMBL/GenBank data libraries under the following accession numbers: *NF-YA2* (At3g05690), *NF-YA3* (At1g72830), *NF-YA5* (At1g54160), *NF-YA7* (At1g30500), *NF-YA10* (At5g06510), *SPX1* (At5g20150), *HEN1* (At4g20910), *MIR169a* (At3g13405), *MIR169h* (At1g19371), *MIR169i* (At3g26812), *MIR169j* (At3g26813), *MIR169k* (At3g26815), *MIR169l* (At3g26816), *MIR169m* (At3g26818), *MIR169n* (At3g26819), *MIR156g* (At2g19425), *MIR164a* (At2g47585), *MIR164b* (At5g01747), *MIR164c* (At5g27807), *ACTIN 2* (At3g18780). The microarray data have been deposited in the National Center for Biotechnology Information Gene Expression Omnibus (http://www.ncbi.nlm.nih.gov/geo/) and are accessible through GEO Series accession number GSE36092.

## Supporting Information

Figure S1 **Temporal and Spatial expression pattern of** ***NF-YAs*** **and miR169s in response to Pi availability.**
(PDF)

Figure S2 **qRT-PCR analysis of miR169nm overexpressing lines.**
(PDF)

Figure S3 ***NF-YA*** **expression level in transgenic** ***Arabidopsis*** **lines overexpressing** ***NF-YAs*** **.**
(PDF)

Figure S4 **Ectopic expression of** ***NF-YAs*** **and miR169nm affects seed weight and silique size.**
(PDF)

Figure S5 **Biomass accumulation is affected in** ***P35S:NF-YA*** **and** ***P35S:miR169nm*** **lines.**
(PDF)

Figure S6 **Chlorophyll content of wild-type,** ***P35S:NF-YA*** **and** ***P35S:miR169nm*** **lines.**
(PDF)

Figure S7 **MAPMAN-based functional classification of differentially expressed transcripts in** ***PXVE:NF-YA*** **and** ***PXVE:miR169nm*** **lines.**
(PDF)

Figure S8 **Dark-induced cell elongation is affected in** ***NF-YA*** **overexpressing plants.**
(PDF)

Figure S9 ***NF-YA*** **overexpression causes a starch excess phenotype.**
(PDF)

Figure S10 ***NF-YA*** **overexpressing lines display increased drought tolerance.**
(PDF)

Figure S11 ***NF-YA*** **overexpression enhances heat and freezing tolerance.**
(PDF)

Figure S12 ***NF-YA*** **overexpression delays dark-induced senescence.**
(PDF)

Figure S13 ***NF-YA*** **overexpression prolongs plant longevity.**
(PDF)

Figure S14 **qRT-PCR analysis of WT,** ***PXVE:NF-YA2*** **and** ***P35S:NF-YA2*** **lines subjected to freezing.**
(PDF)

Figure S15 **Dose-dependent effect of estradiol on seedling phenotype of homozygote and heterozygote** ***PXVE:NF-YA2SRDX*** **transgenic lines.**
(PDF)

Figure S16 ***NF-YA*** **overexpression delays flowering time.**
(PDF)

Table S1 **Expression changes in genes involved in cell wall remodeling in** ***PXVE:NF-YA*** **transgenic lines.**
(PDF)

Table S2 **Sucrose content in WT,** ***P35S:NF-YA2*** **and** ***P35S:miR169nm*** **lines.**
(PDF)

Table S3 **Expression changes in genes belonging to the “anaerobic cluster” in** ***PXVE:NF-YA*** **and** ***PXVE:miR169nm*** **transgenic lines.**
(PDF)

Table S4 **Motifs enrichment in deregulated genes in estradiol treated PXVE transgenic lines.**
(PDF)

Table S5 **Z-score for TATA-box and CCAAT-box motifs in the promoters of differentially expressed genes in PXVE transgenic lines.**
(PDF)

Table S6 **Motifs enrichment in the promoters of repressed genes in** ***PXVE:NF-YA2SRDX*** **lines that change in its** ***PXVE:NF-YA2*** **counterpart, putative indirect targets.**
(PDF)

Table S7 **Z-score for TATA-box and CCAAT-box motifs in the promoters of repressed genes in** ***PXVE:NF-YA2SRDX*** **line that change in its** ***PXVE:NF-YA2*** **counterpart, putative indirect targets.**
(PDF)

Table S8 **Sequences of primers and probes used in this study.**
(PDF)

Dataset S1 **Transcripts differentially expressed in** ***PXVE:NF-YA*** **,** ***PXVE:miR169nm*** **and** ***PXVE:NF-YA2SRDX*** **lines.**
(XLS)

Dataset S2 **Expression profiles of differential genes identified in at least once of the evaluated lines,** ***PXVE:NF-YA*** **,** ***PXVE:miR169nm*** **and** ***PXVE:NF-YA2SRDX*** **, clustered using the Pearson correlation and average linkage clustering.**
(XLS)

Dataset S3 **Functional classification of differentially expressed transcripts in estradiol-inducible lines according to MAPMAN categories using the web-based tool Classification Superviewer.**
(XLS)

Dataset S4 **Putative direct targets of NF-YA2.**
(XLS)
