# Supplementary material for: Functional and Transcriptome Analysis Reveals an Acclimatization Strategy for Abiotic Stress Tolerance Mediated by Arabidopsis NF-YA Family Members
Source: PLoS One. 2012 Oct 31;7(10):e48138. doi: 10.1371/journal.pone.0048138 (PMC3485258; doi:10.1371/journal.pone.0048138)
Supplement: Figure S1 — Temporal and Spatial expression pattern of NF-YAs and miR169s in response to Pi availability. (PDF) [file pone.0048138.s001.pdf]

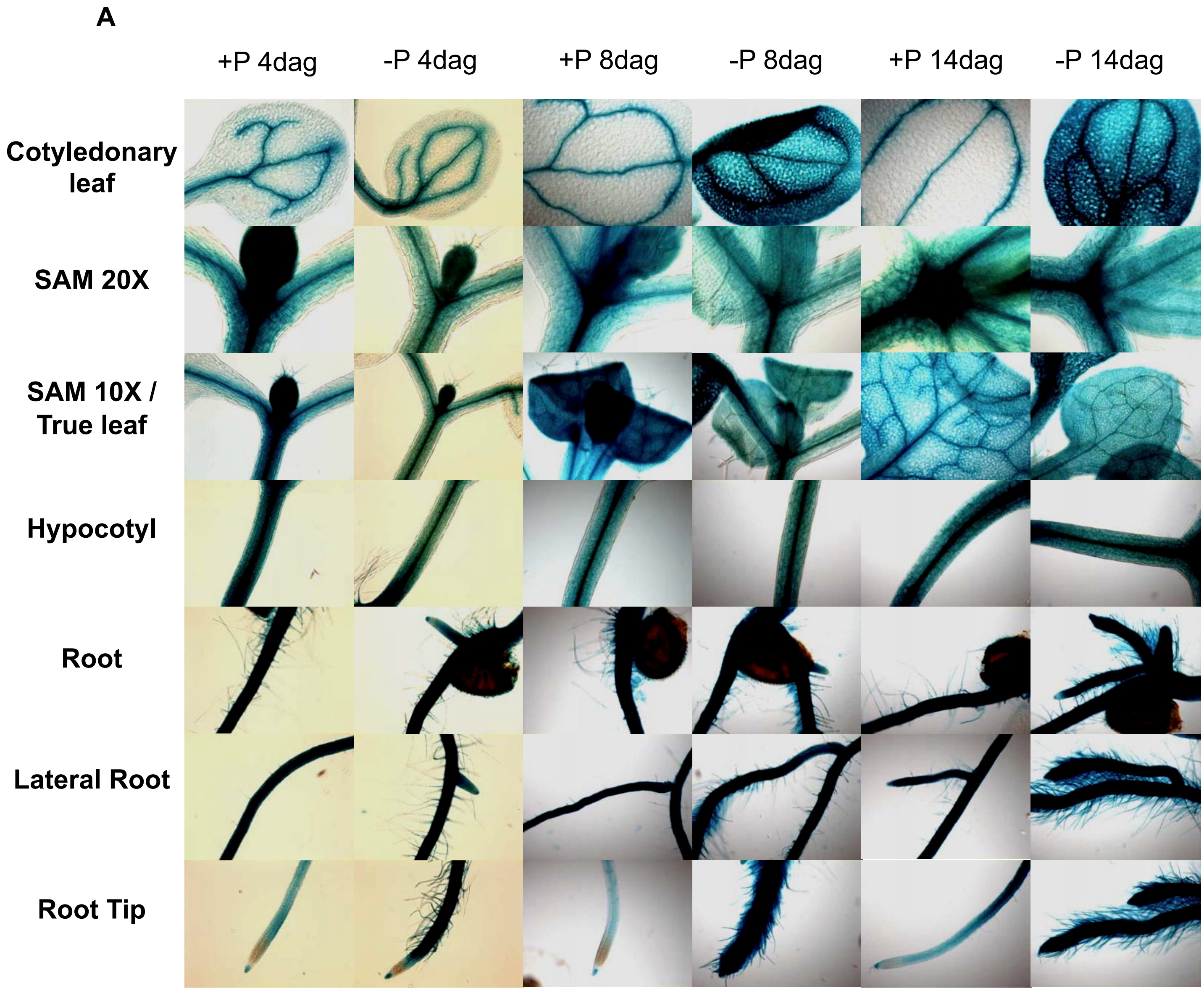

Figure S1. (see next pages)

B

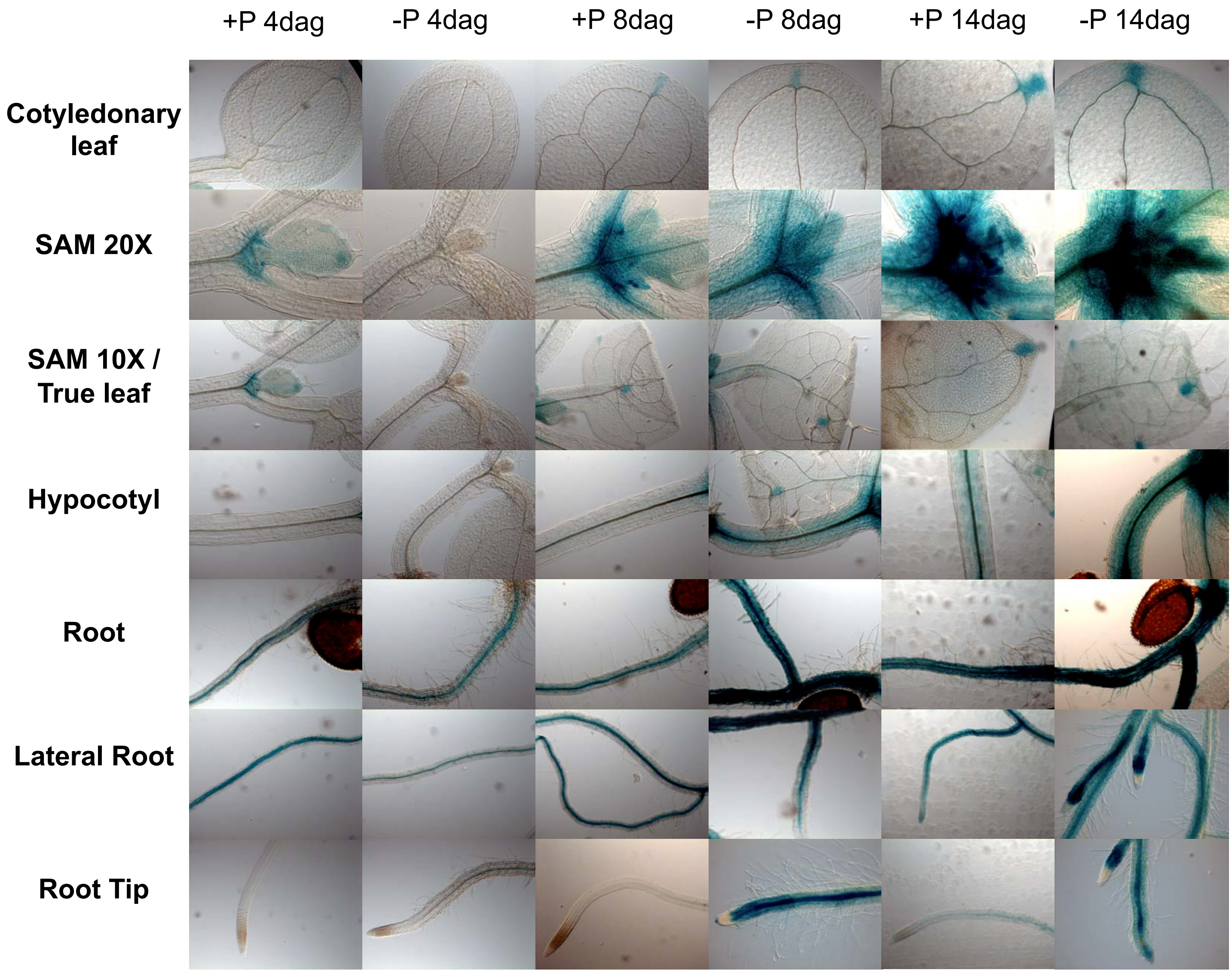

Figure S1. (see next pages)

C

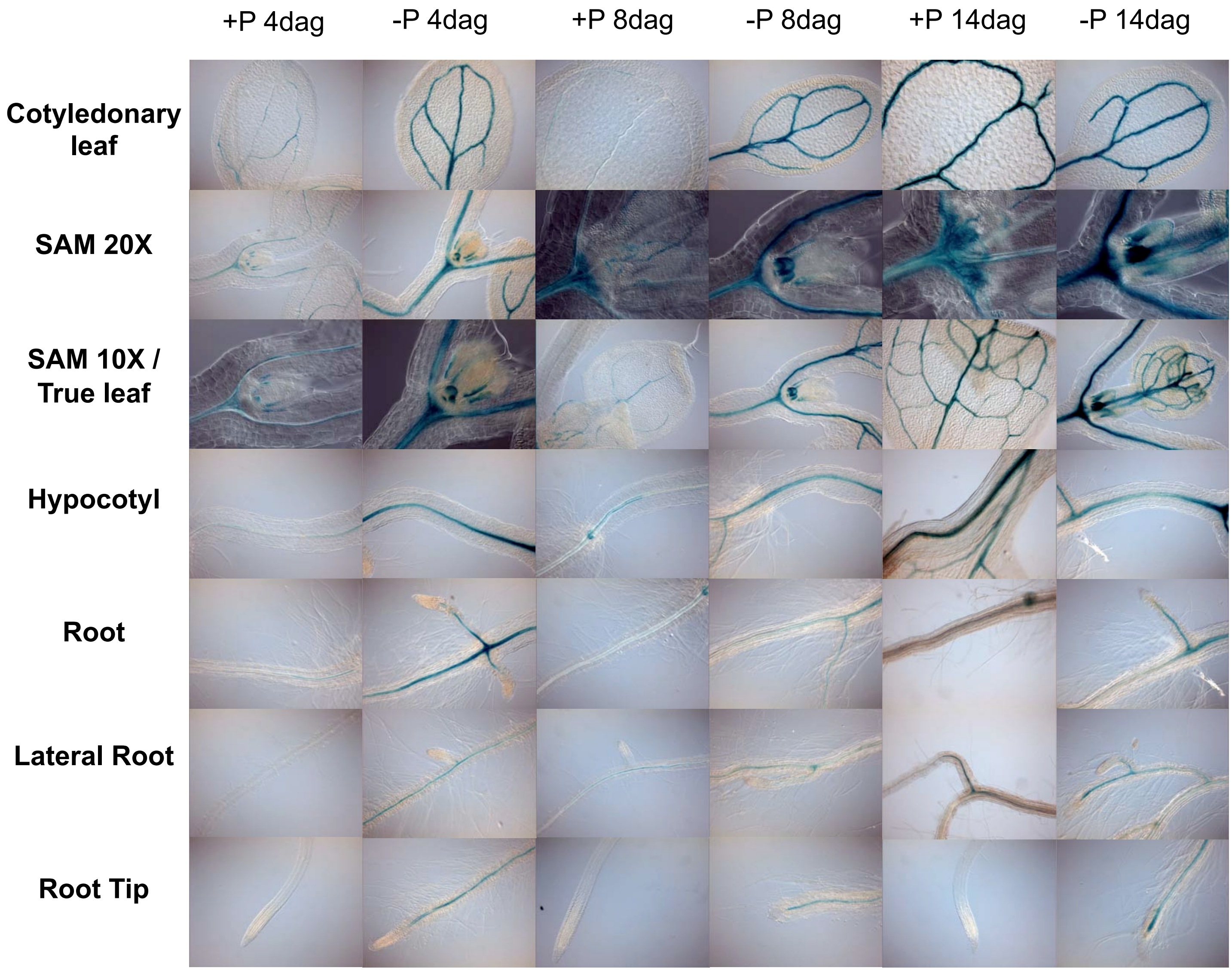

Figure S1. (see next pages)

D

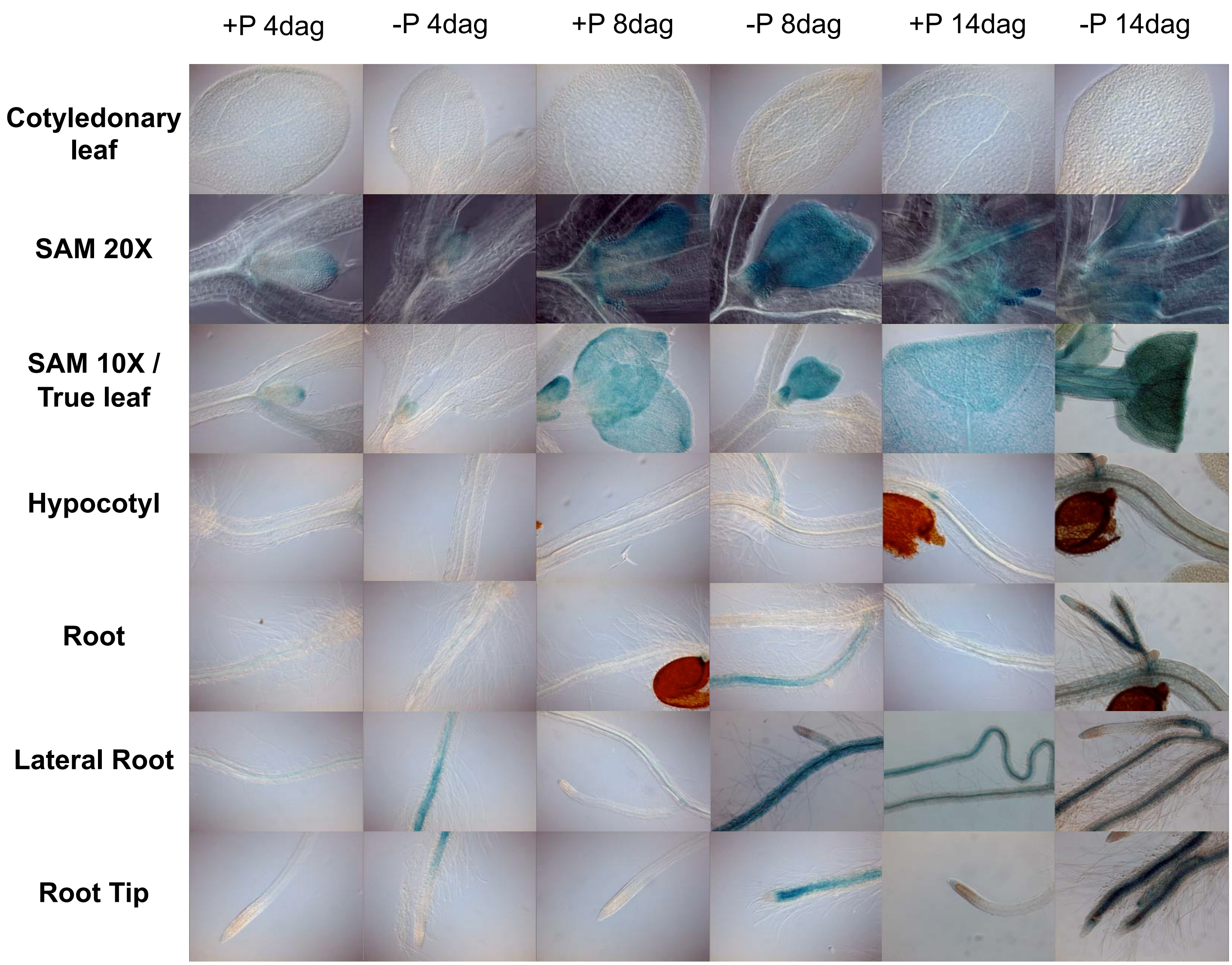

Figure S1. (see next pages)

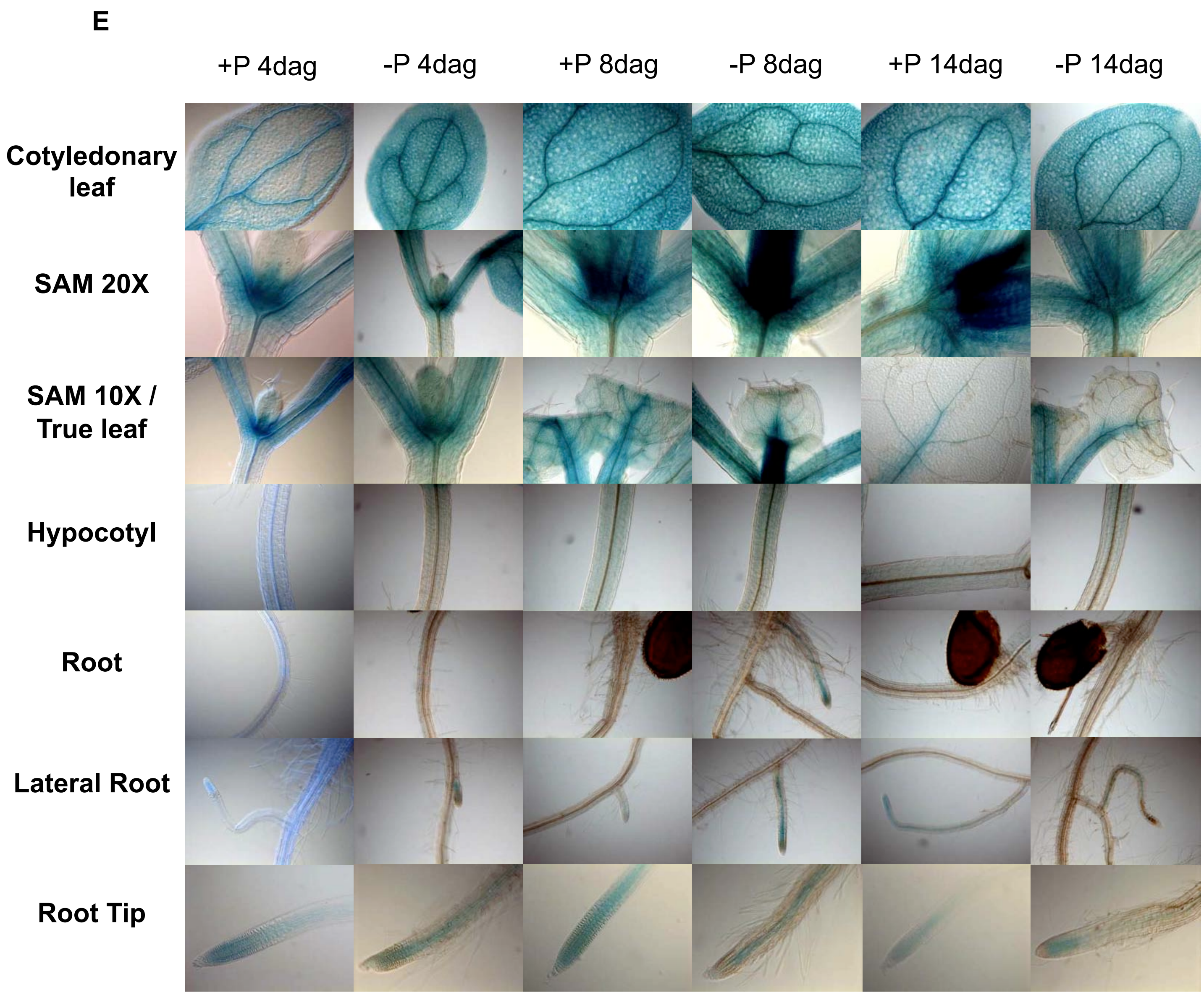

Figure S1. (see next pages)

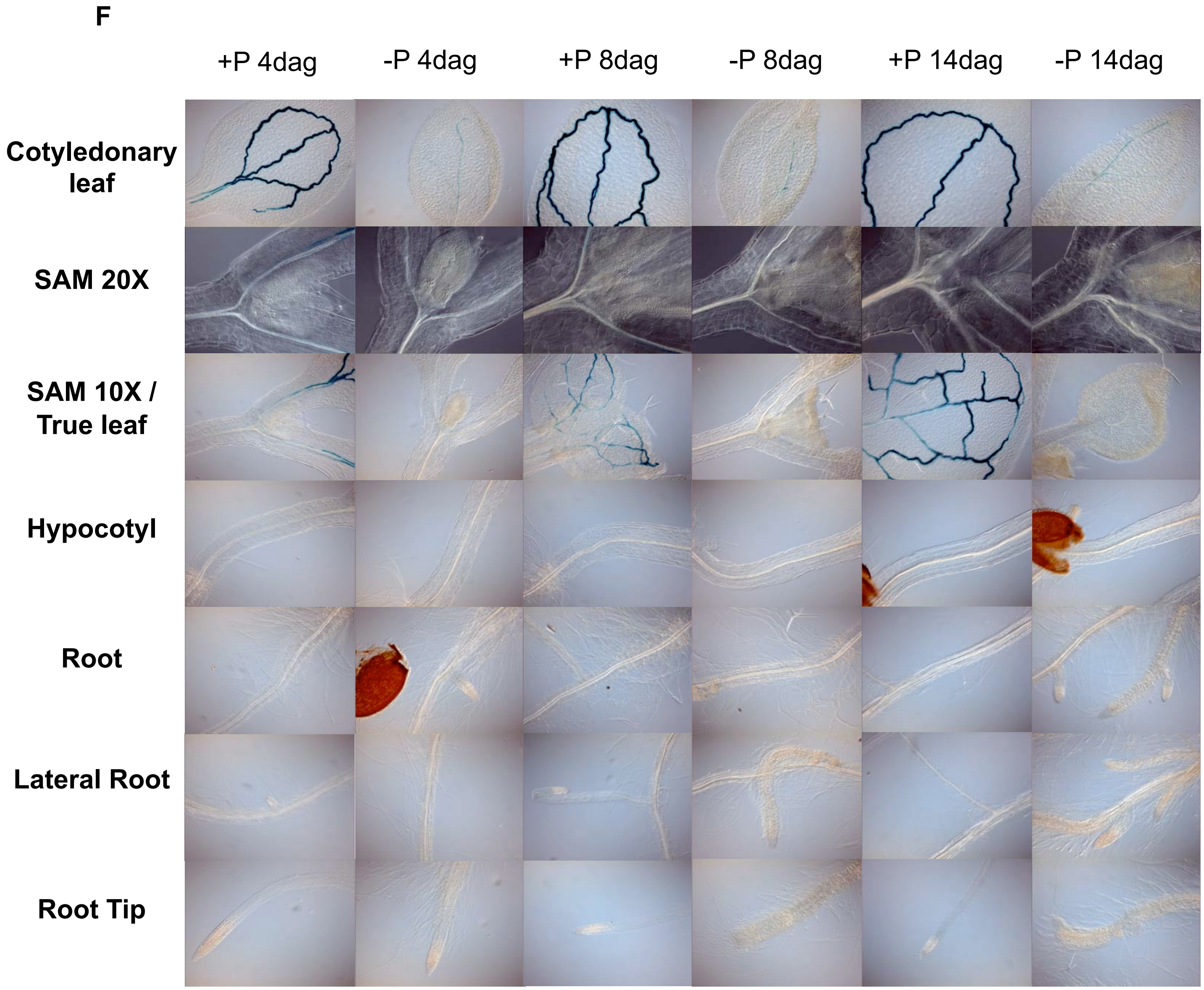

Figure S1. (see next pages)

G

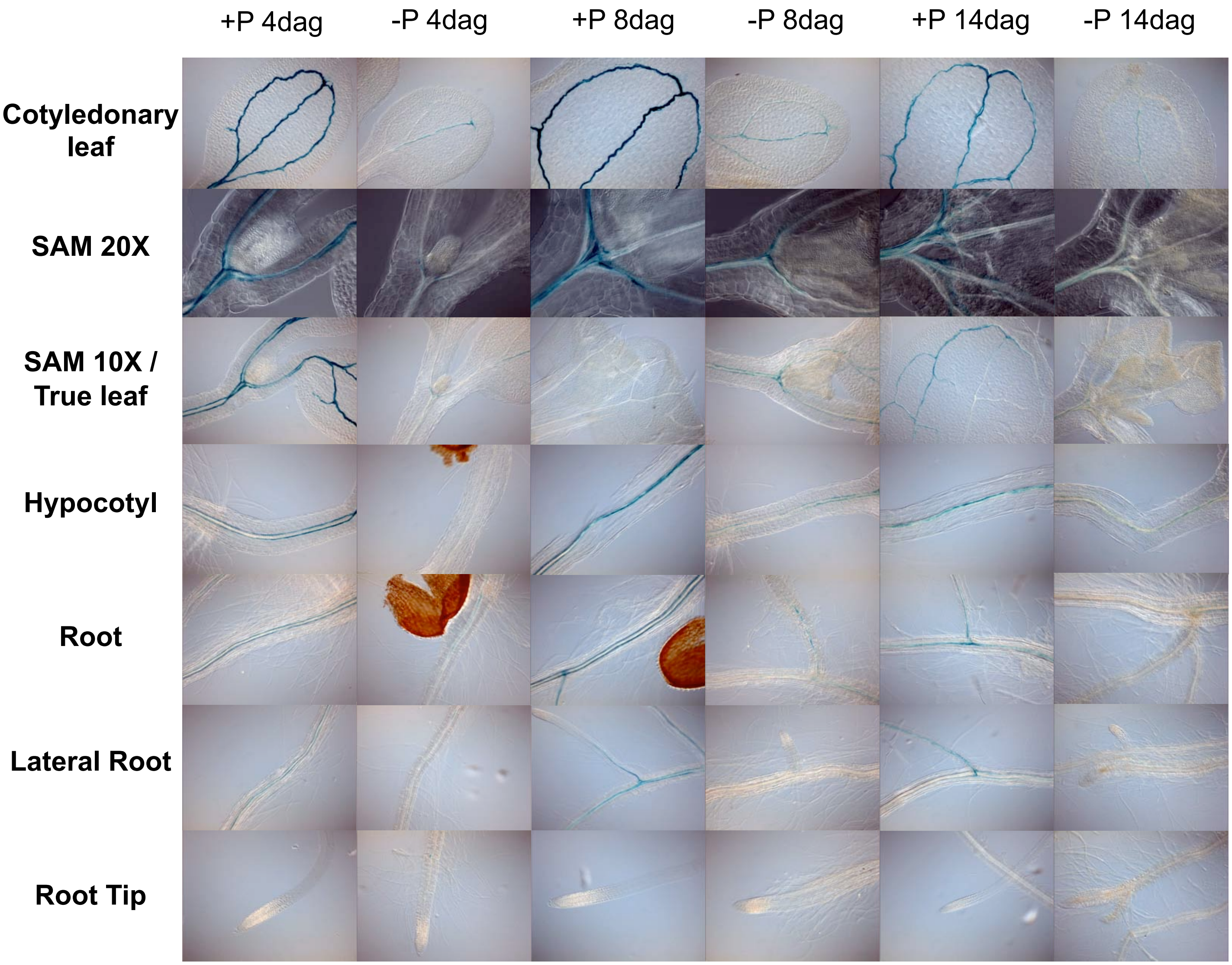

Figure S1. (see next pages)

H

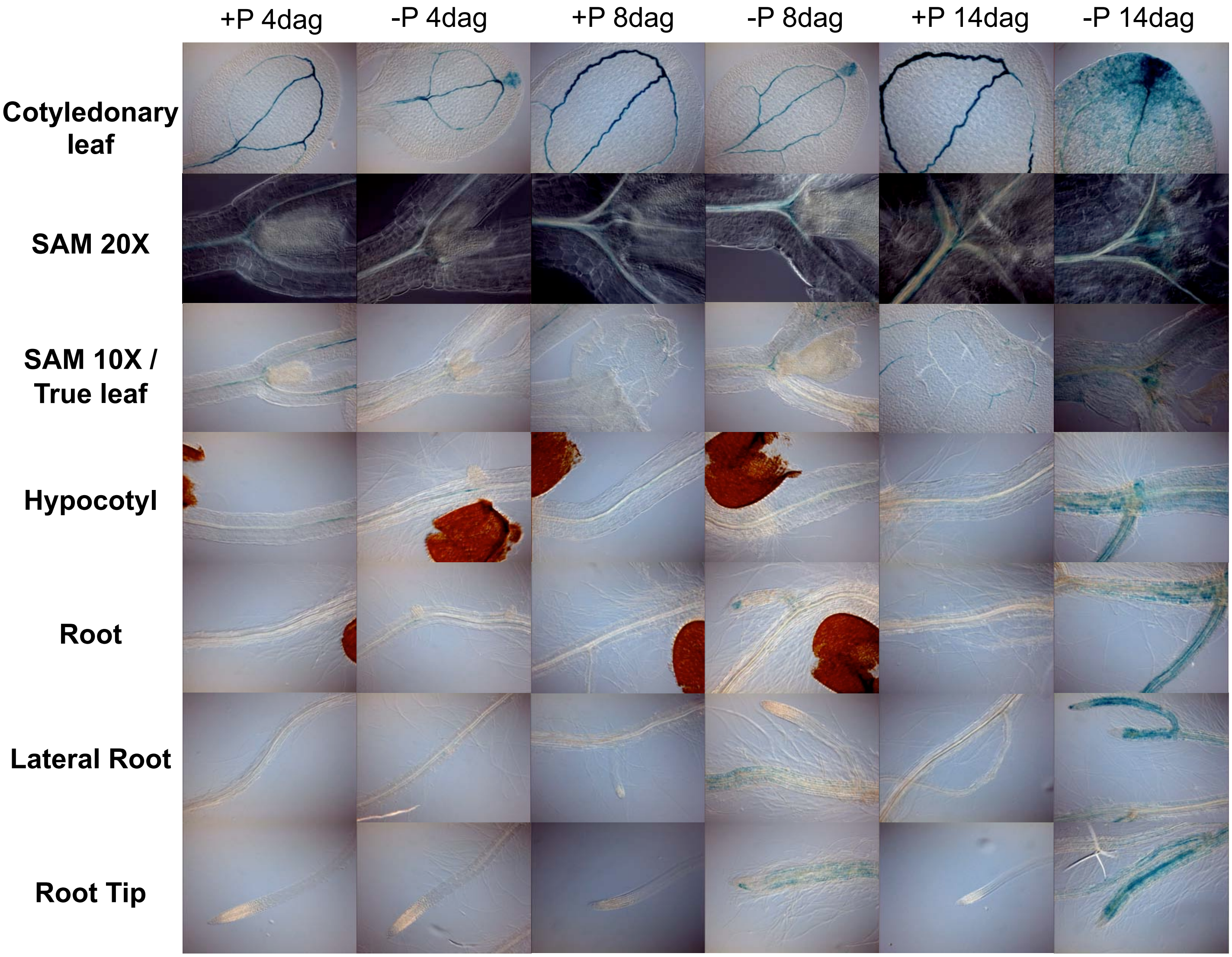

Figure S1. Temporal and spatial expression pattern of *NF-YAs* and miR169s in response to Pi availability.

(A) *PNF-YA2:GUS*
(B) *PNF-YA3:GUS*
(C) *PNF-YA5:GUS*
(D) *PNF-YA7:GUS*
(E) *PmiR169a:GUS*
(F) *PmiR169h:GUS*
(G) *PmiR169k/l:GUS*
(H) *PmiR169m/n:GUS*

Tissue specific expression directed by the *NF-YAs* and miR169s promoters as revealed by GUS histochemical in seedlings grown in Pi contrasting conditions (+P, 1 mM and –P, 5 μM) at the indicated days after germination (dag).
