# Supplementary material for: Functional and Transcriptome Analysis Reveals an Acclimatization Strategy for Abiotic Stress Tolerance Mediated by Arabidopsis NF-YA Family Members
Source: PLoS One. 2012 Oct 31;7(10):e48138. doi: 10.1371/journal.pone.0048138 (PMC3485258; doi:10.1371/journal.pone.0048138)
Supplement: Figure S2 — qRT-PCR analysis of miR169nm overexpressing lines. (PDF) [file pone.0048138.s002.pdf]

A

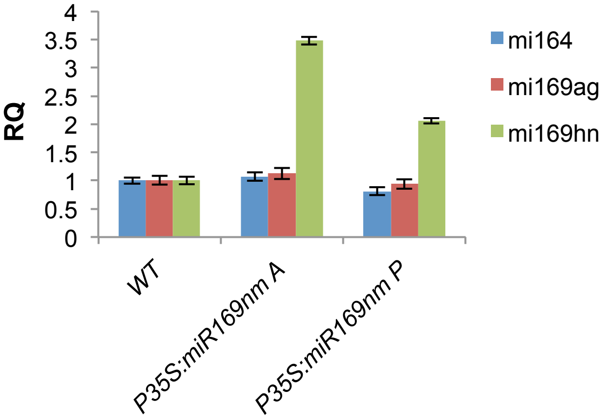

B

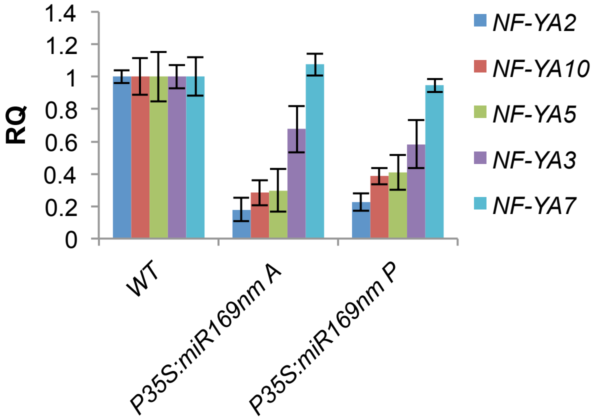

Figure S2. qRT-PCR analysis of miR169nm overexpressing lines.

(A) Mature miR169 transcript levels determined by qRT-PCR, miR164 was used as a control. **(B)** Gene expression profile of *NF-YA Arabidopsis* genes in *P35S:miR169nm* lines.

Eight-day-old plants of two independent miR169nm overexpressing lines were used for RNA extraction in **(A)** and **(B)**. Values represent means and error bars indicate SE of three independent amplification reactions.
