# Supplementary material for: Functional and Transcriptome Analysis Reveals an Acclimatization Strategy for Abiotic Stress Tolerance Mediated by Arabidopsis NF-YA Family Members
Source: PLoS One. 2012 Oct 31;7(10):e48138. doi: 10.1371/journal.pone.0048138 (PMC3485258; doi:10.1371/journal.pone.0048138)
Supplement: Figure S3 — NF-YA expression level in transgenic Arabidopsis lines overexpressing NF-YAs . (PDF) [file pone.0048138.s003.pdf]

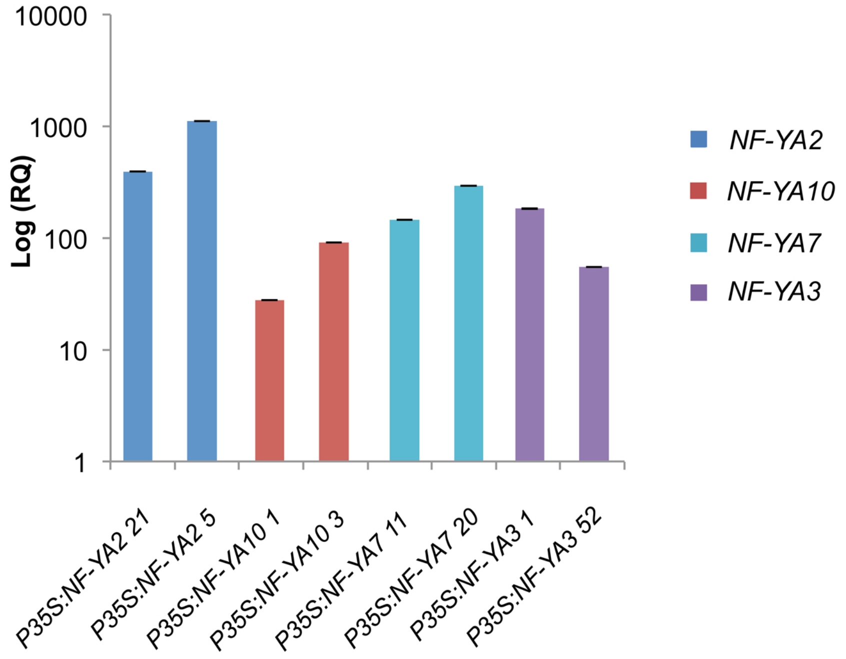

Figure S3. *NF-YA* expression level in transgenic *Arabidopsis* lines overexpressing *NF-YA*.

Transcript levels were determined by qRT-PCR in eight-day-old transgenic and WT seedlings grown on 0.1 X MS agar plates; data are shown in a logarithmic scale. Expression level of *ACT2* was used as internal reference. Values represent means and SE of three independent amplification reactions.
