# Supplementary material for: Functional and Transcriptome Analysis Reveals an Acclimatization Strategy for Abiotic Stress Tolerance Mediated by Arabidopsis NF-YA Family Members
Source: PLoS One. 2012 Oct 31;7(10):e48138. doi: 10.1371/journal.pone.0048138 (PMC3485258; doi:10.1371/journal.pone.0048138)
Supplement: Figure S4 — Ectopic expression of NF-YAs and miR169nm affects seed weight and silique size. (PDF) [file pone.0048138.s004.pdf]

A

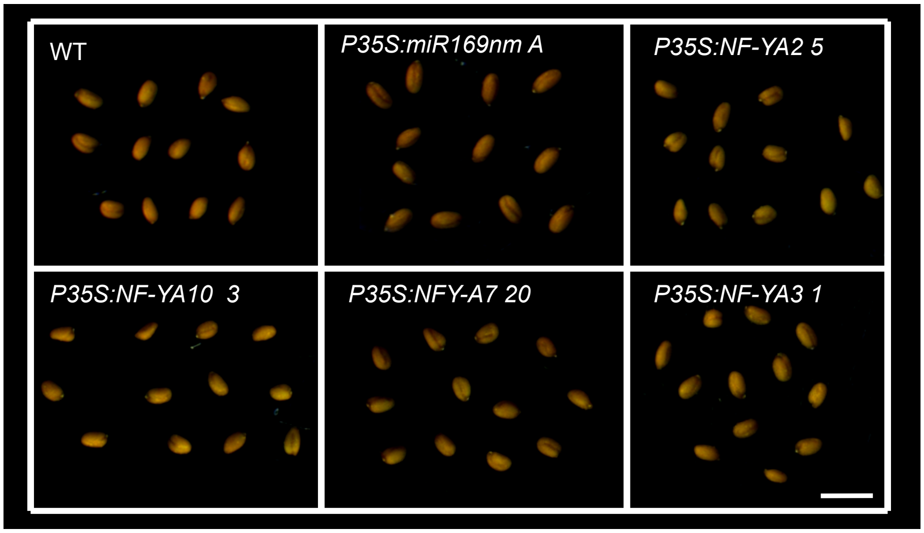

B

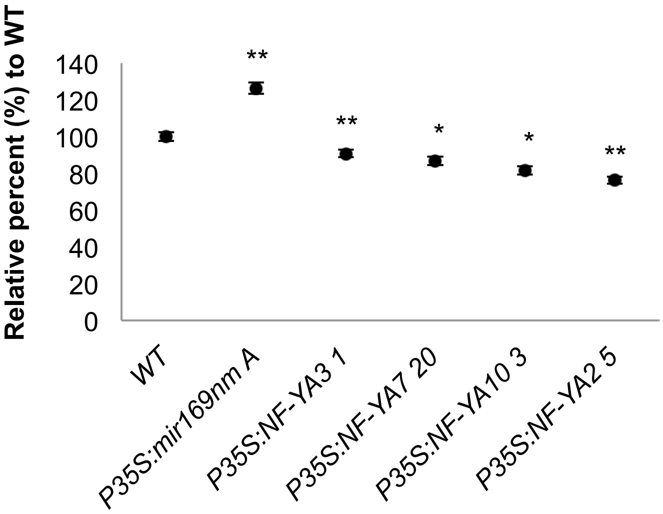

C

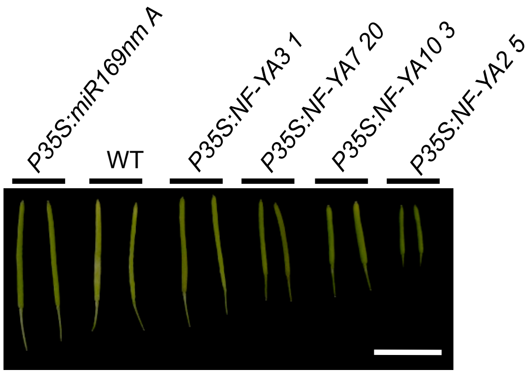

Figure S4. Ectopic expression of *NF-YAs* and miR169nm genes affects seed weight and silique size.

(A) Photographs of seed from *P35S:NF-YA, P35S:mir169nm* and WT lines. **(B)** Weight of 100 seeds of each line from different batches was determined and compared to WT to obtain the relative percent. Data represent means and SD of three replicates statistically treated using a student *t*-test (*P<0.01, **P<0.001). **(C)** Siliques belonging to each transgenic line were photographed at the same developmental stage. Bars = 1mm in **(A)** and 10 mm in **(C)**.
