# Supplementary material for: Functional and Transcriptome Analysis Reveals an Acclimatization Strategy for Abiotic Stress Tolerance Mediated by Arabidopsis NF-YA Family Members
Source: PLoS One. 2012 Oct 31;7(10):e48138. doi: 10.1371/journal.pone.0048138 (PMC3485258; doi:10.1371/journal.pone.0048138)
Supplement: Figure S5 — Biomass accumulation is affected in P35S:NF-YA and P35S:miR169nm lines. (PDF) [file pone.0048138.s005.pdf]

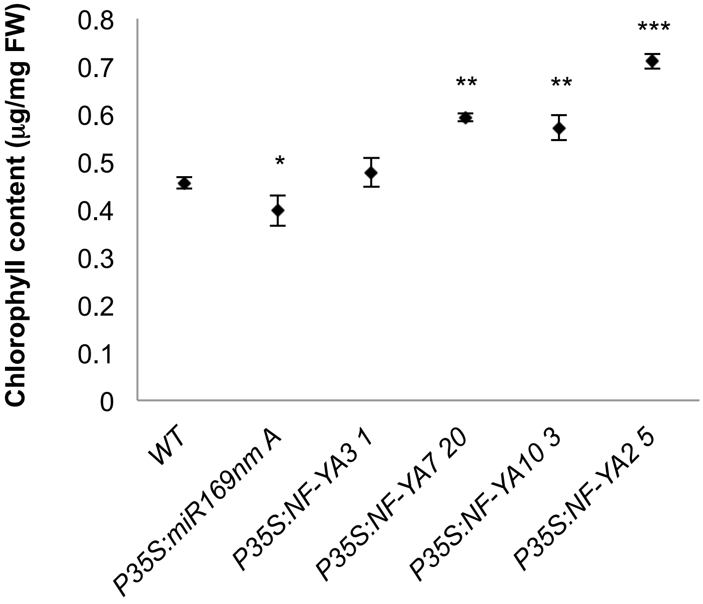

Figure S5. Chlorophyll content of wild-type, *P35S:NF-YA* and *P35S:miR169nm* lines.

Total chlorophyll content of plants grown on 0.1 X MS agar plates for 2 weeks was measured and normalized per milligram fresh weight of sample. Values represent means and SD of three biological replicates statistically treated using a student *t*-test (*P<0.05, **P<0.001, ***P<0.0001).
