# Supplementary material for: Functional and Transcriptome Analysis Reveals an Acclimatization Strategy for Abiotic Stress Tolerance Mediated by Arabidopsis NF-YA Family Members
Source: PLoS One. 2012 Oct 31;7(10):e48138. doi: 10.1371/journal.pone.0048138 (PMC3485258; doi:10.1371/journal.pone.0048138)
Supplement: Figure S6 — Chlorophyll content of wild-type, P35S:NF-YA and P35S:miR169nm lines. (PDF) [file pone.0048138.s006.pdf]

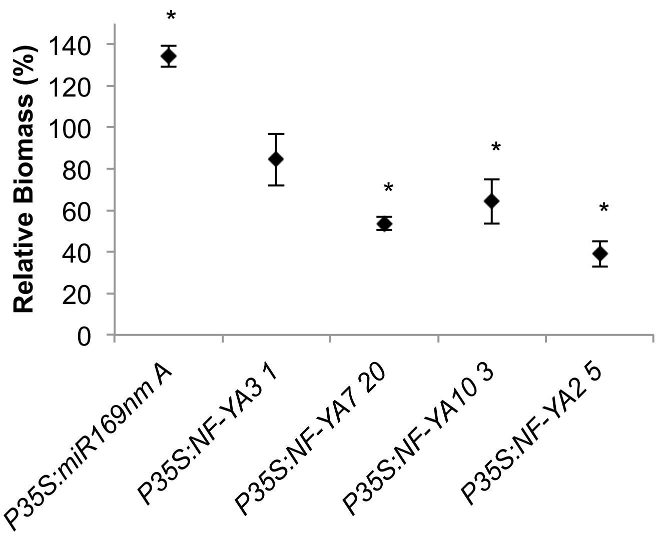

Figure S6. Biomass accumulation is affected in *P35S:NF-YA* and *P35S:miR169nm* lines.

Total biomass of twelve-day-old seedlings was determined and used to calculate the relative biomass to the WT (expressed as a percent of the value for WT line growing on control medium, set to 100 %). Values are means and SD of three biological replicates statistically treated using a student *t*-test (*P<0.05).
