# Supplementary material for: Functional and Transcriptome Analysis Reveals an Acclimatization Strategy for Abiotic Stress Tolerance Mediated by Arabidopsis NF-YA Family Members
Source: PLoS One. 2012 Oct 31;7(10):e48138. doi: 10.1371/journal.pone.0048138 (PMC3485258; doi:10.1371/journal.pone.0048138)
Supplement: Figure S7 — MAPMAN-based functional classification of differentially expressed transcripts in PXVE:NF-YA and PXVE:miR169nm lines. (PDF) [file pone.0048138.s007.pdf]

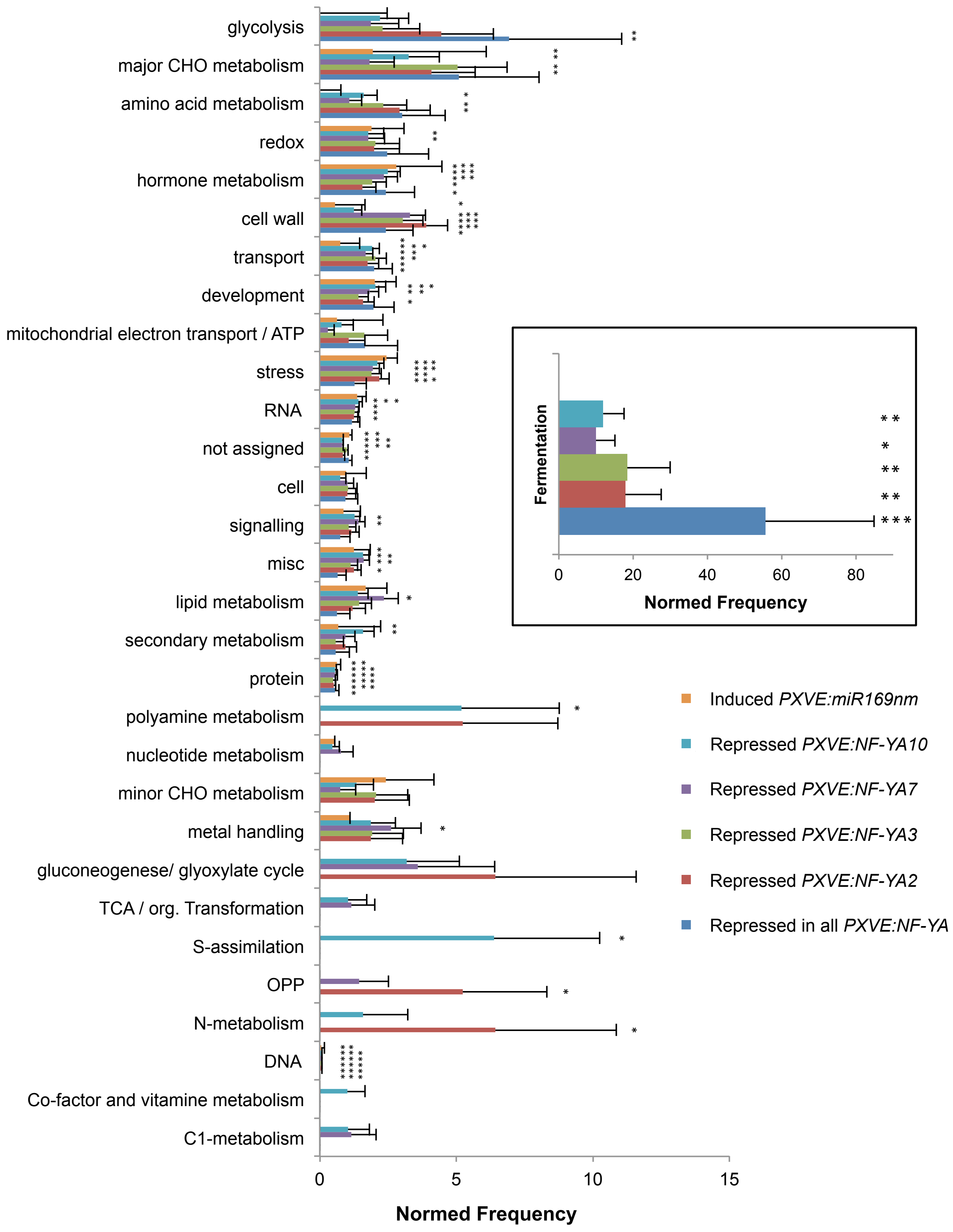

Figure S7. MAPMAN-based functional classification of differentially expressed transcripts in *PXVE:NF-YA* and *PXVE:miR169nm* lines.

MAPMAN classification of the transcripts according to MAPMAN categories using the web-based tool Classification Superviewer (http://bar.utoronto.ca) with normalized class score option. One, two and three asterisks indicate a p-value < 0.05, 0.001 and 0.0001, respectively.
