# Supplementary material for: Functional and Transcriptome Analysis Reveals an Acclimatization Strategy for Abiotic Stress Tolerance Mediated by Arabidopsis NF-YA Family Members
Source: PLoS One. 2012 Oct 31;7(10):e48138. doi: 10.1371/journal.pone.0048138 (PMC3485258; doi:10.1371/journal.pone.0048138)
Supplement: Figure S8 — Dark-induced cell elongation is affected in NF-YA overexpressing plants. (PDF) [file pone.0048138.s008.pdf]

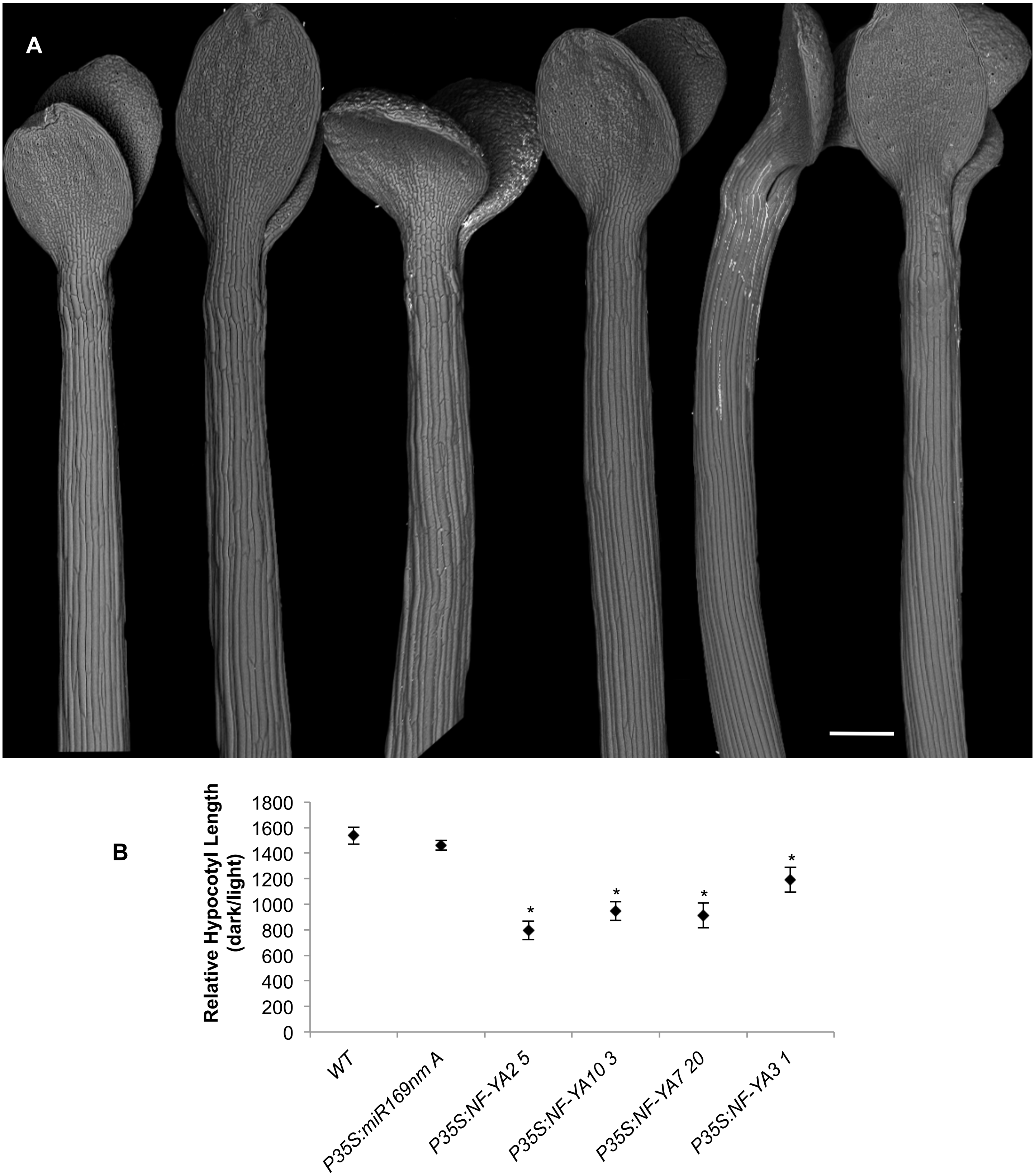

Figure S8. Dark-induced cell elongation is affected in *NF-YA* overexpressing plants.

(A) Scanning electron microscope images of 5-day-old dark-grown (from left to right) *P35S:NF-YA2*, *10*, *7*, *3*; WT and *P35S:miR169nm* seedlings. Bar = 200 μm. **(B)** Dark/Light relative hypocotyl length of 5-day-old WT, *P35:NF-YA* and *P35S:miR169nm* seedlings. Light and dark-grown hypocotyl length was measured and the data used to calculate the relative value. Values are means and SD of three biological replicates statistically treated using a student *t*-test (*P<0.01).
