# Supplementary material for: Functional and Transcriptome Analysis Reveals an Acclimatization Strategy for Abiotic Stress Tolerance Mediated by Arabidopsis NF-YA Family Members
Source: PLoS One. 2012 Oct 31;7(10):e48138. doi: 10.1371/journal.pone.0048138 (PMC3485258; doi:10.1371/journal.pone.0048138)
Supplement: Figure S9 — NF-YA overexpression causes a starch excess phenotype. (PDF) [file pone.0048138.s009.pdf]

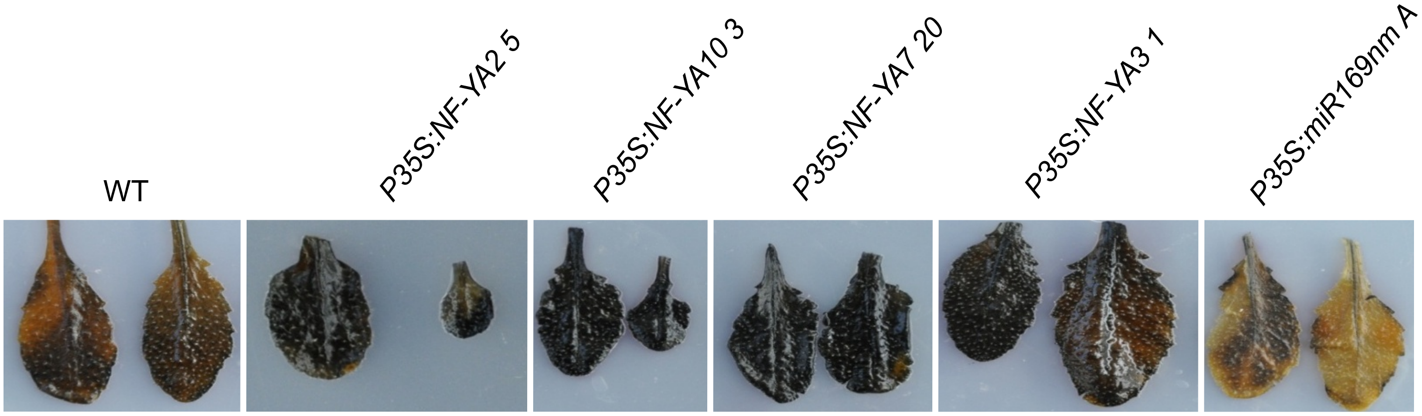

Figure S9. *NF-YA* overexpression causes a starch excess phenotype.

Starch content in 3-week-old leaves belonging to WT, *P35S:NF-YA* and *P35S:miR169nm* lines. Plants were decolorized in hot 80% (v/v) ethanol and stained for starch with iodine solution before photographs were taken.
