# Supplementary material for: Functional and Transcriptome Analysis Reveals an Acclimatization Strategy for Abiotic Stress Tolerance Mediated by Arabidopsis NF-YA Family Members
Source: PLoS One. 2012 Oct 31;7(10):e48138. doi: 10.1371/journal.pone.0048138 (PMC3485258; doi:10.1371/journal.pone.0048138)
Supplement: Figure S10 — NF-YA overexpressing lines display increased drought tolerance. (PDF) [file pone.0048138.s010.pdf]

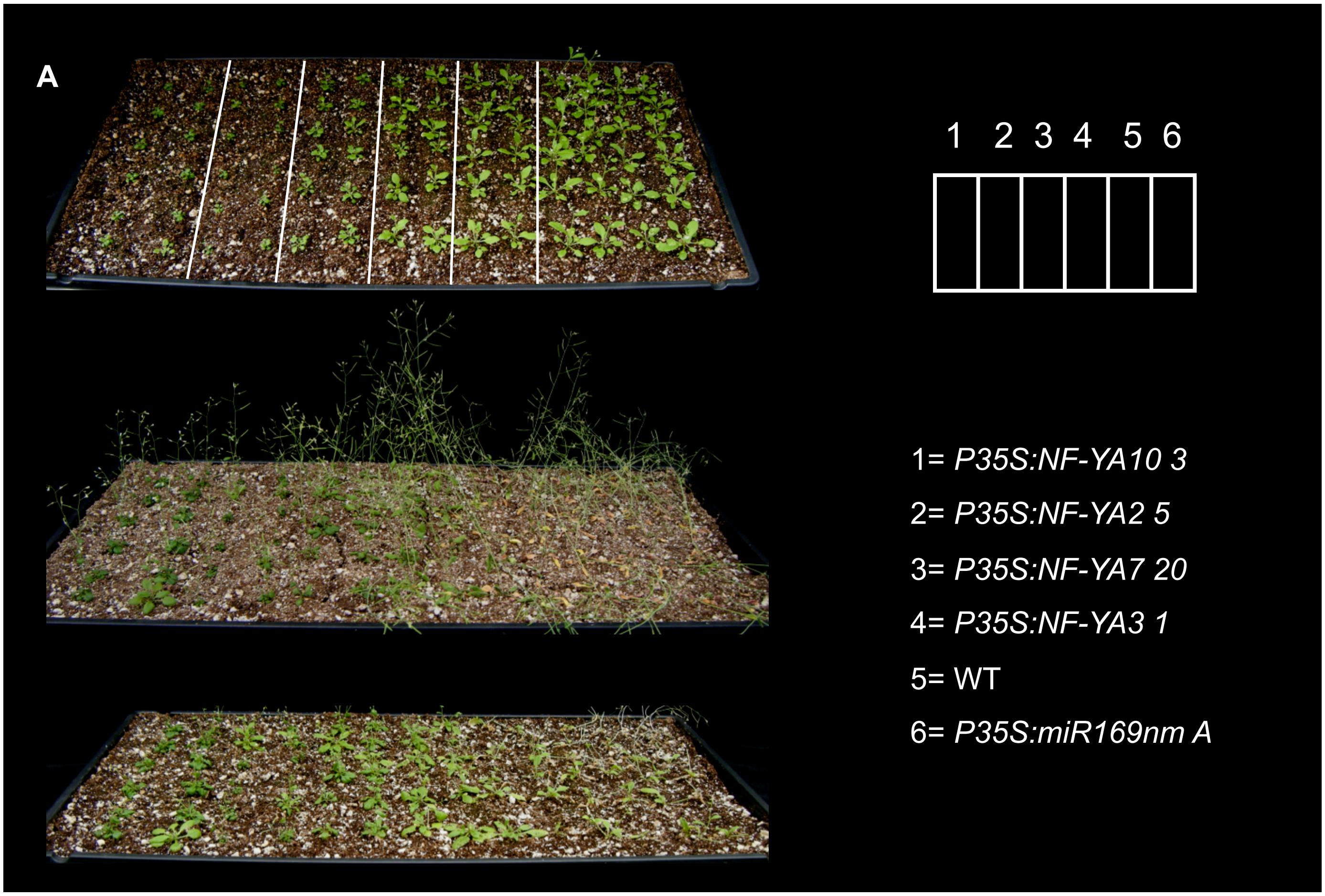

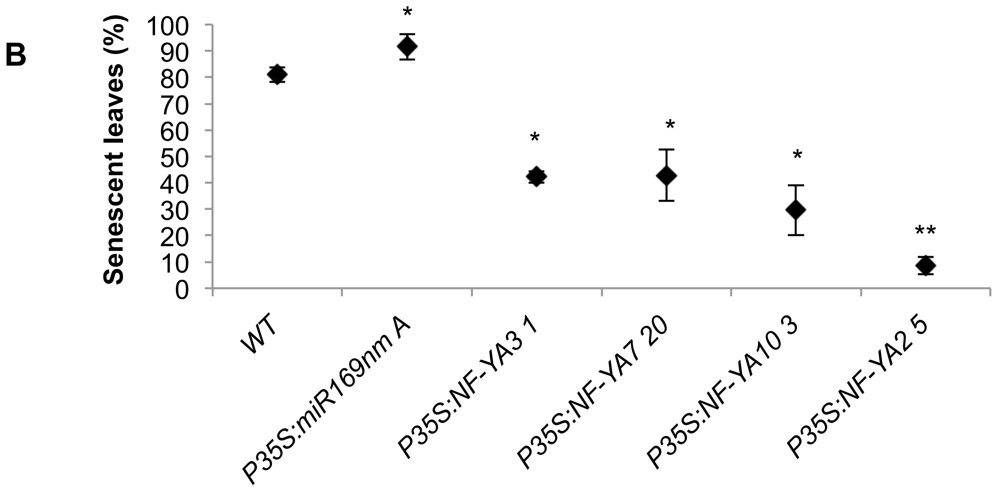

Figure S10. *NF-YA* overexpressing lines display increased drought tolerance.

(A) Images of WT, *P35S:NF-YA* and *P35S:miR169nm* lines throughout the drought assay. The upper row shows plants grown in soil with sufficient water for 3 weeks, the middle row shows the same plants subjected to a drought stress in which water was withheld for 12 days and the lower row shows the plants five days after a recovery irrigation (flowering stems were cut to show the rosette leaves). A schematic diagram of the plants distribution is shown. **(B)** Percentage of senescent leaves in plants subjected to drought for 12 days and 5 days after recovery irrigation. Values are means and SD of three biological replicates (n = 12, at least) statistically treated using a student *t*-test (*P<0.05, **P<0.001).
