# Supplementary material for: Functional and Transcriptome Analysis Reveals an Acclimatization Strategy for Abiotic Stress Tolerance Mediated by Arabidopsis NF-YA Family Members
Source: PLoS One. 2012 Oct 31;7(10):e48138. doi: 10.1371/journal.pone.0048138 (PMC3485258; doi:10.1371/journal.pone.0048138)
Supplement: Figure S11 — NF-YA overexpression enhances heat and freezing tolerance. (PDF) [file pone.0048138.s011.pdf]

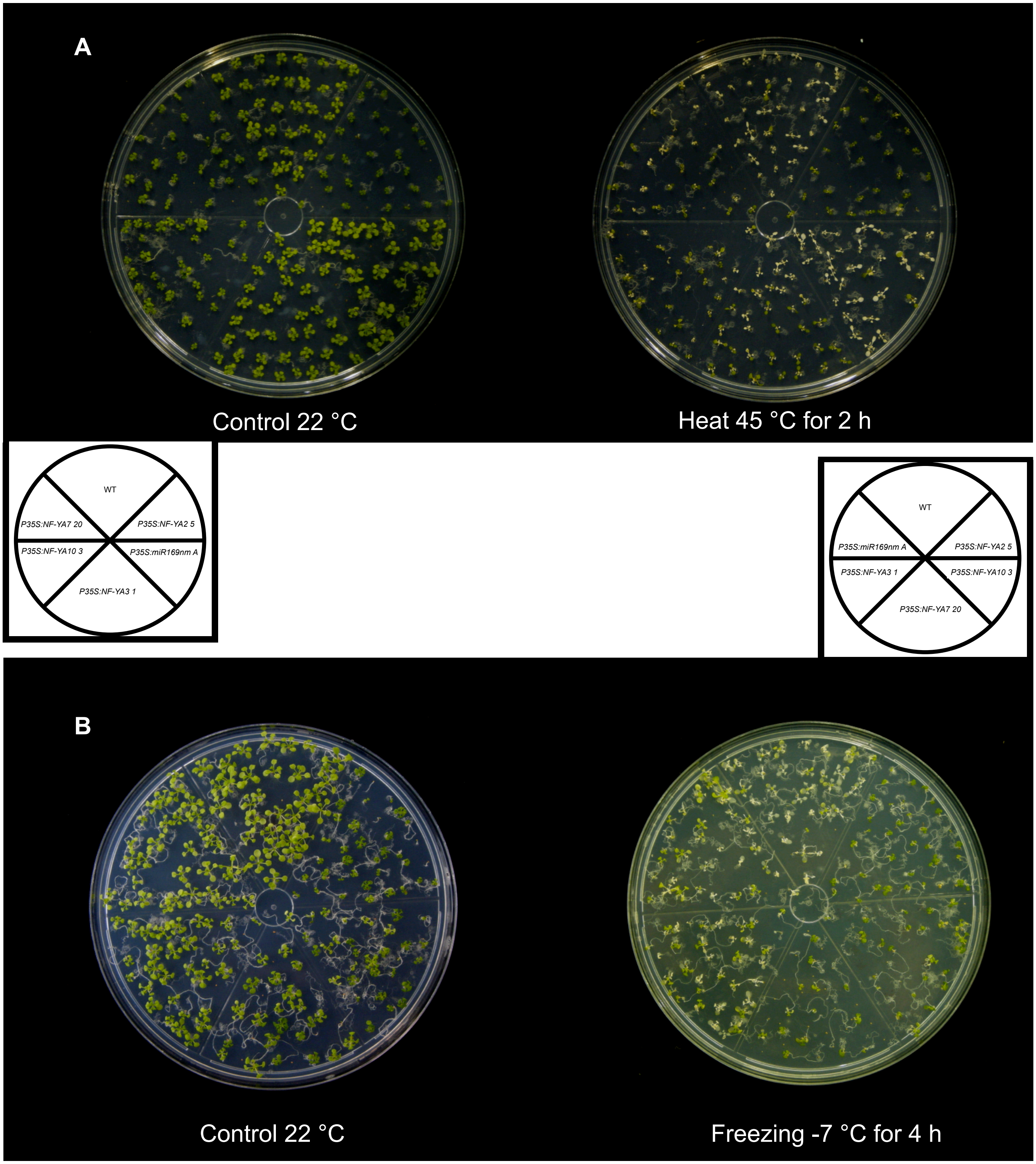

Figure S11. *NF-YA* overexpression enhances heat and freezing tolerance.

(A) (B) Images of twelve-day-old WT, *P35S:NF-YA* and *P35S:miR169nm* seedlings (see schematic distribution in each panel) grown on 0.1 X MS subjected to control (left), heat **(A)** and freezing **(B)**, (right) conditions. Photographs were taken after three days of recovery time.
