# Supplementary material for: Functional and Transcriptome Analysis Reveals an Acclimatization Strategy for Abiotic Stress Tolerance Mediated by Arabidopsis NF-YA Family Members
Source: PLoS One. 2012 Oct 31;7(10):e48138. doi: 10.1371/journal.pone.0048138 (PMC3485258; doi:10.1371/journal.pone.0048138)
Supplement: Figure S12 — NF-YA overexpression delays dark-induced senescence. (PDF) [file pone.0048138.s012.pdf]

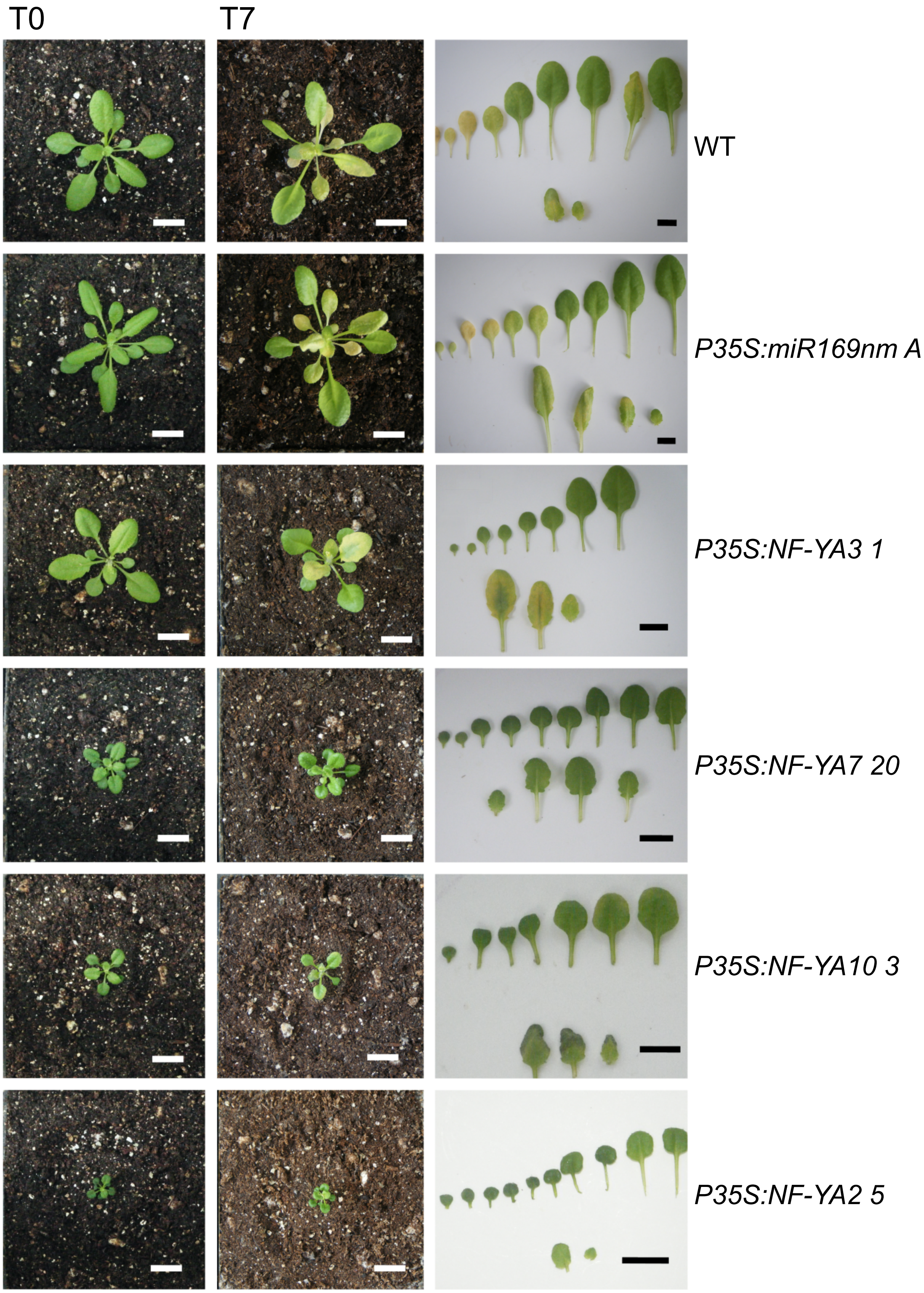

Figure S12. *NF-YA* overexpression delays dark-induced senescence.

Images of four-week-old wild type (WT), *P35S:NF-YA* and *P35S:miR169nm* plants before (T0) and 7 days after dark treatment (T7). The left column shows the phenotypes of whole plants at the beginning of the assay (T0); the middle column of the plants subjected to the treatment (T7) and the right column the rosette leaves of T7 plants. Bars = 10 mm, on the left and middle panels, and 5 mm on the right panels.
