# Supplementary material for: Functional and Transcriptome Analysis Reveals an Acclimatization Strategy for Abiotic Stress Tolerance Mediated by Arabidopsis NF-YA Family Members
Source: PLoS One. 2012 Oct 31;7(10):e48138. doi: 10.1371/journal.pone.0048138 (PMC3485258; doi:10.1371/journal.pone.0048138)
Supplement: Figure S13 — NF-YA overexpression prolongs plant longevity. (PDF) [file pone.0048138.s013.pdf]

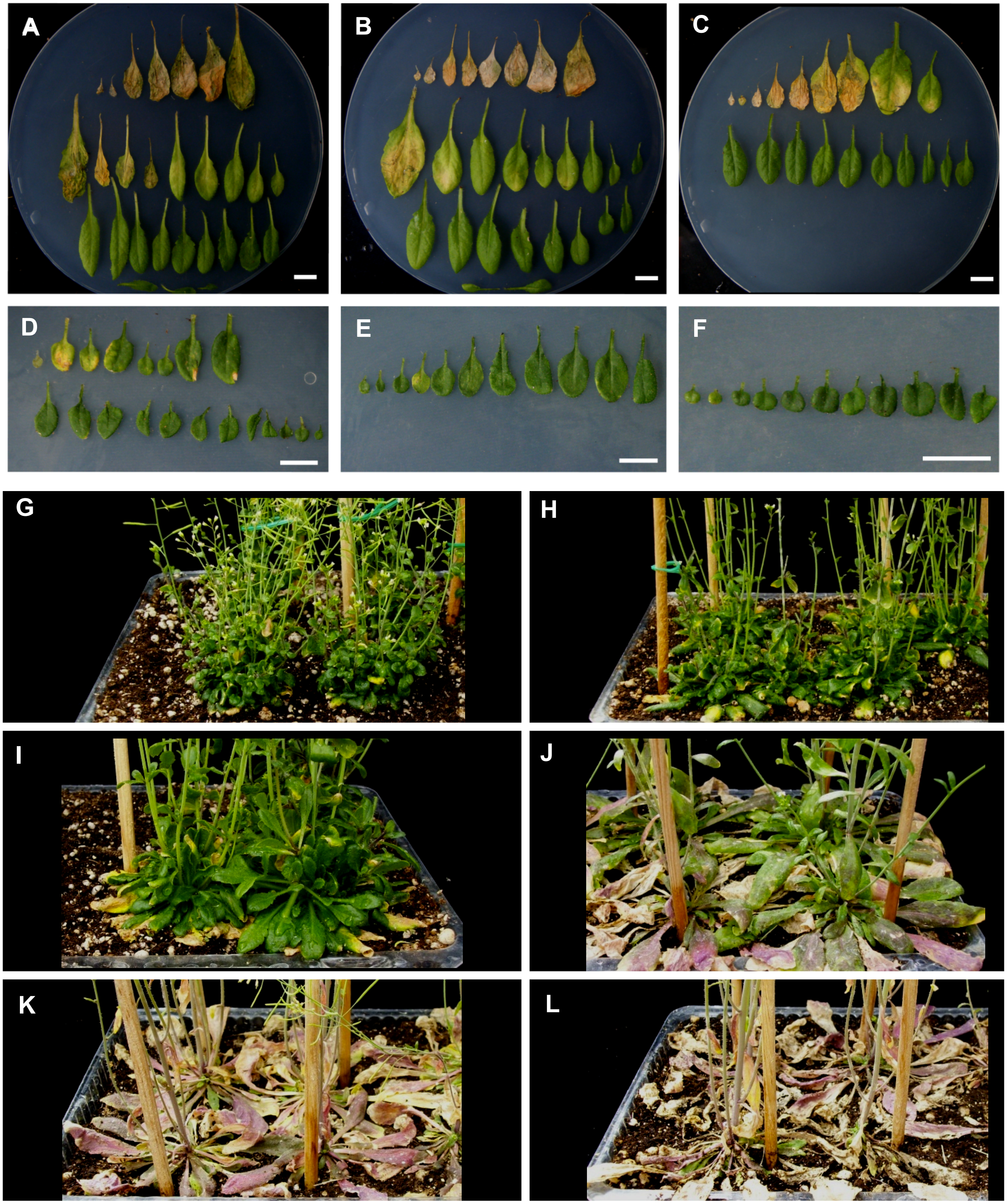

Figure S13. *NF-YA* overexpression prolongs plant longevity.

(A) to **(F)** Senescence of rosette leaves of eight-week-old *P35S:miR169nm*, WT, *P35S:NF-YA3, 7, 10, 2,* plants, respectively grown in soil. Bars = 10 mm. **(G)** to **(L)** Photographs of twelve-week-old plants grown in soil belonging to *P35S:NF-YA2, 10, 7, 3,* WT and *P35S:miR169nm* lines, respectively.
