# Supplementary material for: Functional and Transcriptome Analysis Reveals an Acclimatization Strategy for Abiotic Stress Tolerance Mediated by Arabidopsis NF-YA Family Members
Source: PLoS One. 2012 Oct 31;7(10):e48138. doi: 10.1371/journal.pone.0048138 (PMC3485258; doi:10.1371/journal.pone.0048138)
Supplement: Figure S14 — qRT-PCR analysis of WT, PXVE:NF-YA2 and P35S:NF-YA2 lines subjected to freezing. (PDF) [file pone.0048138.s014.pdf]

A

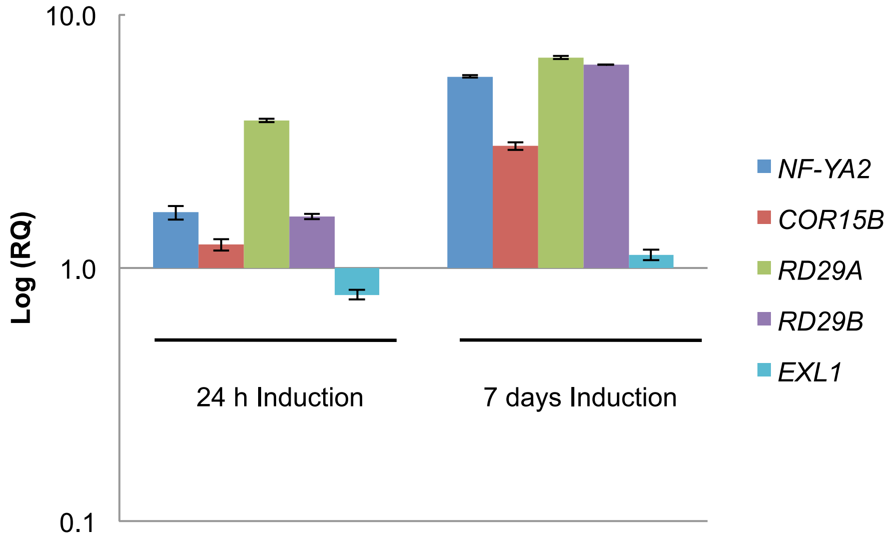

B

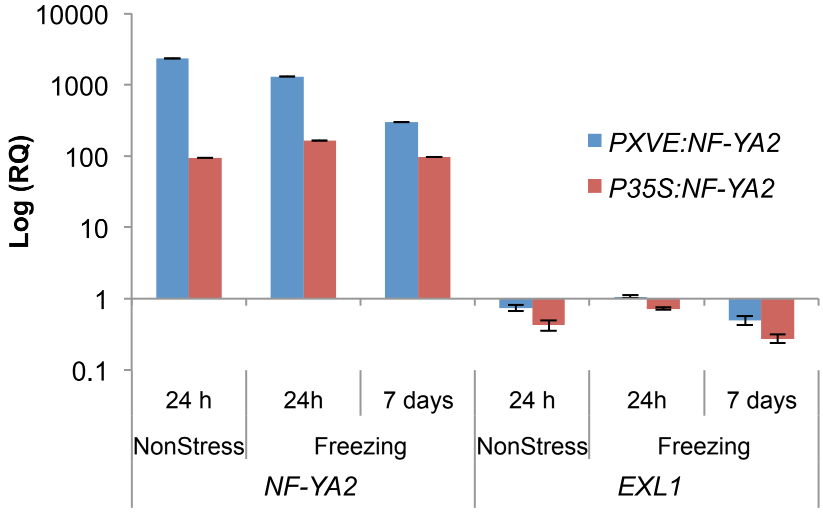

Figure S14. qRT-PCR analysis of WT, *PXVE:NF-YA2* and *P35S:NF-YA2* lines subjected to freezing.

(A) Expression profile of abiotic stress and a growth promoting markers genes in WT seedlings exposed to freezing conditions (-7 °C / 40 min). **(B)** Transcript quantification of *NF-YA2* and *EXL* genes following estradiol treatment for 24 h and 7 days in *PXVE:NF-YA2* and *P35S:NF-YA2* lines exposed to freezing

In **(A)** and **(B)** Seedlings were grown on solid media 0.1 X MS for eight days and then were transferred to 0.1 X MS+ β-estradiol (7 μM) for 24 h or 7 days prior freezing exposition. Total RNA was extracted before and after 40 min of treatment and transcript levels for *NF-YA2, COR15B, RD29A, RD29B and EXL1 were* determined by qRT-PCR; data are shown in a logarithmic scale. Expression level of *ACT2* was used as internal reference. Values represent the means and error bars indicate the standard error (SE) of 3 independent amplification replicates.
