# Supplementary material for: Functional and Transcriptome Analysis Reveals an Acclimatization Strategy for Abiotic Stress Tolerance Mediated by Arabidopsis NF-YA Family Members
Source: PLoS One. 2012 Oct 31;7(10):e48138. doi: 10.1371/journal.pone.0048138 (PMC3485258; doi:10.1371/journal.pone.0048138)
Supplement: Figure S15 — Dose-dependent effect of estradiol on seedling phenotype of homozygote and heterozygote PXVE:NF-YA2SRDX transgenic lines. (PDF) [file pone.0048138.s015.pdf]

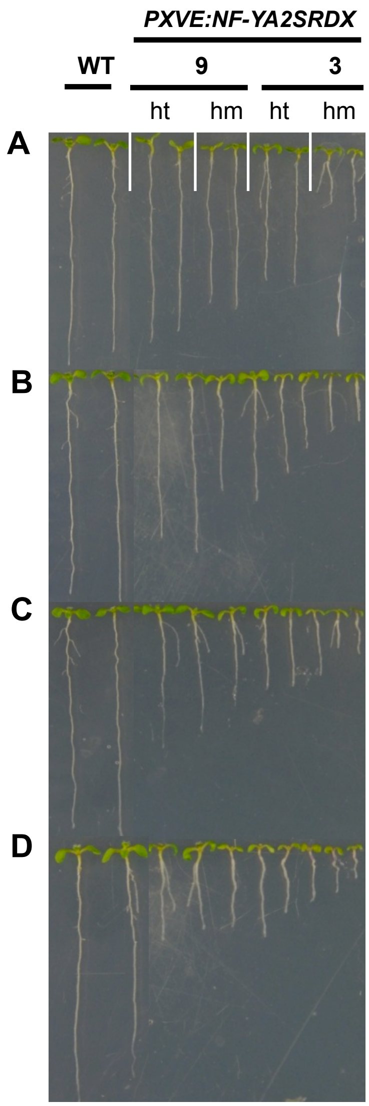

Figure S15. Dose-dependent effect of estradiol on seedling phenotype of homozygote and heterozygote *PXVE:NF-YA2SRDX* transgenic plants.

Eight-day-old transgenic and WT seedlings grown on media supplemented with different concentrations of estradiol ranging from 20 nM to 20 μM belonging to two independent segregating (ht, heterozygote; hm, homozygote) transgenic lines (line numbers are given above the photograph) were analyzed. **(A)** 20 nM; **(B)** 200 nM; **(C)** 2 μM; **(D)** 20 μM.
