# Supplementary material for: Functional and Transcriptome Analysis Reveals an Acclimatization Strategy for Abiotic Stress Tolerance Mediated by Arabidopsis NF-YA Family Members
Source: PLoS One. 2012 Oct 31;7(10):e48138. doi: 10.1371/journal.pone.0048138 (PMC3485258; doi:10.1371/journal.pone.0048138)
Supplement: Figure S16 — NF-YA overexpression delays flowering time. (PDF) [file pone.0048138.s016.pdf]

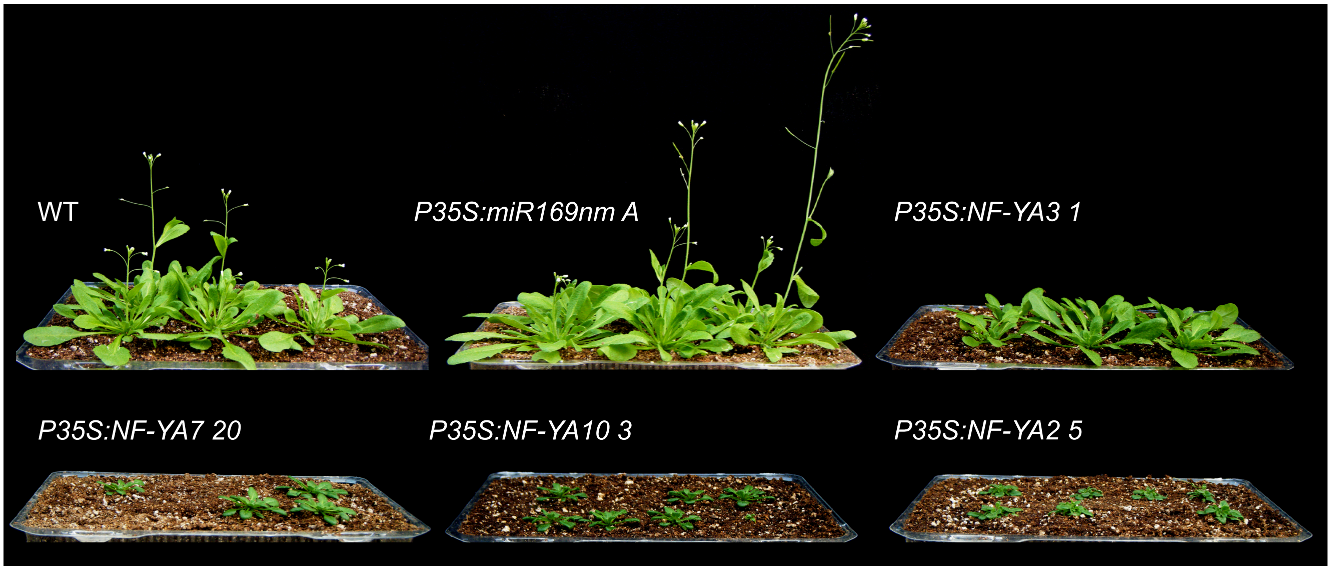

Figure S16. *NF-YA* overexpression delays flowering time.

Photographs of 6-week-old WT, *P35S:NF-YAs* and *P35S:miR169nm* plants grown in soil under standard light/dark conditions (16/8 hrs).
