# Supplementary material for: Functional and Transcriptome Analysis Reveals an Acclimatization Strategy for Abiotic Stress Tolerance Mediated by Arabidopsis NF-YA Family Members
Source: PLoS One. 2012 Oct 31;7(10):e48138. doi: 10.1371/journal.pone.0048138 (PMC3485258; doi:10.1371/journal.pone.0048138)
Supplement: Table S1 — Expression changes in genes involved in cell wall remodeling in PXVE:NF-YA transgenic lines. (PDF) [file pone.0048138.s017.pdf]

Table S1. Expression changes of genes involved in cell wall remodeling in *PXVE:NF-YA* transgenic lines

Gene ID	Description	Fold Change			
		PXVE:NF-YA2	***PXVE:NF-YA3***	***PXVE:NF-YA7***	***PXVE:NF-YA10***
	Xyloglucan endotransglucosylase-hydrolases (XTHs)				
At2g06850	XTH4	-	0.72	0.52	0.70
At5g13870	XTH5	0.73	0.66	0.56	-
At5g65730	XTH6	-	-	-	0.49
At1g11545	XTH8	-	-	0.61	-
At5g57530	XTH12	0.43	0.48	0.38	-
At4g25820	XTH14	0.44	-	0.33	-
At4g14130	XTH15	-	-	0.65	1.72
At3g23730	XTH16	-	-	1.60	-
At1g65310	XTH17	0.51	0.48	0.30	0.51
At4g30280	XTH18	0.27	0.22	0.17	0.25
At4g30290	XTH19	0.43	0.40	0.25	0.34
At5g48070	XTH20	-	-	0.47	-
At5g57560	XTH22	-	0.41	-	-
At4g25810	XTH23	0.28	0.32	0.34	0.35
At4g30270	XTH24	-	-	0.46	0.37
At4g28850	XTH26	0.37	-	0.15	-
At1g32170	XTH30	-	-	0.43	0.54
At1g10550	XTH33	0.59	-	0.35	-
	Expansins (EXPs)				
At3g45970	EXPL1 (EXP-like1)	0.31	0.43	0.18	0.38
At4g17030	EXLB1 (EXP-like B1)			0.64	0.63
At2g37640	EXP3	1.87		-	1.65
At2g28950	EXP6	-	-	1.44	-
At1g12560	EXP7	-	-	0.50	-
At2g40610	EXP8	-	-	0.25	-
At1g20190	EXP11	-	-	0.46	-
At5g56320	EXP14	0.45	-	0.46	-
	Extensins (EXTs)				
At1g21310	EXT3	0.64	-	-	-
At2g24980	EXT6	0.37	0.39	0.29	0.48
At5g06640	EXT10	0.39	-	0.34	-
At5g49080	EXT11	0.40	0.48	0.40	-
At5g05500	extension family protein	0.56	-	0.45	-
At3g09925	extension family protein	0.60	-	0.44	-
At4g02270	extension family protein	0.43	-	0.33	-
At2g34700	extension family protein	-	-	0.62	1.66
At5g15780	extension family protein	-	0.49	-	0.51
At3g28550	proline-rich extensin-like	0.34	0.37	0.36	0.52
At4g08400	proline-rich extensin-like	0.40	0.42	0.51	0.61
At2g43150	proline-rich extensin-like	0.74	0.71	0.74	0.64
At5g06630	proline-rich extensin-like	0.44	0.42	0.36	0.49
At3g54580	proline-rich extensin-like	0.34	0.37	0.26	0.52
At1g26250	proline-rich extensin-like	0.45	0.52	0.26	-
At4g08410	proline-rich extensin-like	0.44	0.41	0.45	-

Table S1. *Continued*

At3g54590	hydroxyproline-rich glycoprotein	0.41	0.47	0.42	-
Pectin methylesterases (PMEs), Pectinacetylesterases, Plant invertase/pectin methylesterase inhibitor					
At1g53840	PME1	-	0.73	0.57	0.53
At4g02330	PME41	0.19	0.35	-	-
At1g11580	Plant invertase/pectin methylesterase inhibitor	0.54	0.60	0.49	0.40
At5g04960	Plant invertase/pectin methylesterase inhibitor	0.40	0.55	0.41	-
At3g10720	Plant invertase/pectin methylesterase inhibitor	0.46	0.48	0.36	-
At2g45220	Plant invertase/pectin methylesterase inhibitor	-	-	0.42	0.44
At4g03930	Plant invertase/pectin methylesterase inhibitor	2.48	-	-	-
At2g26450	Plant invertase/pectin methylesterase inhibitor	-	2.42	-	-
At5g45280	pectinacetylesterase	0.71	0.67	0.60	0.68
At4g19420	pectinacetylesterase	3.10	2.11	-	-
At1g57590	pectinacetylesterase	-	-	-	1.78
Peroxidases (PRXs)					
At3g49110	PRX33	-	-	-	0.67
At4g08770	PRX37	-	-	0.54	-
At3g28200	Peroxidase superfamily	0.53	0.68	0.50	0.52
At4g33420	Peroxidase superfamily	0.70	0.69	0.66	-
At4g26010	Peroxidase superfamily	0.34	0.39	0.27	-
At5g39580	Peroxidase superfamily	0.19	-	0.26	0.29
At4g21960	Peroxidase superfamily	-	-	0.52	0.67
At5g64100	Peroxidase superfamily	-	-	0.61	0.66
At1g05250	Peroxidase superfamily	0.51	-	0.33	-
At1g05240	Peroxidase superfamily	0.47	-	0.31	-
At2g38380	Peroxidase superfamily	0.68		0.46	-
At2g37130	Peroxidase superfamily	-	-	0.51	0.58
At1g34510	Peroxidase superfamily	-	-	0.27	-
At4g08780	Peroxidase superfamily	-	-	0.60	-
At2g38390	Peroxidase superfamily	-	-	0.70	-
At4g11290	Peroxidase superfamily	-	-	0.57	-
At4g30170	Peroxidase superfamily	-	-	0.57	-
At5g51890	Peroxidase superfamily	-	-	0.46	-
At1g05260	Peroxidase superfamily	-	-	0.48	-
At5g19890	Peroxidase superfamily	-	-	-	1.38
At3g03670	Peroxidase superfamily	0.54	-	-	-

Gene expression values shown represent fold change (estradiol treatment vs. control), p-value $\leq$ 0.05. (–), not statistically significant.
