# Supplementary material for: Functional and Transcriptome Analysis Reveals an Acclimatization Strategy for Abiotic Stress Tolerance Mediated by Arabidopsis NF-YA Family Members
Source: PLoS One. 2012 Oct 31;7(10):e48138. doi: 10.1371/journal.pone.0048138 (PMC3485258; doi:10.1371/journal.pone.0048138)
Supplement: Table S2 — Sucrose content in WT, P35S:NF-YA2 and P35S:miR169nm lines. (PDF) [file pone.0048138.s018.pdf]

Table S2. Sucrose content in WT, *P35S:NF-YA2* and *P35S:miR169nm* lines

**Line**	**Sucrose**		
	μg/mg (FW)	SD	p-value
WT	0.211	0.023	-
P35S:NF-YA2 5	0.408	0.059	0.008
P35S:miR169nm A	0.168	0.020	0.038

Values are means of three biological replicates statistically treated using a student *t*-test. FW, fresh weight.
