# Supplementary material for: Functional and Transcriptome Analysis Reveals an Acclimatization Strategy for Abiotic Stress Tolerance Mediated by Arabidopsis NF-YA Family Members
Source: PLoS One. 2012 Oct 31;7(10):e48138. doi: 10.1371/journal.pone.0048138 (PMC3485258; doi:10.1371/journal.pone.0048138)
Supplement: Table S3 — Expression changes in genes belonging to the “anaerobic cluster” in PXVE:NF-YA and PXVE:miR169nm transgenic lines. (PDF) [file pone.0048138.s019.pdf]

Table S3. Expression changes in genes belonging to the "anaerobic cluster" in *PXVE:NF-YA* and *PXVE:miR169nm* transgenic lines

Gene ID	Description	Fold Change				
		PXVE:NF-YA2	***PXVE:NF-YA3***	***PXVE:NF-YA7***	***PXVE:NF-YA10***	***PXVE:miR169nm***
At5g10040	expressed protein	0.22317	0.09811	0.11024	0.07397	2.09783
At2g29870	major intrinsic family protein	0.37954	0.3791	0.39105	0.17861	1.21054
At3g43190	SUCROSE SYNTHASE 4	0.10395	0.07675	0.06754	0.05693	0.35669
At2g17850	Rhodanese	0.25176	0.11597	0.09108	0.106	-
At1g76650	CML38	0.43615	0.41809	0.11425	0.10695	6.29814
At2g47520	HRE2	0.1695	0.16804	0.19958	0.1251	-
At4g33070	Thiamine pyrophosphate dependent pyruvate decarboxylase family protein	0.14629	0.12665	0.09434	0.09013	-
At4g24110	unknown protein	0.40279	0.40762	0.21342	0.20524	3.87045
At5g66985	unknown protein	0.38725	0.37349	0.26893	0.19078	2.58875
At2g19590	ACC OXIDASE 1	0.3439	0.37716	0.24948	0.31233	-
At1g43800	stearoyl-acyl-carrier-protein desaturase family protein	0.28368	0.25078	0.16891	0.16119	0.73312
At5g54960	PYRUVATE DECARBOXYLASE-2	0.39217	0.38895	0.27441	0.4333	-
At1g77120	ALCOHOL DEHYDROGENASE 1	0.13504	0.18216	0.301	0.08186	-
At1g72360	HRE1	0.48747	0.41508	0.23147	0.42279	1.40734
At5g20830	SUCROSE SYNTHASE 1	0.23054	0.19257	0.29213	0.10124	0.74640
At2g26400	ACIREDUCTONE DIOXYGENASE 3	0.41522	0.25133	0.32435	0.12289	-
At5g59820	RESPONSIVE TO HIGH LIGHT 41; ZAT12	0.18838	0.19837	0.40414	0.1449	-
At3g29970	B12D protein	0.06913	0.06221	0.05111	0.02868	-
At4g17670	Protein of unknown function	0.39419	0.26216	0.26544	0.12544	1.77229
At4g26270	PHOSPHO-FRUCTOKINASE 3	0.46352	0.43708	0.44175	0.35603	-
At1g33055	unknown protein	0.39481	0.32415	0.10987	0.1584	5.68170
At3g03270	Adenine nucleotide alpha hydrolases-like superfamily protein	0.20211	0.17413	0.18209	0.11224	-
At1g35140	EXORDIUM LIKE 1	0.26044	0.34167	0.22758	0.16987	4.69267
At2g16060	HEMOGLOBIN 1	0.16934	0.17456	0.16508	0.11282	1.70533
At4g10270	Wound-responsive family protein	0.57032	0.55508	0.14103	0.18356	3.36846
At5g39890	Protein of unknown function	0.54404	0.52378	0.17417	0.21059	2.75164
At2g41730	unknown protein	0.13552	0.13359	0.29174	0.14330	-

Table S3. ***Continued***

At5g47910	RESPIRATORY BURST OXIDASE HOMOLOGUE D	0.44452	0.50561	0.35533	0.35664	1.52951
At3g23170	unknown protein	0.51171	0.61257	0.34552	0.23871	4.08399
At4g33560	Wound-responsive family protein	0.53068	0.55018	0.21328	0.31872	1.98021
At1g27730	SALT TOLERANCE ZINC FINGER STZ	0.39096	0.42403	-	0.35361	-
At5g51190	ERF (ethylene response factor) subfamily B-3	0.35777	0.42645	-	0.21060	-
At3g02550	LOB domain-containing protein 41	-	-	0.14631	0.16901	4.05369
At3g10040	sequence-specific DNA binding transcription factors	-	-	0.22902	0.22517	-
At3g27220	Galactose oxidase/ kelch repeat superfamily protein	-	-	0.17414	0.36557	-
At1g72940	Toll-Interleukin-Resistance (TIR) domain-containing protein	-	-	-	0.36265	1.88249
At5g54490	PINOID-BINDING PROTEIN 1	-	-	0.44680	-	1.75092
At2g22880	VQ motif-containing protein	-	-	-	-	2.15119
At3g23150	ETHYLENE RESPONSE 2	-	-	0.52852	-	-
At4g31020	alpha/beta-Hydrolases superfamily protein	2.02154	-	-	-	0.77878

Gene expression values shown represent fold change (estradiol treatment vs. control), p-value $\leq$ 0.05. (–), not statistically significant. “Anaerobic cluster” genes were taken from Licausi *et al*., 2010.
