# Supplementary material for: Functional and Transcriptome Analysis Reveals an Acclimatization Strategy for Abiotic Stress Tolerance Mediated by Arabidopsis NF-YA Family Members
Source: PLoS One. 2012 Oct 31;7(10):e48138. doi: 10.1371/journal.pone.0048138 (PMC3485258; doi:10.1371/journal.pone.0048138)
Supplement: Table S4 — Motifs enrichment in deregulated genes in estradiol treated PXVE transgenic lines. (PDF) [file pone.0048138.s020.pdf]

Table S4. Motifs enrichment in deregulated genes in estradiol treated PXVE transgenic lines

Line / expression	Motifs Identifier	No. Genes with this element	No. of elements	P value
PXVE:NF-YA10 / induced	Non-enriched			
PXVE:NF-YA10 / repressed	ABFs binding site	44	54	$< 10^{-5}$
	ABRE binding site	57	70	$< 10^{-5}$
	ABRE-like binding	228	392	$< 10^{-10}$
	ABREATRD22	39	42	$< 10^{-6}$
	ACGTABREMOTIFA2OSE	167	257	$< 10^{-10}$
	ATHB5ATCORE	37	76	$< 10^{-3}$
	AtMYC2 BS in RD22	254	358	$< 10^{-3}$
	CACGTGMOTIF	163	444	$< 10^{-10}$
	CARGCW8GAT	448	1488	$< 10^{-6}$
	CCA1 binding site	208	246	$< 10^{-3}$
	DRE core motif	165	192	$< 10^{-3}$
	GADOWNAT	105	139	$< 10^{-10}$
	GAREAT	390	573	$< 10^{-4}$
	GBF1/2/3 BS in ADH	22	44	$< 10^{-3}$
	GBOXLERBCS	38	46	$< 10^{-5}$
	MYB1AT	568	1354	$< 10^{-3}$
	MYCATERD1	254	358	$< 10^{-3}$
	TATA-box	631	1989	$< 10^{-10}$
	Motif TGA1 binding site	52	53	$< 10^{-10}$
	UPRMOTIFIAT	52	53	$< 10^{-10}$
	W-box promoter motif	482	880	$< 10^{-7}$
PXVE:NF-YA2 / induced	ABRE-like binding site motif	57	92	$< 10^{-3}$
PXVE:NF-YA2 / repressed	ABRE-like binding site motif	99	154	$< 10^{-6}$
	ACGTABREMOTIFA2OSEM	75	98	$< 10^{-5}$
	GADOWNAT	47	54	$< 10^{-4}$
	TATA-box Motif	304	967	$< 10^{-10}$
	TGA1 binding site motif	25	26	$< 10^{-5}$
	UPRMOTIFIAT	25	26	$< 10^{-5}$
	W-box promoter motif	233	460	$< 10^{-4}$
PXVE:NF-YA3 / induced	Non-enriched			
PXVE:NF-YA3 / repressed	ABRE-like binding site motif	91	141	$<10^{-4}$
	ACGTABREMOTIFA2OSEM	69	92	$< 10^{-4}$
	ATHB2 binding site motif	52	56	$< 10^{-3}$
	GADOWNAT	43	50	$< 10^{-3}$
	TATA-box Motif	291	926	$< 10^{-7}$
	W-box promoter motif	236	464	$< 10^{-6}$
PXVE:NF-YA7 / induced	CACGTGMOTIF	57	138	$< 10^{-3}$

Table S4. ***Continued***

PXVE:NF-YA7 / repressed	ABRE-like binding	175	260	$< 10^{-6}$
	ACGTABREMOTIFA2OSE	115	150	$< 10^{-3}$
	AtMYC2 BS in RD22	259	376	$< 10^{-7}$
	CARGCW8GAT	427	1424	$< 10^{-7}$
	GAREAT	360	549	$< 10^{-3}$
	MYB1AT	530	1237	$< 10^{-3}$
	MYCATERD1	259	376	$< 10^{-7}$
	RY-repeat promoter	34	72	$< 10^{-3}$
	TATA-box Motif	605	1991	$< 10^{-10}$
	TGA1 binding site	38	39	$< 10^{-4}$
	UPRMOTIFIAT	38	39	$< 10^{-4}$
	W-box promoter motif	474	877	$< 10^{-10}$
PXVE:NF-YA2SRDX / induced	EveningElement promoter motif	39	45	$< 10^{-6}$
	Ibox promoter motif	117	180	$< 10^{-4}$
PXVE:NF-YA2SRDX / repressed	ABFs binding site	43	52	$< 10^{-3}$
	ABRE binding site	58	71	$< 10^{-4}$
	ABRE-like binding	251	425	$< 10^{-10}$
	ABREATRD22	40	43	$< 10^{-5}$
	ACGTABREMOTIFA2OSE	183	275	$< 10^{-10}$
	CACGTGMOTIF	176	458	$< 10^{-10}$
	CARGCW8GAT	507	1678	$< 10^{-5}$
	DREB1A/CBF3	71	78	$< 10^{-3}$
	GADOWNAT	117	155	$< 10^{-10}$
	GAREAT	453	665	$< 10^{-5}$
	GBF1/2/3 BS in ADH	23	46	$< 10^{-3}$
	GBOXLERBCS	35	43	$< 10^{-3}$
	MYB1AT	662	1556	$< 10^{-6}$
	TATA-box Motif	726	2250	$< 10^{-10}$
	TGA1 binding site	51	51	$< 10^{-7}$
	UPRMOTIFIAT	51	51	$< 10^{-7}$
	W-box promoter motif	574	1123	$< 10^{-10}$
PXVE:miR169nm / induced	CARGCW8GAT	170	584	$< 10^{-3}$
	EveningElement promoter motif	42	50	$< 10^{-7}$
	GAREAT	156	245	$< 10^{-3}$
	Ibox promoter motif	129	189	$< 10^{-7}$
	TATA-box Motif	238	772	$< 10^{-7}$
	W-box promoter motif	184	366	$< 10^{-3}$
PXVE:miR169nm / repressed	Non-enriched			

Survey was performed in 1000 bp maximum upstream range cutting off at adjacent genes. Data obtained using the Athena Web tools (http://www.bioinformatics2.wsu.edu/cgi-bin/Athena/cgi/home.pl).
