# Supplementary material for: Functional and Transcriptome Analysis Reveals an Acclimatization Strategy for Abiotic Stress Tolerance Mediated by Arabidopsis NF-YA Family Members
Source: PLoS One. 2012 Oct 31;7(10):e48138. doi: 10.1371/journal.pone.0048138 (PMC3485258; doi:10.1371/journal.pone.0048138)
Supplement: Table S5 — Z-score for TATA-box and CCAAT-box motifs in the promoters of differentially expressed genes in PXVE transgenic lines. (PDF) [file pone.0048138.s021.pdf]

Table S5. Z-score for CCAAT-box and TATA-box in the promoters of differentially expressed genes in PXVE transgenic lines

**Line / expression**	**TATA-box**		**CCAAT**	
	Z-score	Significance	Z-score	Significance
PXVE:NF-YA2 / induced	2.3	0.05	1.4	-
PXVE:NF-YA2 / repressed	**5.9**	0.001	NF	-
PXVE:NF-YA7 / induced	1.3	-	0.9	-
PXVE:NF-YA7 / repressed	**7.1**	0.001	NF	-
PXVE:NF-YA10 / induced	0.8	-	0.5	-
PXVE:NF-YA10 / repressed	**2.6**	0.005	NF	-
PXVE:NF-YA3 / induced	0.6	-	1.9	0.05
PXVE:NF-YA3 / repressed	**4.5**	0.001	NF	-
PXVE:NF-YAs / induced	-0.5	-	0.2	-
PXVE:NF-YAs / repressed	**2.9**	0.005	0.8	-
PXVE:miR169nm / induced	**3.9**	0.001	NF	-
PXVE:miR169nm / repressed	1.2	-	NF	-
PXVE:NF-YA2SRDX / induced	1.9	0.05	NF	-
PXVE:NF-YA2SRDX / repressed	2.2	-	3.5	-

Survey was performed in TAIR7 upstream 500 sequences. Data obtained using the Promomer web tool (at http://bbc.botany.utoronto.ca). NF not found in at least 50% of the tested genes. (-), Not shown by the program. Significant differences, based on a Z-score, are highlighted in bold. TATA-box was used as a control to compare the CCAAT-box with another widely distributed promoter element.
