# Supplementary material for: Functional and Transcriptome Analysis Reveals an Acclimatization Strategy for Abiotic Stress Tolerance Mediated by Arabidopsis NF-YA Family Members
Source: PLoS One. 2012 Oct 31;7(10):e48138. doi: 10.1371/journal.pone.0048138 (PMC3485258; doi:10.1371/journal.pone.0048138)
Supplement: Table S6 — Motifs enrichment in the promoters of repressed genes in PXVE:NF-YA2SRDX lines that change in its PXVE:NF-YA2 counterpart, putative indirect targets. (PDF) [file pone.0048138.s022.pdf]

Table S6. Motifs enrichment in the promoters of repressed genes in *PXVE:NF-YA2SRDX* lines that change in its *PXVE:NF-YA2* counterpart

Line / expression	Motifs Identifier	No. Genes with this element	No. of elements	P value
PXVE:NF-YA2 / induced	Non enriched			
PXVE:NF-YA2 / repressed	ABRE-like binding site motif	50	77	$< 10^{-3}$
	TATA-box Motif	151	462	$< 10^{-5}$
	TGA1 binding site motif	14	14	$< 10^{-3}$
	UPRMOTIFIAT	14	14	$< 10^{-3}$
	W-box promoter motif	120	266	$< 10^{-3}$

Survey was performed in 1000 bp maximum upstream range cutting off at adjacent genes. Data obtained using the Athena Web tools (http://www.bioinformatics2.wsu.edu/cgi-bin/Athena/cgi/home.pl).
