# Supplementary material for: Functional and Transcriptome Analysis Reveals an Acclimatization Strategy for Abiotic Stress Tolerance Mediated by Arabidopsis NF-YA Family Members
Source: PLoS One. 2012 Oct 31;7(10):e48138. doi: 10.1371/journal.pone.0048138 (PMC3485258; doi:10.1371/journal.pone.0048138)
Supplement: Table S7 — Z-score for TATA-box and CCAAT-box motifs in the promoters of repressed genes in PXVE:NF-YA2SRDX line that change in its PXVE:NF-YA2 counterpart, putative indirect targets. (PDF) [file pone.0048138.s023.pdf]

**Table S7.** Z-score for CCAAT-box and TATA-box in the promoters of repressed genes in *PXVE:NF-YA2SRDX* lines that change in its *PXVE:NF-YA2* counterpart

| **Line / expression** | **TATA-box** | | **CCAAT** | |
|---|---|---|---|---|
| | Z-score | Significance | Z-score | Significance |
| *PXVE:NF-YA2* / induced | -0.3 | - | **3.1** | 0.001 |
| *PXVE:NF-YA2* / repressed | **3.0** | 0.005 | 1.4 | - |

Survey was performed in TAIR7 upstream 500 sequences. Data obtained using the Promomer web tool (at http://bbc.botany.utoronto.ca). NF not found at least in 50 % of the tested genes. (-), Not shown by the program. Significant differences, based on a Z-score, are highlighted in bold. TATA-box was used as a control to compare the CCAAT-box with another widely distributed promoter element.
