# Supplementary material for: Functional and Transcriptome Analysis Reveals an Acclimatization Strategy for Abiotic Stress Tolerance Mediated by Arabidopsis NF-YA Family Members
Source: PLoS One. 2012 Oct 31;7(10):e48138. doi: 10.1371/journal.pone.0048138 (PMC3485258; doi:10.1371/journal.pone.0048138)
Supplement: Table S8 — Sequences of primers and probes used in this study. (PDF) [file pone.0048138.s024.pdf]

Table S8. Sequences of primers and probes used in this study

Gene	Probe	Sequence (5' to 3')
miR169	miR169a	TCGGCAAGTCATCCTTGGCTG
	miR169hn	CAGGCAAGTCATCCTTGGCTA
U6sn	U6	GTCATCCTTGCGCAGGGGCCATGCTAAT
miR156	miR156g	GTGTGCTCACTCTCTTCTGTCG

Gene	Primer set Sequence (5' to 3')	Usage
miR164abc	**Stem-loop primer**<br>GTCGTATCCAGTGCAGGGTCCGAGGTATTC<br>GCACTGGATACGACCGCACG<br>**Forward**<br>CACGTGGAGAAGCAGGGCA<br>**miRRev**<br>CCAGTGCAGGGTCCGAGGT	qRT-PCR
mir169ag	**Stem-loop primer**<br>GTCGTATCCAGTGCAGGGTCCGAGGTATTC<br>GCACTGGATACGACTCGGCA<br>**Forward**<br>TCACGCAGCCAAGGATGACTT<br>**miRRev**<br>CCAGTGCAGGGTCCGAGGT	qRT-PCR
miR169hn	**Stem-loop primer**<br>GTCGTATCCAGTGCAGGGTCCGAGGTATTC<br>GCACTGGATACGACCAGGCA<br>**Forward**<br>TGCACGTAGCCAAGGATGACTT<br>**miRRev**<br>CCAGTGCAGGGTCCGAGGT	qRT-PCR
miR169a	**Forward**<br>GATTGACCTCTCTCTCTCCA<br>**Reverse**<br>CTTTCGTCACATTCGCGTTTC	Promoter:GUS fusion
[a]miR169m/n	**Forward**<br>***GGGGACAAGTTTGTACAAAAAAGCAGG***<br>***CTAA***GTGTGAGACTTTTGCCGTTTTTC<br>**Reverse**<br>***GGGGACCACTTTGTACAAGAAAGCTGG***<br>***GTA***GACGCTTTCCGCCATGTTAGACCTC	Promoter:GUS fusion
[a]miR169k/l	**Forward**<br>***GGGGACAAGTTTGTACAAAAAAGCAGG***<br>***CTAA***CAAGCGATTGTGGAAGAAC<br>**Reverse**<br>***GGGGACCACTTTGTACAAGAAAGCTGG***<br>***GTA***GACGCTTTCCGCCATGTTAGACCTC	Promoter:GUS fusion
[a]miR169i/j	**Forward**<br>***GGGGACAAGTTTGTACAAAAAAGCAGG***<br>***CTAA***GTATGGTTCCGATGCAGCGCAC<br>**Reverse**<br>***GGGGACCACTTTGTACAAGAAAGCTGG***<br>***GTA***GACGCTTTCCGCCATGTTAGACCTC	Promoter:GUS fusion

Table S8. ***Continued***

[a]miR169h	**Forward**<br>***GGGGACAAGTTTGTACAAAAAAGCAGGCTAA***GTGGGATGGGCTAAAAGCCAC<br>**Reverse**<br>***GGGGACCACTTTGTACAAGAAAGCTGGGTA***GTTGTTGATGAGTATCCCAC	Promoter:GUS fusion
[a]miR169nm	**Forward**<br>***GGGGACAAGTTTGTACAAAAAAGCAGGCTAA***GAAGGAGCATATTCCCGTGATG<br>**Reverse**<br>***GGGGACCACTTTGTACAAGAAAGCTGGGTA***GGAAGATGCGAGACGAGACGTG	Overexpressing line
[a]At3g05690	**Forward**<br>***GGGGACAAGTTTGTACAAAAAAGCAGGCTAA***GAGGAGACGAATCAGACTG<br>**Reverse**<br>***GGGGACCACTTTGTACAAGAAAGCTGGGTA***GATATGTTCCTGTGGAAG	Promoter:GUS fusion
[a]At1g30500	**Forward**<br>***GGGGACAAGTTTGTACAAAAAAGCAGGCTAA***CACGTTGAAGTCTATTGAGCTC<br>**Reverse**<br>***GGGGACCACTTTGTACAAGAAAGCTGGGTA***CATGAGCTTAATGTCCGAAG	Promoter:GUS fusion
[a]At1g54160	**Forward**<br>***GGGGACAAGTTTGTACAAAAAAGCAGGCTAA***GTGGTGGCACAAGAGAAGGTC<br>**Reverse**<br>***GGGGACCACTTTGTACAAGAAAGCTGGGTA***GTCCAAACCAACAAGTCCAG	Promoter:GUS fusion
[a]At1g72830	**Forward**<br>***GGGGACAAGTTTGTACAAAAAAGCAGGCTAA***CTGGTGCGGAGATCAATTC<br>**Reverse**<br>***GGGGACCACTTTGTACAAGAAAGCTGGGTA***GCTAATAATCCAAGTGGGTG	Promoter:GUS fusion
[a]At5g06510	**Forward**<br>***GGGGACAAGTTTGTACAAAAAAGCAGGCTAA***CTGCCGTCAAAGTTGACATGAC<br>**Reverse**<br>***GGGGACCACTTTGTACAAGAAAGCTGGGTA***GCTTATGTTCCAAAAGTCCAATG	Promoter:GUS fusion
[b]At3g05690	**Forward**<br>TGGAACCGAGATGGCTTCCT<br>**Reverse**<br>**TTA**AGCGAAACCCAAACGGAGTTCTAGATCCAGATCCAGGGTTTTGAAATTGCAGCAGCCA	Chemeric repressor version
At5g06510	**Forward**<br>GGAACCGACATAGCTCCCAAC<br>**Reverse**<br>TCATATATTAAGTTTGCAGCAGC	Overexpressing line

Table S8. ***Continued***

[a]At1g72830	**Forward**<br>***GGGGACAAGTTTGTACAAAAAAGCAGGCTAA***CTGTGTCAGGGAATTTAGGA<br>**Reverse**<br>***GGGGACCACTTTGTACAAGAAAGCTGGGTA***CAGCACCAAGATCCACTGTC	Overexpressing line
At1g30500	**Forward**<br>GAGGCTCTCTAATCGTCGTCAG<br>**Reverse**<br>GATGATCTGAGACCAAACAGAGCC	Overexpressing line
At3g05690	**Forward**<br>CGCACTTAGCTTTCTCACTTG<br>**Reverse**<br>TGATGCATATATGGCTTGCGGCATC	qRT-PCR
At5g06510	**Forward**<br>CACTTTCTCACCTGGTGGTG<br>**Reverse**<br>CATATATGGCTTACGGCATC	qRT-PCR
At1g30500	**Forward**<br>GTCACGTAAGCCGTATTTGCATGA<br>**Reverse**<br>GATGATCTGAGACCAAACAGAGCC	qRT-PCR
At1g72830	**Forward**<br>CTCCTGCACAGGCTAATTTC<br>**Reverse**<br>CGTGGTATTGCTTCGCATTG	qRT-PCR
At1g54160	**Forward**<br>CCAACGGCGCTGCCACATTTAG<br>**Reverse**<br>ATGAAGGTACGGTTTACGGC	qRT-PCR
COR15B	**Forward**<br>ATGACCTCAACGAAGCCACA<br>**Reverse**<br>TGGCTTCAATGGTTTTCTCAA	qRT-PCR
RD29A	**Forward**<br>GTTACTGATCCCACCAAAGAAGA<br>**Reverse**<br>GGAGACTCATCAGTCACTTCCA	qRT-PCR
RD29B	**Forward**<br>GCGCACCAGTGTATGAATCC<br>**Reverse**<br>CTTTTCGCTTCCCAGTCCGATG	qRT-PCR
EXL1	**Forward**<br>TGGATCGGCTTGTACTGGAG<br>**Reverse**<br>GGGTCAAACAAAGCCGGTAA	qRT-PCR
…………………	**Reverse**<br>AGCGAAACCCAAACGGAGTTCTAG	qRT-PCR SRDX

[a]ATTB´s sequence are in bold and cursive. [b]Artificial stop codon is in bold and SRDX coding sequence is underlined.
